# Supplementary material for: Compact engineered human mechanosensitive transactivation modules enable potent and versatile synthetic transcriptional control
Source: Nat Methods. 2023 Oct 9;20(11):1716–28. doi: 10.1038/s41592-023-02036-1 (PMC10630135; doi:10.1038/s41592-023-02036-1)

nature methods

Article

https://doi.org/10.1038/s41592-023-02036-1

# Compact engineered human mechanosensitive transactivation modules enable potent and versatile synthetic transcriptional control

In the format provided by the authors and unedited

nature methods

Article

https://doi.org/10.1038/s41592-023-02036-1

# Compact engineered human mechanosensitive transactivation modules enable potent and versatile synthetic transcriptional control

In the format provided by the authors and unedited

## Supplementary Information

# Compact engineered human mechanosensitive transactivation modules enable potent and versatile synthetic transcriptional control

Barun Mahata[1], Alan Cabrera[1], Daniel A. Brenner[1], Rosa Selenia Guerra-Resendez[2], Jing Li[1], Jacob Goell[1], Kaiyuan Wang[1], Yannie Guo[1], Mario Escobar[3], Abinand Krishna Parthasarathy[1], Hailey Szadowski[2], Guy Bedford[1], Daniel Reed[1], Sunghwan Kim[1], and Isaac B. Hilton[1,2,3*]

[1] Department of Bioengineering, Rice University, Houston, TX, USA
[2] Systems, Synthetic, and Physical Biology Graduate Program, Rice University, Houston, TX, USA
[3] Department of BioSciences, Rice University, Houston, TX, USA

* To whom correspondence should be addressed. Tel: 713-348-8247; Email: isaac.hilton@rice.edu

## Table of Contents

**Supplementary Notes 1 – 6:** Pages 2 – 9

**Supplementary References:** Pages 9 – 10

**Supplementary Figures 1 – 35:** Pages 11 – 53

**Supplementary Tables 1 – 8:** Pages 54 – 63

**Supplementary Note 7:** Pages 64 – 73

**Uncropped Western blots from Supplementary Figures:** Page 74

## Supplementary Note 1: Select TADs from MTFs can activate transcription from diverse endogenous human loci when recruited by dCas9

We first isolated TADs from 7 different serum-responsive MTFs (YAP, YAP-S397A[1], TAZ, SRF, MRTF-A, MRTF-B, and MYOCD) and analyzed their ability to activate transcription when recruited to human promoters using either N- or C-terminal fusion to *Streptococcus pyogenes* dCas9 (dCas9), SunTag-mediated recruitment[2], or recruitment via a gRNA aptamer and fusion to the MCP protein[3] (**Supplementary Fig. 1**). TADs derived from MRTF-A, MRTF-B, or MYOCD displayed consistent transactivation potential across recruitment architectures. We next compared the optimal recruitment strategies for MRTF-A and MRTF-B TADs because they were more potent than, or comparable to, the MYOCD TAD, yet slightly smaller. Our results demonstrated that TADs from MRTF-A and B functioned best when fused to the MCP protein and recruited via gRNA aptamers (**Supplementary Fig. 2**), and further that this strategy could be used with pools or single gRNAs, and to activate enhancer RNAs (eRNAs) and long noncoding RNAs (lncRNAs).

Although the NRF2-ECH homology domains 4 and 5 (Neh4 and Neh5, respectively) within the oxidative stress/antioxidant regulated NRF2 human MTF have been shown to activate gene expression in Gal4 systems[4], we observed that neither Neh4 nor Neh5 were capable of potent human gene activation when recruited to promoters in any dCas9-based architecture (**Supplementary Fig. 3**). Therefore, we constructed an engineered TAD called eNRF2, consisting of Neh4 and Neh5 separated by an extended glycine-serine linker and found that the eNRF2 TAD stimulated high levels of transactivation in all dCas9-based recruitment configurations (**Supplementary Fig. 3**). Similar to the MRTF-A/B TADs, eNRF2 displayed optimal potency in the gRNA aptamer/MCP-based recruitment architecture and transactivated diverse human regulatory loci (**Supplementary Fig. 4**). We next tested whether TADs derived from one of 6 different cytokine regulated/JAK-STAT family MTFs (STAT1 – 6) could transactivate human genes but observed that single STAT TADs alone were incapable of potent transactivation regardless of dCas9-based recruitment context (**Supplementary Fig. 5**). Interestingly, all native TADs that displayed measurable efficacy at endogenous target sites harbored at least one 9aa segment that perfectly matched predictions generated by previously described software[5] (**Supplementary Fig. 6**). Together, these data demonstrate that TADs from human MTFs can transactivate human loci when recruited via dCas9 and that these TADs are amenable to protein engineering.

**Supplementary Note 2: Combinations of TADs from MTFs can potently activate human genes when recruited by dCas9**

STAT proteins typically activate gene expression in combination with co-factors[6]. Therefore, we tested if TADs from different STAT proteins might synergize with other MTF TADs. We built 24 different bipartite fusion proteins by linking each STAT TAD to the N- or C- terminus of either the MRTF-A or MRTF-B TAD and then assayed the relative transactivation potential of each bipartite fusion when recruited to the human *OCT4* promoter using gRNA aptamer/MCP-based recruitment (**Supplementary Fig. 7a – c**). All of these 24 fusions markedly outperformed single TADs from MRTF-A/B or STAT alone, and one bipartite TAD configuration (MRTF-A/STAT1) was comparable to MCP fused to the dCas9-SAM derived bipartite p65-HSF1[3] module. Similar results were obtained at other human promoters (**Supplementary Fig. 7d and e**).

We next investigated whether the eNRF2 TAD could further enhance the potency of the MRTF-A/STAT1 module by building tripartite fusions consisting of MRTF-A/STAT1/eNRF2 (MSN) or eNRF2/MRTF-A/STAT1 (NMS) TADs. Both MSN and NMS stimulated *OCT4* mRNA synthesis to levels comparable to the state-of-the-art CRISPRa platforms (**Supplementary Fig. 8a and b**) when recruited to the *OCT4* promoter using gRNA aptamers/MCP-based targeting. We further validated these results against dCas9 + MCP-p65-HSF1 at six other endogenous promoters (**Supplementary Fig. 8c and d**). Notably, switching the order of p65 and HSF1 did not improve the efficacy of the p65-HSF1 TAD module (**Supplementary Fig. 8e and f**). Surprisingly, the potency of the MSN tripartite effector was not further enhanced by the direct fusion of other TADs to the C-terminus of dCas9 (**Supplementary Fig. 8g**). Collectively, these data show that gRNA aptamer/MCP-based recruitment of the MSN or NMS modules – termed the CRISPR-dCas9 recruited enhanced activation module (DREAM) platform – can efficiently stimulate transcription without viral components. Our results also demonstrate that natural and engineered human TADs can have non-obvious interactions when combinatorially recruited in bi- and tripartite fashions.

## Supplementary Note 3: 9aa TAD combinations from select MTFs efficiently activate endogenous transcription

Although single 9aa TADs derived from MRTF-A, MRTF-B or MYOCD were unable to substantially induce transcription (**Supplementary Fig. 22d**), we hypothesized that the combinatorial fusion of multiple 9aa TADs could do so. To test this hypothesis, we generated heterotypic bipartite 9aa TAD fusions derived from full-length TADs separated by a 1x glycine-serine linker. Our data showed that fusion of two distinct 9aa TADs from MYOCD.1 and MYOCD.3 respectively, modestly induced transcription (**Supplementary Fig. 22e**). We used this bipartite fusion for further testing. We fused different 9aa TADs derived from MRTF-A or MRTF-B to the N-terminus or C-terminus of the 2x 9aa TAD MYOCD.1-GS-MYOCD.3, and interestingly observed that all heterotypic 3x 9aa TADs strongly induced transcription, with a 3x 9aa TAD configuration of MRTF-B.3-GS-MYOCD.1-GS-MYOCD.3 (**Main text Fig. 3g)** displaying the highest transcriptional induction from our *OCT4* testbed locus (**Supplementary Fig. 22f**). To assess the effectiveness of this 3x 9aa TAD more generally, we targeted endogenous protein coding genes using pooled gRNAs (*HBG1*, *TTN and CD34*) and single gRNAs (*SBNO2*). We also targeted this 3x 9aa TAD, to the GRASLND lncRNA and the *OCT4-DE* enhancer using pools of gRNAs and observed substantial transactivation at all tested loci (**Main text Fig. 3h and Supplementary Fig. 22g – j**).

## Supplementary Note 4: Fusion of VP64 boosts the performance of MSN and NMS

Direct fusion of effectors to dCas9 is attractive because it enables less complex delivery. However, many effectors display optimal performance when recruited by dCas9 using other architectures (e.g., SunTag/MCP; **Supplementary Figs. 2a and b, 4a and b and 8b**). That is, some effectors appear to lose efficacy when directly fused to dCas9. Therefore, we sought to test the relative efficacy of MSN and NMS in direct dCas9 fusion formats relative to similar directly fused architectures such as dCas9-VPR. Although, direct fusion of MSN or NMS to the C- or N-terminus of dCas9 enabled robust activation of target genes, efficacy with any of these formats remained lower than that of dCas9-VPR (**Supplementary Fig. 26a**). To test if we could further boost the potency of MSN/NMS direct fusions to dCas9, we added the VP64 domain in various locations and, interestingly observed highly potent transcriptional activation comparable to that of dCas9-VPR at the *OCT4* testbed locus. Moreover, the size of the effector components in the best performing fusion protein; NMS-dCas9-VP64 (1,020bp) is ~35% smaller than the size of the VPR effector (1,569bp) in dCas9-VPR (**Supplementary Fig. 26d**). We also noted that the relative expression of NMS-dCas9-VP64 was qualitatively higher than dCas9-VPR in HEK293T cells using Western blotting (**Supplementary Fig. 26b**). Moreover, NMS-dCas9-VP64 performed significantly better than dCas9-VP192 and VP64-dCas9-VP64 (**Supplementary Fig. 26c**). NMS-dCas9-VP64 performed comparably to dCas9-VPR at a battery of endogenous loci in HEK293T cells using pool of gRNAs and single gRNAs (**Supplementary Fig. 26e and f,** respectively).

**Supplementary Note 5: The NMS effector enables superior multiplexed gene activation when fused to dCas12a**

The CRISPR/Cas12a system has attracted significant attention because the platform is smaller than SpCas9, and because Cas12a can process its own crRNA arrays in human cells[7]. This feature has been leveraged for both multiplexed genome editing and multiplexed transcriptional control[8]. Therefore, we next investigated the potency of the tripartite MSN and NMS effectors when they were directly fused to dCas12a (**Main text Fig. 4f**). We selected the AsdCas12a variant for this analysis because AsdCas12a (hereafter dCas12a) has been shown to activate human genes when fused to transcriptional effectors[8]. Our results demonstrated that both dCas12a-MSN and dCas12a-NMS were able to induce gene expression when targeted to different human promoters using pooled or single crRNAs (**Main text Fig. 4g and h**, **Supplementary Fig. 30a – e**). dCas12a-NMS was generally superior to dCas12a-MSN and to the previously described dCas12a-Activ system[8] at the loci tested here. These data demonstrate that the NMS and MSN effectors domains are potent transactivation modules when combined with the dCas12a targeting system in human cells.

We next tested the extent to which dCas12a-MSN/NMS could be used in conjunction with crRNA arrays for multiplexed endogenous gene activation. We cloned 8 previously described crRNAs[8] (targeting the *ASCL1, IL1R2, IL1B* or *ZFP42* promoters) into a single plasmid in an array format and then transfected this vector into HEK293T cells with either dCas12a control, dCas12a-MSN, dCas12a-NMS, or the dCas12a-Activ system. Again, our data demonstrated that dCas12a-NMS was superior or comparable to dCas12a-Activ, even in multiplex settings (**Supplementary Fig. 30f**). Finally, to evaluate if dCas12-NMS could simultaneously activate multiple genes on a larger scale, we cloned 20 full-length (20bp) crRNAs targeting 16 different loci into a single array (**Supplementary Fig. 30g**). This array was designed to enable simultaneous targeting of several classes of human regulatory elements; including 13 different promoters, 2 different enhancers (one intrageneric; *SOCS1*, and one driving eRNA output; *NET1*), and one lncRNA (GRASLND). When this crRNA array was transfected into HEK293T cells along with dCas12a-NMS, RNA synthesis was robustly stimulated from all 16 loci (**Main text Fig. 4i**). To our knowledge this is the most loci that have been targeted simultaneously using CRISPR systems, demonstrating the versatility and utility of the engineered NMS effector in combination with dCas12a.

## Supplementary Note 6: Molecular cloning details

### Plasmid Cloning

Lenti-dCas9-VP64 (Addgene #61425), dCas9-VPR (Addgene #63798), dCas9-p300 (Addgene #83889), MCP-p65-HSF1 (Addgene #61423), scFv-VP64 (Addgene #60904), SpgRNA expression plasmid (Addgene #47108), MS2-modified gRNA expression plasmid (Addgene #61424), AsCas12a (Addgene #128136), *E. Coli* Type I Cascade system (Addgene #106270-106275) and *Pae* Type I Cascade System (Addgene #153942 and 153943), YAP-S5A (Addgene #33093) have been described previously. The eNRF2 TAD fusion was synthetically designed and ordered as a gBlock from IDT. To generate an isogenic C-terminal effector domain cloning backbone, the dCas9-p300 plasmid (Addgene #83889) was digested with BamHI and then a synthetic double-stranded ultramer (IDT) was incorporated using NEBuilder HiFi DNA Assembly (NEB, E2621) to generate a dCas9-NLS-linker-BamHI-NLS-FLAG expressing plasmid. This plasmid was further digested with AfeI and then a synthetic double-stranded ultramer (IDT) was incorporated using NEBuilder HiFi DNA Assembly to generate a FLAG-NLS-MCS-linker-dCas9 expressing Plasmid for N-terminal effector domain cloning. For fusion of effector domains to MCP, the MCP-p65-HSF1 plasmid (Addgene #61423) was digested with BamHI and NheI and respective effector domains were cloned using NEBuilder HiFi DNA Assembly. For SunTag components, the scFv-GCN4-linker-VP16-GB1-Rex NLS sequence was PCR amplified from pHRdSV40-scFv-GCN4-sfGFP-VP64-GB1-NLS (Addgene #60904) and cloned into a lentiviral backbone containing an EF1-alpha promoter. Then VP64 domain was removed and an AfeI restriction site was generated and used for cloning TADs using NEBuilder HiFi DNA Assembly. The pHRdSV40-dCas9-10xGCN4_v4-P2A-BFP (Addgene #60903) vector was used for dCas9-based scFv fusion protein recruitment to target loci. All MTF TADs were isolated using PCR amplified from a pooled cDNA library from HEK293T, HeLa, U2OS and Jurkat-T cells. TADs were cloned into the MCP, dCas9 C-terminus, dCas9 N-terminus, and scFv backbones described above using NEBuilder HiFi DNA Assembly. Bipartite N-terminal fusions between MCP-MRTF-A or MCP-MRTF-B TADs and STAT 1-6 TADs were generated by digesting the appropriate MCP-fusion plasmid (MCP-MRTF-A or MCP-MRTF-B) with BamHI and then subcloning PCR-amplified STAT 1-6 TADs using NEBuilder HiFi DNA Assembly. Bipartite C-terminal fusions between MCP-MRTF-A or MCP-MRTF-B TADs and STAT 1-6 TADs were generated by digesting the appropriate MCP-fusion plasmid (MCP-MRTF-A or MCP-MRTF-B) with NheI and then subcloning PCR-amplified STAT 1-6 TADs using NEBuilder HiFi DNA Assembly. Similarly, eNRF2 was fused to the N- or C-terminus of the bipartite MRTF-A-STAT1 TAD in the MCP-fusion backbone using either BamHI (N-terminal; MCP-eNRF2-MRTF-A-STAT1 TAD) or NheI (C-terminal; MCP-MRTF-A-STAT1-eNRF2 TAD) digestion and NEBuilder HiFi DNA Assembly to generate the MCP-NMS or MCP-MSN tripartite TAD fusions, respectively. SadCas9 (with D10A and N580A mutations derived using PCR) was PCR amplified and then cloned into the SpdCas9 expression plasmid backbone created in this study digested with BamHI and XbaI. This SadCas9 expression plasmid was digested with BamHI and then PCR-amplified VP64 or VPR TADs were cloned in using NEBuilder HiFi DNA Assembly. CjCas9 was PCR-amplified from pAAV-EFS-CjCas9-eGFP-HIF1a (Addgene #137929) as two overlapping fragments using primers to create D8A and H559A mutations. These two CjdCas9 PCR fragments were then cloned into the SpdCas9 expression plasmid digested with BamHI and XbaI using

NEBuilder HiFi DNA Assembly. This CjdCas9 expression plasmid was digested with BamHI and the PCR-amplified VP64 or VPR TADs were cloned in using NEBuilder HiFi DNA Assembly. HNH domain deleted SpdCas9 plasmids were generated using different primer sets designed to amplify the N-terminal and C-terminal portions of dCas9 excluding the HNH domain and resulting in either: no linker, a glycine-serine linker, or an XTEN16 linker, between HNH-deleted SpdCas9 fragments. These different PCR-amplified regions were cloned into the SpdCas9 expression plasmid digested with BamHI and XbaI using NEBuilder HiFi DNA Assembly. MCP-mCherry, MCP-MSN and MCP-p65-HSF1 were digested with NheI and a single strand oligonucleotide encoding the FLAG sequence was cloned onto the C-terminus of each respective fusion protein using NEBuilder HiFi DNA Assembly to enable facile detection via Western blotting. 1x 9aa TADs were designed and annealed as double strand oligos and then cloned into the BamHI/NheI-digested MCP-p65-HSF1 backbone plasmid (Addgene #61423) using T4 ligase (NEB). Heterotypic 2x 9aa TADs were generated by digesting MCP-1x 9aa TAD plasmids with either BamHI or NheI and then cloning single strand DNA encoding 1x 9aa TADs to the N- or C-termini using NEBuilder HiFi DNA Assembly. Heterotypic MCP-3x 9aa TADs were generated similarly by digesting MCP-2x 9aa TAD containing plasmids either with BamHI or NheI and then single strand DNA encoding 1x 9aa TADs were cloned to the N- or C-termini using NEBuilder HiFi DNA Assembly. Selected fusions between 3x 9aa TADs and eNRF2 were generated using gBlock (IDT) fragments and cloned into the BamHI/NheI-digested MCP-p65-HSF1 backbone plasmid (Addgene #61423) using NEBuilder HiFi DNA Assembly. To generate mini-DREAM compact single plasmid system, SpdCas9-HNH (no linker) deleted plasmid was digested with BamHI and then PCR amplified P2A self-cleaving sequence and MCP-eNRF2-3x 9aa TAD (eN3x9) was cloned using NEBuilder HiFi DNA Assembly. For dCas12a fusion proteins, SiT-Cas12a-Activ (Addgene #128136) was used. First, we generated a nuclease dead (E993A) SiT-Cas12a backbone using PCR amplification and we used this plasmid for subsequent C-terminal effector cloning using BamHI digestion and NEBuilder HiFi DNA Assembly. For *E. coli* Type I CRISPR systems, the Cas6-p300 plasmid (Addgene #106275) was digested with BamHI and then MSN and NMS domains were cloned in using NEBuilder HiFi DNA Assembly. *Pae* Type I Cascade plasmids encoding Csy1-Csy2 (Addgene #153942) and Csy3-VPR-Csy4 (Addgene #153943) were obtained from Addgene. The Csy3-VPR-Csy4 plasmid was digested with MluI (NEB) and BamHI (to remove the VPR TAD) and then the nucleoplasmin NLS followed by a linker sequence was added using NEBuilder HiFi DNA Assembly. Next, this Csy3-Csy4 plasmid was digested with AscI and either the MSN or NMS TADs were cloned onto the N-terminus of Csy3 NEBuilder HiFi DNA Assembly. ZF fusion proteins were generated by cloning PCR-amplified MSN, NMS, or VPR domains into the BsiWI and AscI digested *ICAM1* targeting ZF-p300 plasmid[9] using NEBuilder HiFi DNA Assembly. Similarly, TALE fusion proteins were created by cloning PCR-amplified MSN, NMS, or VPR domains into the BsiWI and AscI digested *IL1RN* targeting TALE plasmid backbone[9] using NEBuilder HiFi DNA Assembly. pCXLE-dCas9VP192-T2A-EGFP-shP53 (Addgene #69535), GG-EBNA-OSK2M2L1-PP (Addgene #102898) and GG-EBNA-EEA-5guides-PGK-Puro (Addgene #102898) used for reprogramming experiments have been described previously[10, 11]. The PCR-amplified NMS domain was cloned into the sequentially digested (XhoI then SgrDI; to remove the VP192 domain) pCXLE-dCas9VP192-T2A-EGFP-shP53 backbone using NEBuilder HiFi

DNA Assembly. TADs were directly fused to the C-terminus of dCas9 by digesting the dCas9-NLS-linker-BamHI-NLS-FLAG plasmid with BamHI and then cloning in PCR-amplified TADs using NEBuilder HiFi DNA Assembly. TADs were directly fused to the N-terminus to dCas9 by digesting the FLAG-NLS-MCS-linker-dCas9 plasmid with AgeI (NEB) and then cloning in PCR-amplified TADs using NEBuilder HiFi DNA Assembly. For constructs harboring both N- and C-terminal fusions, respective plasmids with TADs fused to the C-terminus of dCas9 were digested with AgeI and then PCR-amplified TADs were cloned onto the N-terminus of dCas9 using NEBuilder HiFi DNA Assembly. hSyn-AAV-EGFP (Addgene #50465) plasmid was used to generate different AAV based DNA constructs. For SpdCas9 cloning both EFGP and WPRE were removed using XbaI and XhoI and SpdCas9 and the modified smaller WPRE along with SV40 polyA signal (W3SL) were then cloned into this backbone using NEBuilder HiFi DNA Assembly. For expression of MS2-gRNA and hSyn-MCP-MSN from a single plasmid, both components were PCR amplified and cloned into an EGFP-removed hSyn-AAV-EGFP backbone using NEBuilder HiFi DNA Assembly. For All-In-One AAV backbone the M11 promoter[12] was used to drive SaCas9 gRNA expression. The SCP1[13] and the EFS promoters were used to drive the expression of NMS-SadCas9. The efficient, smaller synthetic WPRE and polyadenylation signal CW3SA[14] was utilized to maximize expression this size-limited context. Following cloning and sequence verification, 3 SaCas9 specific gRNAs targeting mouse *Agrp* gene were cloned into the all-in-one (AIO) vectors using Bbs1 restriction digestion. Following identification of the most efficacious gRNA (by transfecting into Neuro-2a cells), the SCP1 and EFS promoter driven SadCas9 based AIO plasmids were sequence verified by Plasmidsaurus. Sequence verified SpdCas9 and SCP1 and EFS promoter driven SadCas9 based AIO plasmids were sent to Charles River Laboratories for AAV8 production. Titers of different AAVs are included in source data.

**gRNA design and construction**

All protospacer sequences for SpCas9 systems were designed using the Custom Alt-R® CRISPR-Cas9 guide RNA design tool (IDT). All gRNA protospacers were then phosphorylated, annealed, and cloned into chimeric U6 promoter containing sgRNA cloning plasmid (Addgene #47108) and/or an MS2 loop containing plasmid backbone (Addgene #61424) digested with Bbs1 and treated with alkaline phosphatase (Thermo) using T4 DNA ligase (NEB). The SaCas9 gRNA expression plasmid (pIBH072) was a kind gift from Charles Gersbach and was digested with BbsI or BpiI (NEB or Thermo, respectively) and treated with alkaline phosphatase and then annealed protospacer sequences were cloned in using T4 DNA ligase (NEB). gRNAs were cloned into the pU6-Cj-sgRNA expression plasmid (Addgene #89753) by digesting the vector backbone with BsmBI or Esp3I (NEB or Thermo, respectively), and then treating the digested plasmid with alkaline phosphatase, annealing phosphorylated gRNAs, and then cloning annealed gRNAs into the backbone using T4 DNA ligase. MS2-stem loop containing plasmids for SaCas9 and CjCas9 were designed as gBlocks (IDT) with an MS2-stem loop incorporated into the tetraloop region for both respective gRNA tracr sequences. crRNA expression plasmids for the Type I *Eco* Cascade system were generated by annealing synthetic DNA ultramers (IDT) containing direct repeats (DRs) and cloning these ultramers into the BbsI and SacI-digested SpCas9 sgRNA cloning plasmid (Addgene #47108) using NEBuilder HiFi DNA Assembly. crRNA expression plasmids for *Pae* Type I Cascade system were

generated by annealing and then PCR-extending overlapping oligos (that also harbored a BsmBI or Esp3I cut site for facile crRNA array incorporation) into the sequentially BbsI (or BpiI) and SacI-digested SpCas9 sgRNA cloning plasmid (Addgene #47108) using NEBuilder HiFi DNA Assembly. crRNA expression plasmids for Cas12a systems were generated by annealing and then PCR-extending overlapping oligos (that also harbored a BsmBI or Esp3I cut site for facile crRNA array incorporation) into the sequentially BbsI (or BpiI) and SacI-digested SpCas9 sgRNA cloning plasmid (Addgene #47108) using NEBuilder HiFi DNA Assembly. All gRNA spacer sequences used in this study for SpdCas9, SadCas9, and CjdCas9 are listed in **Supplementary Tables 1, 2, and 3,** respectively.

**crRNA array cloning**
crRNA arrays for AsCas12a and Type I CRISPR systems were designed in fragments as overlapping ssDNA oligos (IDT) and 2-4 oligo pairs were annealed. Oligos were designed with an Esp3I cut site at 3' of the array for subsequent cloning steps. Equimolar amounts of oligos were mixed, phosphorylated, and annealed similar to the standardized gRNA/crRNA assembly protocol above. Phosphorylated and annealed arrays were then cloned into the respective Esp3I-digested and alkaline phosphatase treated crRNA cloning backbone (described above) using T4 DNA ligase (NEB). crRNA arrays were verified by Sanger sequencing. Correctly assembled 4-8 crRNA array expressing plasmids were then digested again with Esp3I and alkaline phosphatase treated to enable incorporation of subsequent arrays up to 20 crRNAs. All crRNA spacer sequences used in this study for AsdCas12a, *Eco*-Cascade, and *Pae*-Cascade are listed in **Supplementary Tables 4, 5, and 6,** respectively.

## Supplementary References


1. Zhao, B. et al. Inactivation of YAP oncoprotein by the Hippo pathway is involved in cell contact inhibition and tissue growth control. Genes Dev 21, 2747-2761 (2007).
2. Tanenbaum, M.E., Gilbert, L.A., Qi, L.S., Weissman, J.S. & Vale, R.D. A protein-tagging system for signal amplification in gene expression and fluorescence imaging. Cell 159, 635-646 (2014).
3. Konermann, S. et al. Genome-scale transcriptional activation by an engineered CRISPR-Cas9 complex. Nature 517, 583-588 (2015).
4. Katoh, Y. et al. Two domains of Nrf2 cooperatively bind CBP, a CREB binding protein, and synergistically activate transcription. Genes Cells 6, 857-868 (2001).
5. Piskacek, S. et al. Nine-amino-acid transactivation domain: establishment and prediction utilities. Genomics 89, 756-768 (2007).
6. Bromberg, J. & Darnell, J.E., Jr. The role of STATs in transcriptional control and their impact on cellular function. Oncogene 19, 2468-2473 (2000).
7. Zetsche, B. et al. Multiplex gene editing by CRISPR-Cpf1 using a single crRNA array. Nat Biotechnol 35, 31-34 (2017).
8. Campa, C.C., Weisbach, N.R., Santinha, A.J., Incarnato, D. & Platt, R.J. Multiplexed genome engineering by Cas12a and CRISPR arrays encoded on single transcripts. Nat Methods 16, 887-893 (2019).
9. Hilton, I.B. et al. Epigenome editing by a CRISPR-Cas9-based acetyltransferase activates genes from promoters and enhancers. Nat Biotechnol 33, 510-517 (2015).

10. Weltner, J. & Trokovic, R. Reprogramming of Fibroblasts to Human iPSCs by CRISPR Activators. Methods Mol Biol 2239, 175-198 (2021).
11. Weltner, J. et al. Human pluripotent reprogramming with CRISPR activators. Nat Commun 9, 2643 (2018).
12. Gao, Z. et al. Engineered miniature H1 promoters with dedicated RNA polymerase II or III activity. J Biol Chem 296, 100026 (2021).
13. Juven-Gershon, T., Cheng, S. & Kadonaga, J.T. Rational design of a super core promoter that enhances gene expression. Nat Methods 3, 917-922 (2006).
14. Choi, J.H. et al. Optimization of AAV expression cassettes to improve packaging capacity and transgene expression in neurons. Mol Brain 7, 17 (2014).

**Supplementary Figure 1**

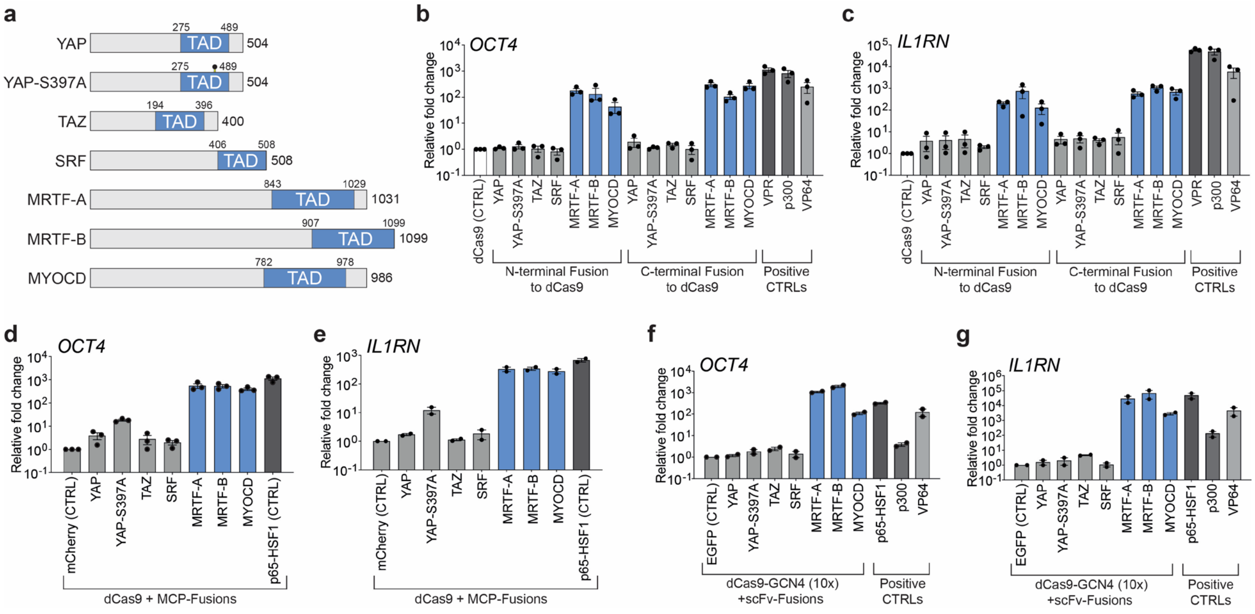


**Supplementary Fig. 1. Transactivation potency of serum responsive MTF TADs when recruited to human promoters via dCas9. a.** Schematics showing Hippo and SRF-MRTF family proteins; YAP, the hyperactive YAP mutant (YAP S397A), TAZ, SRF, MRTF-A, MRTF-B and MYOCD proteins. TADs for each respective MTF, along with amino acid (aa) coordinates, are shown in light blue. **b and c.** *OCT4* and *IL1RN* mRNA levels after the indicated TADs were fused to the N- or C-terminus of dCas9 targeted to each respective promoter using 4 pooled gRNAs. **d and e.** *OCT4* and *IL1RN* mRNA levels after the indicated TADs were fused to the MCP protein and recruited via 4 pooled MS2 modified gRNAs and dCas9. MCP fused to the bipartite p65-HSF was used as a positive control. **f and g.** *OCT4* and *IL1RN* mRNA levels after the indicated TADs were fused to scFv and recruited via dCas9 harboring 10xGCN4 C-terminal fusion protein (the SunTag system) along with 4 pooled standard gRNAs targeting each respective promoter. All samples were processed for QPCR analysis 72 hours post-transfection in HEK293T cells. Data are the result of 3 biological replicates for **panel b – d** and 2 biological replicates for **panels e – i**. See source data for more information. Data are presented as mean values +/- SEM.

**Supplementary Figure 2**

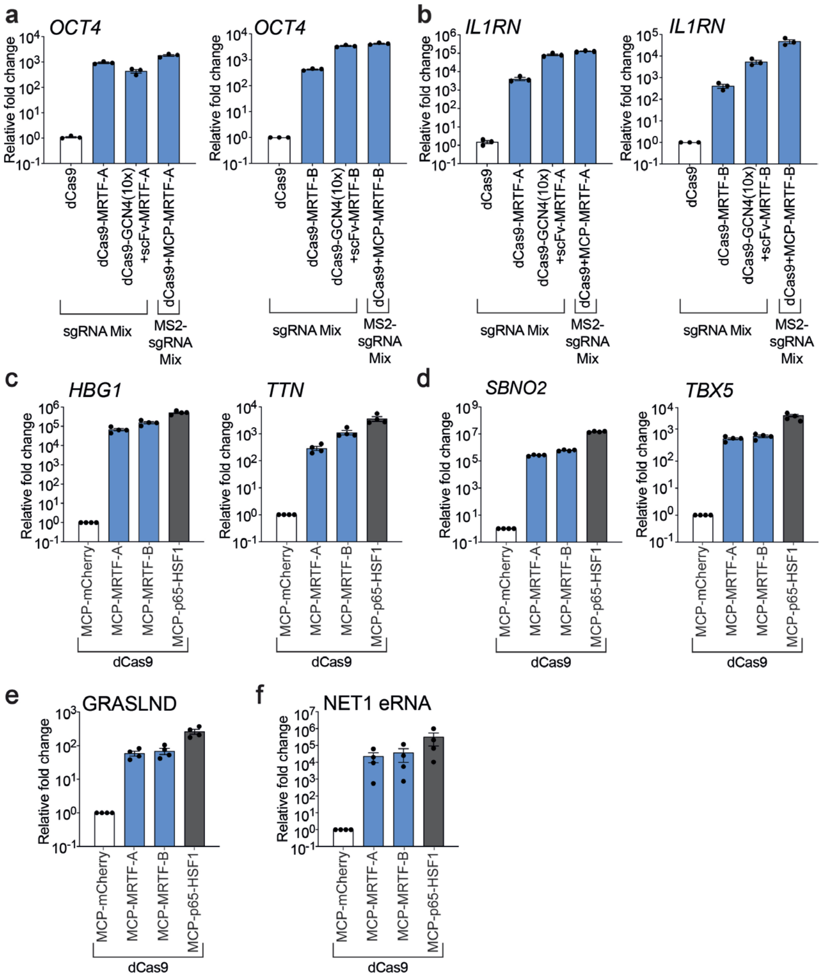


**Supplementary Fig. 2. Comparison and versatility of gene activation potential between TADs from MRTF-A and MRTF-B. a and b.** *OCT4* and *IL1RN* mRNA levels after the indicated TADs from MRTA-A (left panels) or MRTF-B (right panels) were recruited using the specified dCas9-based recruitment architecture (direct fusion, SunTag-based, or MCP-based) and 4 corresponding pooled gRNAs. **c and d.** mRNA levels for indicated loci after TADs from MRTF-A or MRTF-B were recruited via dCas9 and targeted to promoters using pools of MS2 modified gRNAs (**panel c**; *HBG1* and *TTN* promoters) or a single gRNA (**panel d**; *SBNO2* and *TBX5* promoters). **e and f**. GRASLND long noncoding RNA (**left**) or NET1 eRNA (**right**) levels after TADs from MRTF-A or MRTF-B were recruited via dCas9 and targeted to each respective locus using 4 and 2 pooled MS2 modified gRNAs, respectively. All samples were processed for QPCR analysis 72 hours post-transfection in HEK293T cells. Data are the result of 3 biological replicates for **panel a, and b** and 4 biological replicates for **panels c – f**. See source data for more information. Data are presented as mean values +/- SEM.

## Supplementary Figure 3

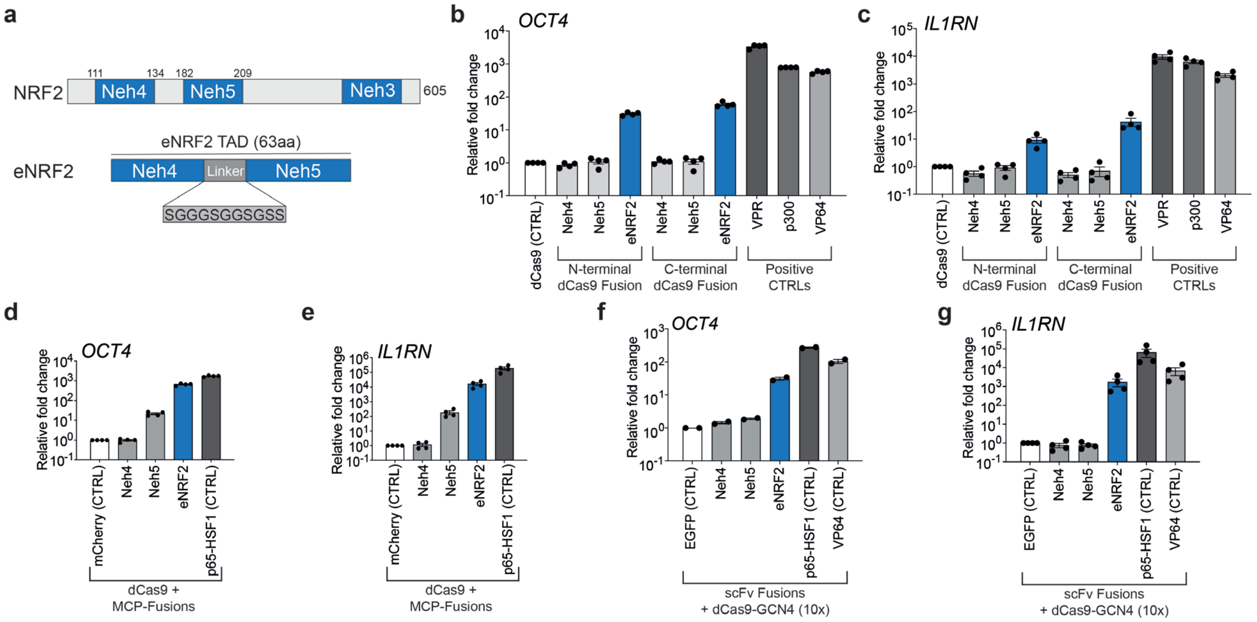


**Supplementary Fig. 3. Transactivation potency of oxidative stress responsive MTF TADs when recruited to human promoters via dCas9. a.** TADs from the NRF2 protein are depicted schematically. eNRF2 is a fusion between Neh4 and Neh5 TADs separated by an 11 amino acid (aa) extended glycine-serine linker. The length of NRF2 and aa coordinates of individual TADs (Neh4 and Neh5) are shown. **b and c.** *OCT4* and *IL1RN* mRNA levels after the indicated TADs were fused to the N- or C-terminus of dCas9 and targeted to each respective promoter using 4 pooled standard gRNAs. **d and e.** *OCT4* and *IL1RN* mRNA levels after the indicated TADs were fused to the MCP protein and recruited via 4 pooled MS2 stem-loop modified gRNAs and dCas9. MCP fused to the bipartite p65-HSF was used as a positive control. **f and g.** *OCT4* and *IL1RN* mRNA levels after the indicated TADs were fused to scFv and recruited via dCas9 harboring 10xGCN4 C-terminal fusion protein (the SunTag system) along with 4 pooled standard gRNAs targeting each respective promoter. All samples were processed for QPCR analysis 72 hours post-transfection in HEK293T cells. Data are the result of 4 biological replicates for **panel b – e, and g** and 2 biological replicates for **panels f**. See source data for more information. Data are presented as mean values +/- SEM.

## Supplementary Figure 4

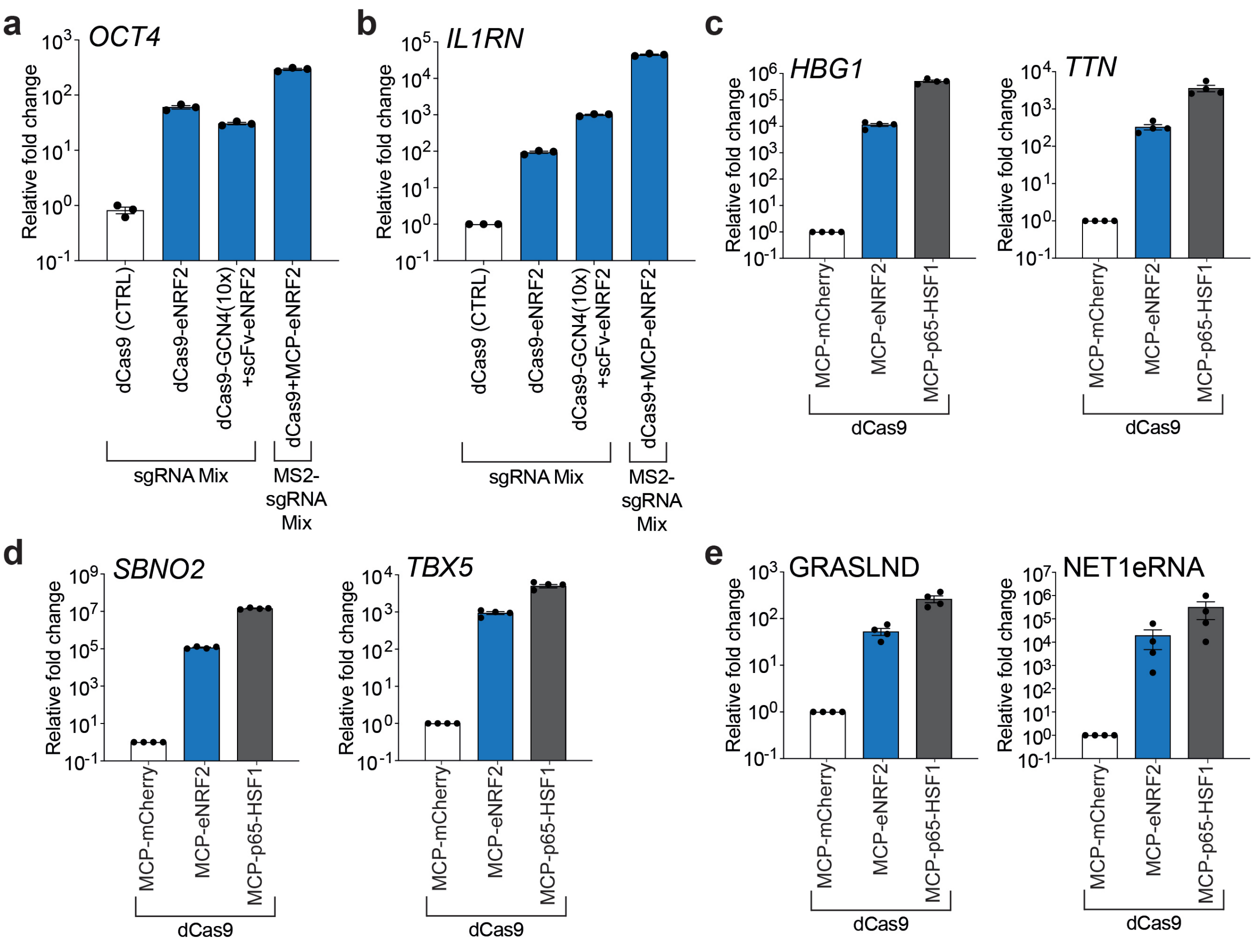


**Supplementary Fig. 4. Comparison and versatility of gene activation potential of the eNRF2 TAD. a and b.** *OCT4* and *IL1RN* mRNA levels after the eNRF2 TAD was recruited using the specified dCas9-based recruitment architecture (direct fusion, SunTag-based, or MCP-based) and 4 corresponding pooled gRNAs. **c and d.** mRNA levels for indicated loci after the eNRF2 TAD was recruited via dCas9 and targeted to promoters using pools of MS2 modified gRNAs (**panel c**; *HBG1* and *TTN* promoters) or a single gRNA (**panel d**; *SBNO2* and *TBX5* promoters). **e**. GRASLND long noncoding RNA (left) or NET1 eRNA (right) levels after the eNRF2 TAD was recruited via dCas9 and targeted to each indicated locus using 4 and 2 pooled MS2 modified gRNAs, respectively. All samples were processed for QPCR analysis 72 hours post-transfection in HEK293T cells. Data are the result of 3 biological replicates for **panel a, and b** and 4 biological replicates for **panels c – e**. See source data for more information. Data are presented as mean values +/- SEM.

## Supplementary Figure 5

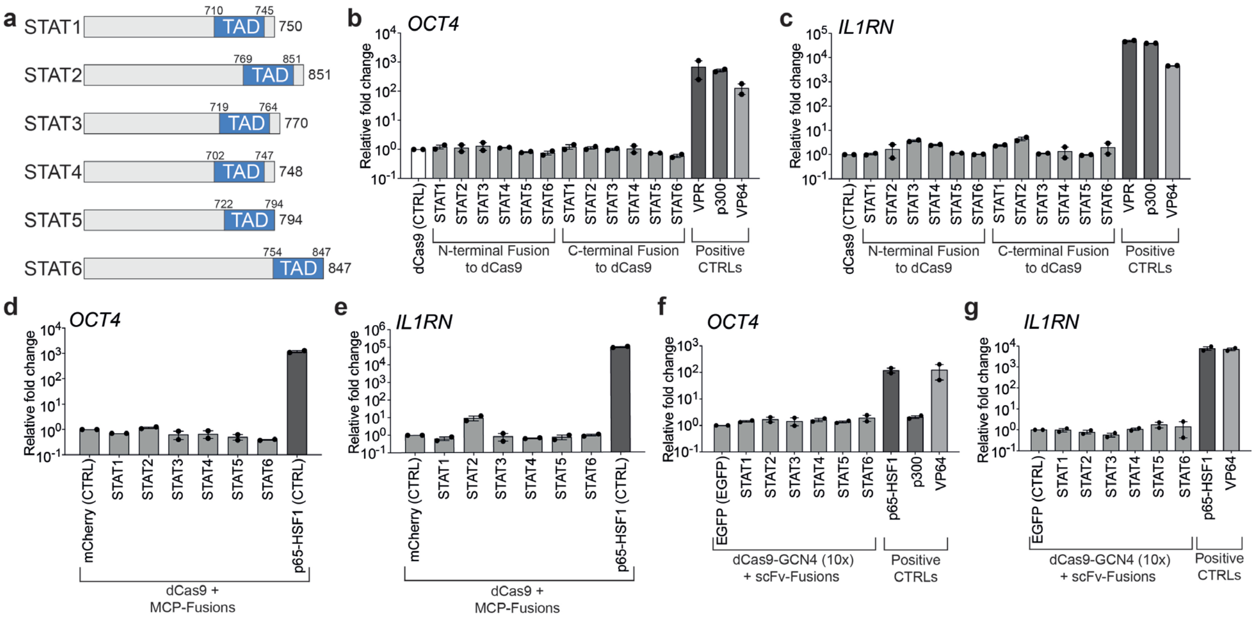


**Supplementary Fig. 5. Transactivation potency of cytokine responsive MTF TADs when recruited to human promoters via dCas9. a.** Schematics showing STAT family proteins STAT1, STAT2, STAT3, STAT4, STAT5 and STAT6. TADs for each respective protein are shown in light blue along with amino acid (aa) coordinates. **b and c.** *OCT4* and *IL1RN* mRNA levels after the indicated TADs were fused to the N- or C-terminus of dCas9 targeted to each respective promoter using 4 pooled standard gRNAs. **d and e.** *OCT4* and *IL1RN* mRNA levels after the indicated TADs were fused to the MCP protein and recruited via 4 pooled MS2 modified gRNAs and dCas9. MCP fused to the bipartite p65-HSF was used as a positive control. **f and g.** *OCT4* and *IL1RN* mRNA levels after the indicated TADs were fused to scFv and recruited via dCas9 harboring a 10xGCN4 C-terminal fusion protein (the SunTag system) along with 4 pooled standard gRNAs targeting each respective promoter. All samples were processed for QPCR analysis 72 hours post-transfection in HEK293T cells. Data are the result of 2 biological replicates for **panel b – g**. See source data for more information. Data are presented as mean values +/- SEM.

## Supplementary Figure 6

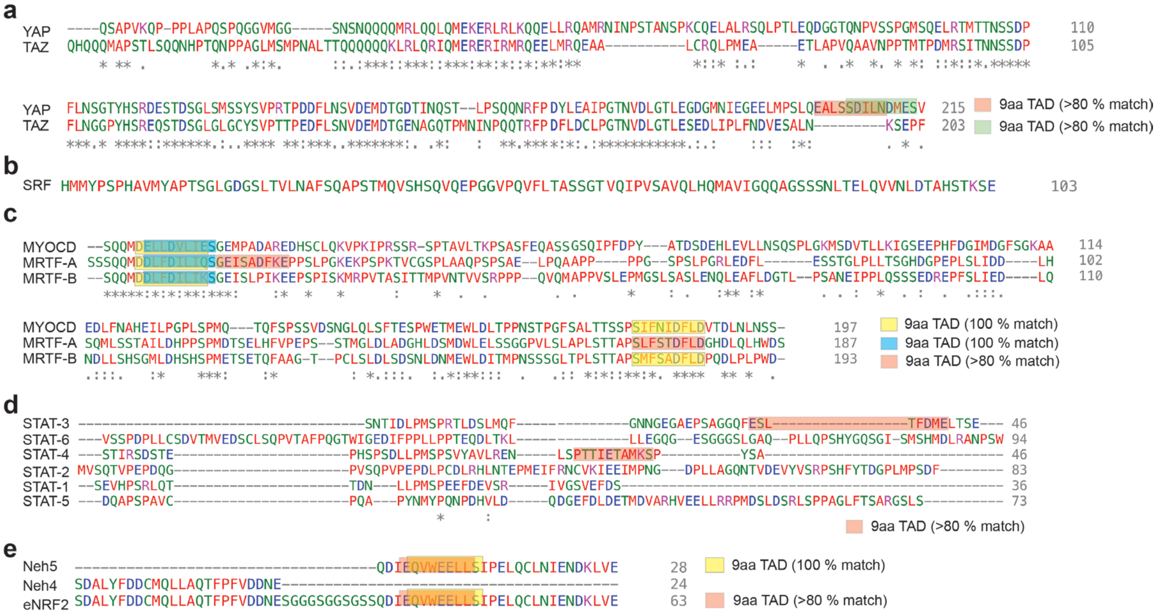


f

| TAD Domain | 9AA TAD (100% match) | 9AA TAD (>80% match) |
|---|---|---|
| YAP | 0 | 2 |
| TAZ | 0 | 0 |
| SRF | 0 | 0 |
| **MRTF-A** | **2** | **2** |
| **MRTF-B** | **3** | **0** |
| **MYOCD** | **3** | **0** |
| **Neh5** | **1** | **1** |
| Neh4 | 0 | 0 |
| **eNRF2** | **1** | **1** |
| STAT-1 | 0 | 0 |
| STAT-2 | 0 | 0 |
| STAT-3 | 0 | 1 |
| STAT-4 | 0 | 1 |
| STAT-5 | 0 | 0 |
| STAT-6 | 0 | 0 |

**Supplementary Fig. 6. Amino acid sequence analysis shows that potent mechanosensitive TAD domains contain single or multiple 9aa TAD segments with 100% matches to predictive software. a.** Clustal omega sequence alignment of TAD domains of two paralogous transcriptional activators YAP and TAZ. 9aa TADs with >80% match to algorithmic predictions (https://www.med.muni.cz/9aaTAD/) are either highlighted with light pink or light green. **b**. Amino acid sequence of the SRF TAD domain is shown. **c**. Clustal omega sequence alignment of TAD domains of the MYOCD, MRTF-A, and MRTF-B transcriptional activators are shown. 9aa TADs with 100% match to algorithmic predictions are either highlighted with light yellow or light blue and 9aa TADs with >80% to algorithmic predictions match are either highlighted with light pink or light green. **d**. Clustal omega sequence alignment of TAD domains from STAT-1 through STAT-6 are shown. 9aa TADs with >80% match to algorithmic predictions are highlighted with light pink. **e**. Amino acid sequence of Neh5, Neh4 and eNRF2 TADs are shown. 9aa TADs with 100% match to algorithmic predictions are highlighted with light yellow and 9aa TADs with >80% match to algorithmic predictions are highlighted with light pink. **f**. Table showing overall number of predicted 9aa TADs from all TAD domains used in this study.

## Supplementary Figure 7

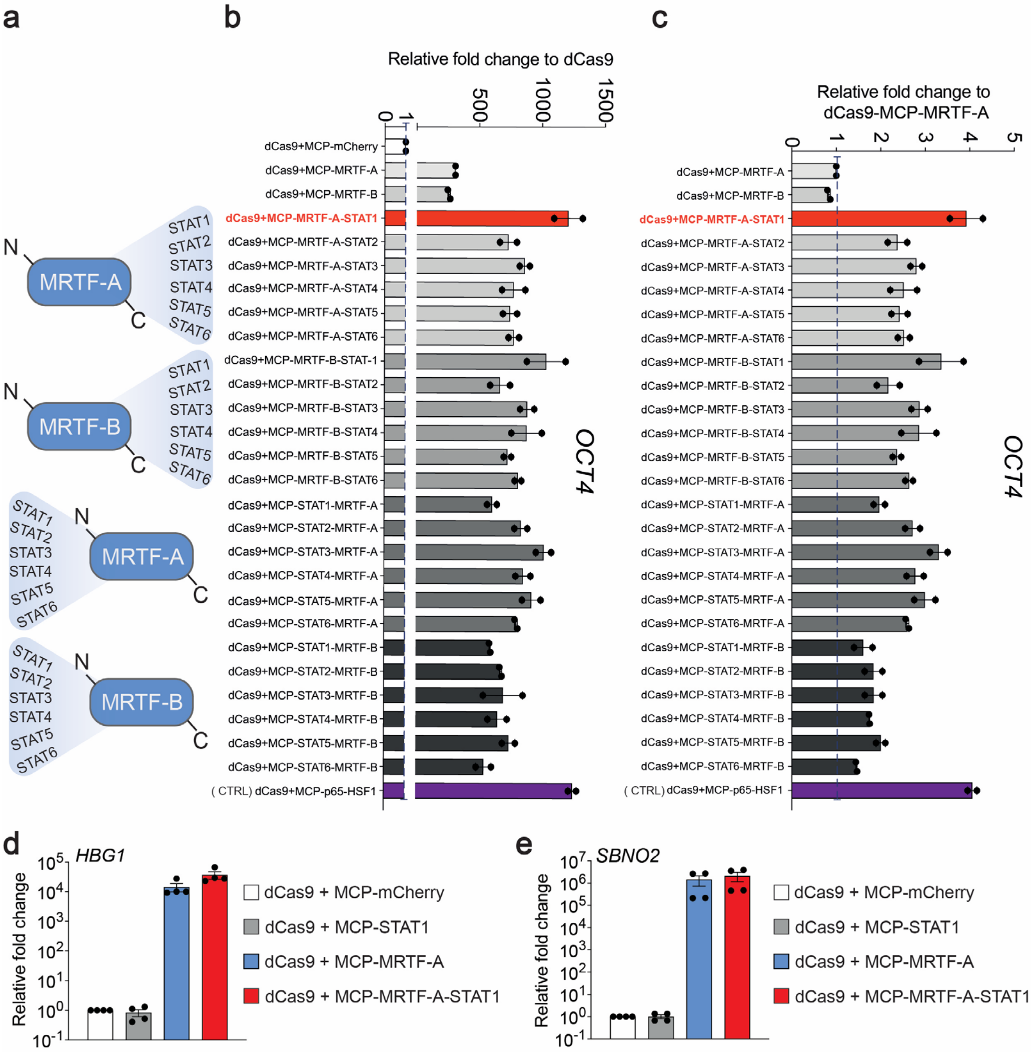


**Supplementary Fig. 7. Bipartite fusions between MRTF and STAT TADs enhance transactivation potential. a.** Schematic diagram showing the fusion of STAT1 through STAT6 TADs to the C- or N-terminus of MRTF-A or MRTF-B TADs. **b.** *OCT4* mRNA activation when targeted by indicated bipartite MRTF-STAT TAD fusions relative to dCas9 + MCP-mCherry. The dotted line indicates basal *OCT4* expression in dCas9 + MCP-mCherry transfected HEK293T cells. **c.** *OCT4* mRNA activation potential of indicated bipartite MRTF-STAT TAD fusions relative to dCas9 + MCP-MRTF-A. The dotted line indicates *OCT4* expression in dCas9 + MCP-MRTF-A transfected HEK293T cells. **d.** *HBG1* gene activation when dCas9 and indicated MCP fusions were targeted to the *HBG1* promoter using a pool of 4 MS2-modified gRNAs. **e.** *SBNO2* gene activation when dCas9 and indicated MCP fusions were targeted to the *SBNO2* promoter using a single MS2-modified gRNAs. All samples were processed for QPCR analysis 72 hours post-transfection. Data are the result of 2 biological replicates for **panel b, and c,** and 4 biological replicates for **panels d, and e**. See source data for more information. Data are presented as mean values +/- SEM.

## Supplementary Figure 8

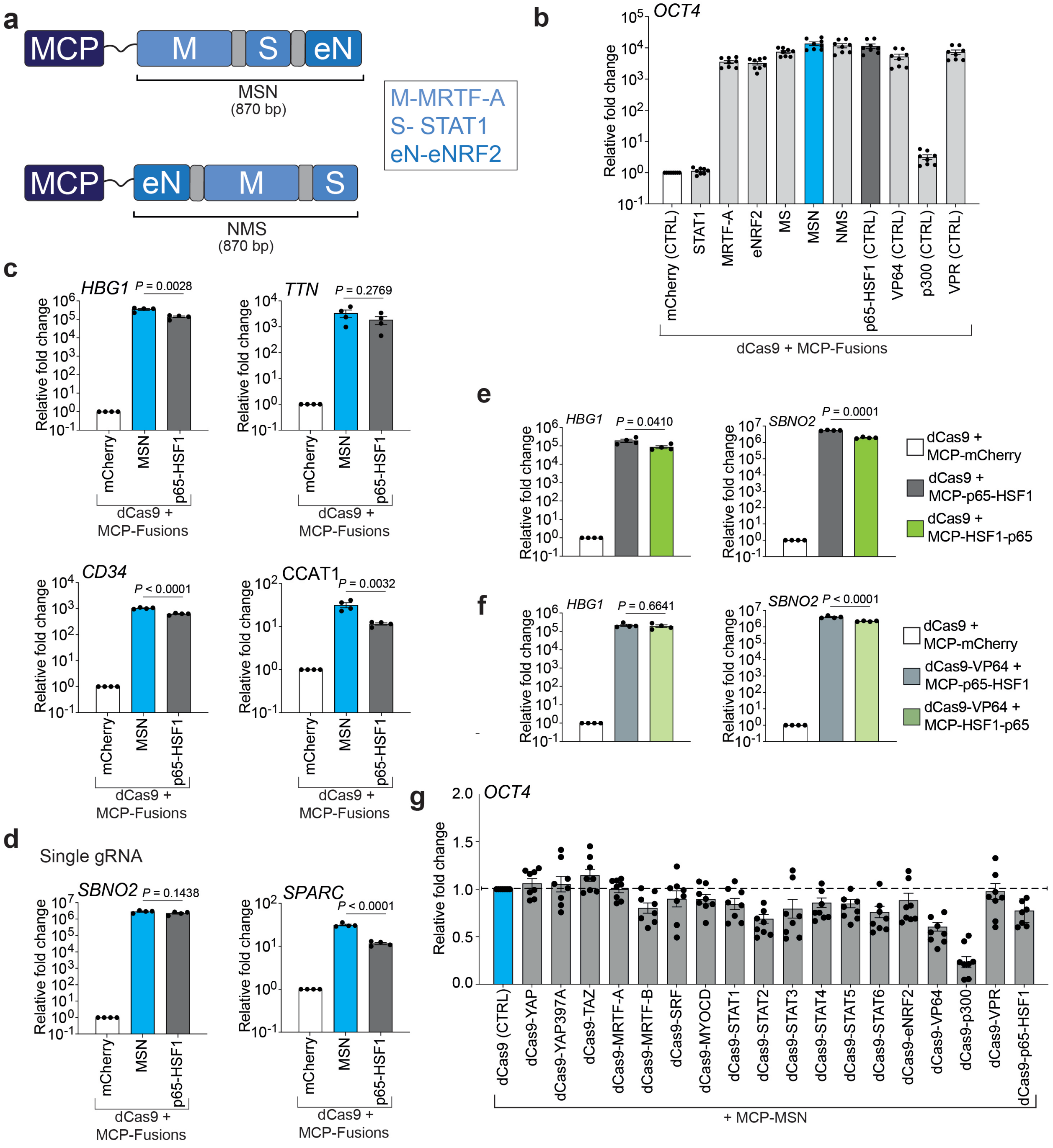


**Supplementary Fig. 8. Fusion of eNRF2 to the C- or N-terminus of MRTF-A-STAT1 (MS) fusions further enhances transactivation potency. a.** Schematic diagram showing tripartite MSN or NMS fusion proteins (base pair, bp sizes below) in conjunction with the MS2 binding protein; MCP. The eNRF2 TAD was either fused to the C-terminus or N-terminus of the bipartite MRTF-A-STAT1 TAD. **b.** *OCT4* mRNA levels after targeting with dCas9, 4 MS2-modified *OCT4* gRNAs, and the indicated MS2-recruited tripartite

TADs. **c.** Relative expression of *HBG1***,** *TTN***,** *CD34*, or CCAT1 after dCas9 + MCP-mCherry control, dCas9 + MCP-MSN, or dCas9 + MCP-p65-HSF1 systems were targeted to their respective promoters using pools of gRNAs in HEK293T cells. **d.** Relative expression of *SBNO2* or *SPARC* after dCas9 + MCP-mCherry control, dCas9 + MCP-MSN, or dCas9 + MCP-p65-HSF1 systems were targeted to their respective promoters using a single gRNA in HEK293T cells. **e.** Relative expression of *HBG1* or *SBNO2* mRNA levels after dCas9 + MCP-mCherry control, dCas9 + MCP-p65-HSF1, or dCas9 + MCP-HSF1-p65 systems were targeted to their respective promoters using pools of gRNAs or a single gRNA, respectively in HEK293T cells. **f.** Relative expression of *HBG1* or *SBNO2* mRNA levels after dCas9 + MCP-mCherry control, dCas9-VP64 + MCP-p65-HSF1, or dCas9-VP64 + MCP-HSF1-p65 systems were targeted to their respective promoters using pools of gRNAs or a single gRNA, respectively in HEK293T cells. **g.** *OCT4* mRNA levels after targeting with dCas9 or indicated direct dCas9 C-terminal TAD fusions, 4 MS2-modified gRNAs, and the MS2-recruited tripartite MCP-MSN TAD. *OCT4* activation levels are presented relative to the activation achieved by dCas9+MCP-MSN. All samples were processed for QPCR analysis 72 hours post-transfection. Data are the result of 8 biological replicates for **panels b,** 4 biological replicates for **c – f,** and 8 biological replicates for **panel g**. See source data for more information. Data are presented as mean values +/- SEM. *P* values determined using unpaired two-sided t-test.

**Supplementary Figure 9**

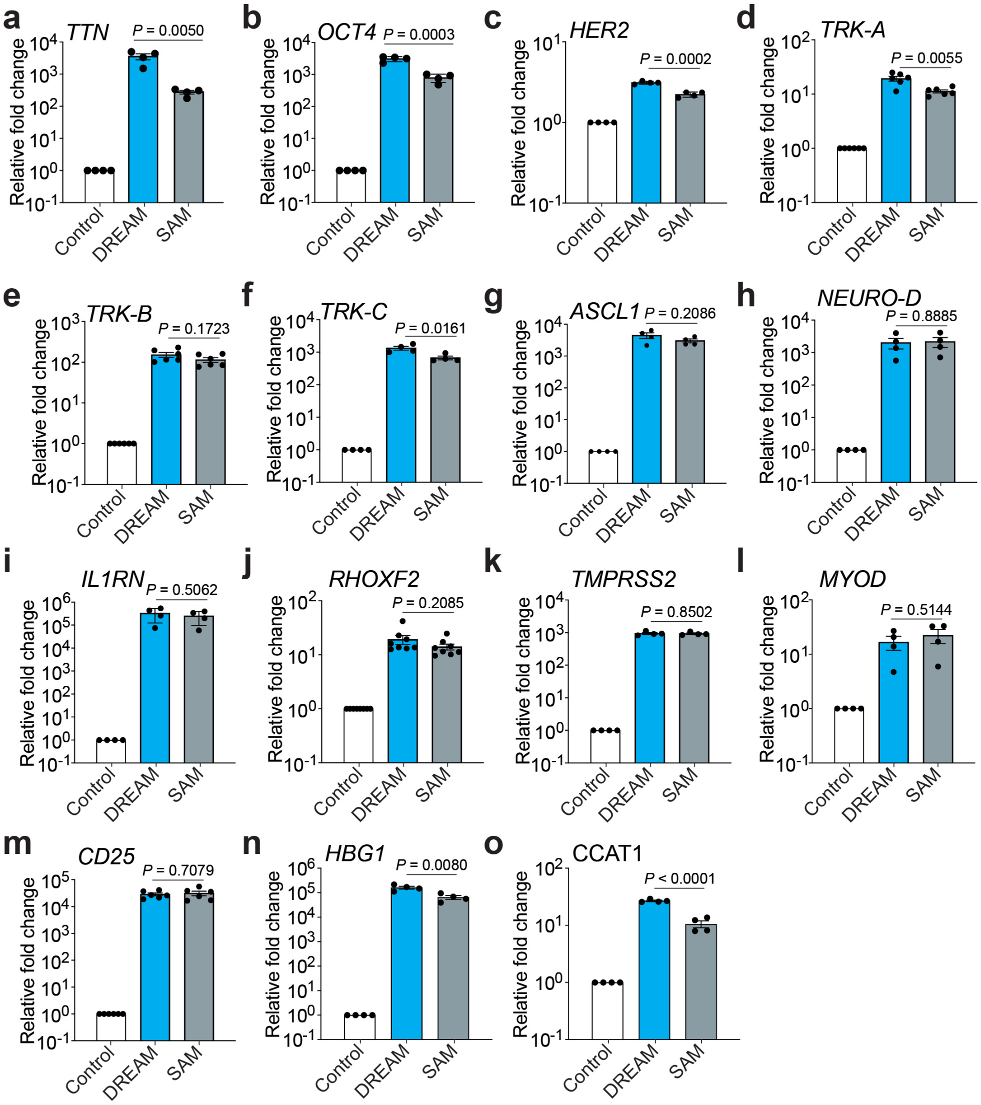


**Supplementary Fig. 9. CRISPR-DREAM mediated activation of coding genes in HEK293T cells using pooled gRNAs. a – m.** Relative expression levels of 13 different endogenous human genes after Control, DREAM, or SAM systems were targeted to their respective promoters using pools of gRNAs in HEK293T cells. **n and o.** Relative expression of *HBG1* or CCAT1 after equimolar amounts of control (187.5ng dCas9 + 187.05ng MCP-mCherry), DREAM (187.5ng dCas9 + 187.5ng MCP-MSN), or SAM (189.77ng dCas9-VP64 + 188.64ng MCP-p65-HSF1) systems were targeted to their respective promoters using pools of gRNAs, respectively in HEK293T cells. All samples were processed for QPCR analysis 72 hours post-transfection. Data are the result of 4 biological replicates for **panels a – c,** and **panels f – i, k, l, n and o.** Data are the result of 6 biological replicates for **panel d, e, and m** and 8 biological replicates for **panel j**. See source data for more information. Data are presented as mean values +/- SEM. *P* values determined using unpaired two-sided t-test.

## Supplementary Figure 10

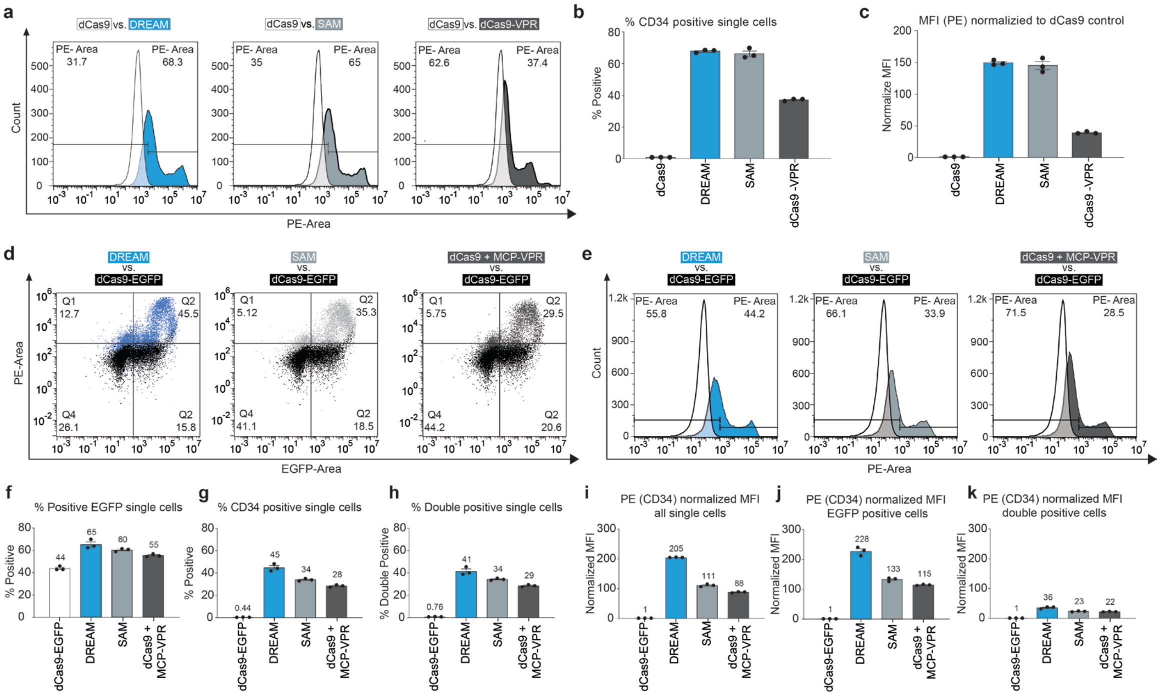


**Supplementary Fig. 10. Comparisons of CRISPR-DREAM, SAM, and dCas9-VPR induced CD34 expression in single HEK293T cells. a.** Histograms illustrating CD34-PE fluorescence after transfection of 4 *CD34*-targeting gRNAs and either DREAM (left in blue; gRNAs are MS2-modified), SAM (middle in light gray; gRNAs are MS2-modfied), dCas9-VPR (right in dark gray) or dCas9 (control in white). **b and c.** CD34 positivity and normalized mean fluorescence intensity (MFI) compared to dCas9 control, respectively following transfection of dCas9 (control), DREAM (dCas9 + MCP-MSN-STOP), SAM (dCas9 + MCP-p65-HSF1-STOP), or dCas9-VPR as in **panel a**. **d.** Histograms showing CD34 (via PE) and MCP-fusion (via EGFP) signal intensities after transfection of 4 MS2-modified *CD34*-targeting gRNAs and either DREAM (left in blue), SAM (middle in light gray), or dCas9 + MCP-VPR (right in dark gray) compared with dCas9-EGFP control (shown in black). **e.** Histograms illustrating CD34-PE fluorescence after transfection of 4 MS2-modified *CD34*-targeting gRNAs and either DREAM (left in blue), SAM (middle in light gray), dCas9 + MCP-VPR (right in dark gray) or dCas9-EGFP (control in white). **f and g.** Percent EGFP positive (**panel f**) and percent CD34 positive (**panel g**) single cell populations following transfection of 4 MS2-modified *CD34*-targeting gRNAs and either dCas9-EGFP (control), DREAM, SAM, or dCas9 + MCP-VPR. **h.** Percent CD34 and EGFP double positive HEK293T cells following transfection of 4 MS2-modified *CD34*-targeting gRNAs and either dCas9-EGFP (control), DREAM, SAM, or dCas9 + MCP-VPR. **i – k.** CD34 (via PE) MFI in single (**panel i**), EGFP positive (**panel j**), and double positive (**panel k**) cell populations following transfection of 4 MS2-modified *CD34*-targeting gRNAs dCas9-EGFP (control), DREAM, SAM, or dCas9 + MCP-VPR. Data are the result of 3 biological replicates for **panels b and c,** and **panels f – k.** Histogram shown in **panels a,d and e** are representative of 3 biological replicates**.** Data are presented as mean values +/- SEM.

## Supplementary Figure 11

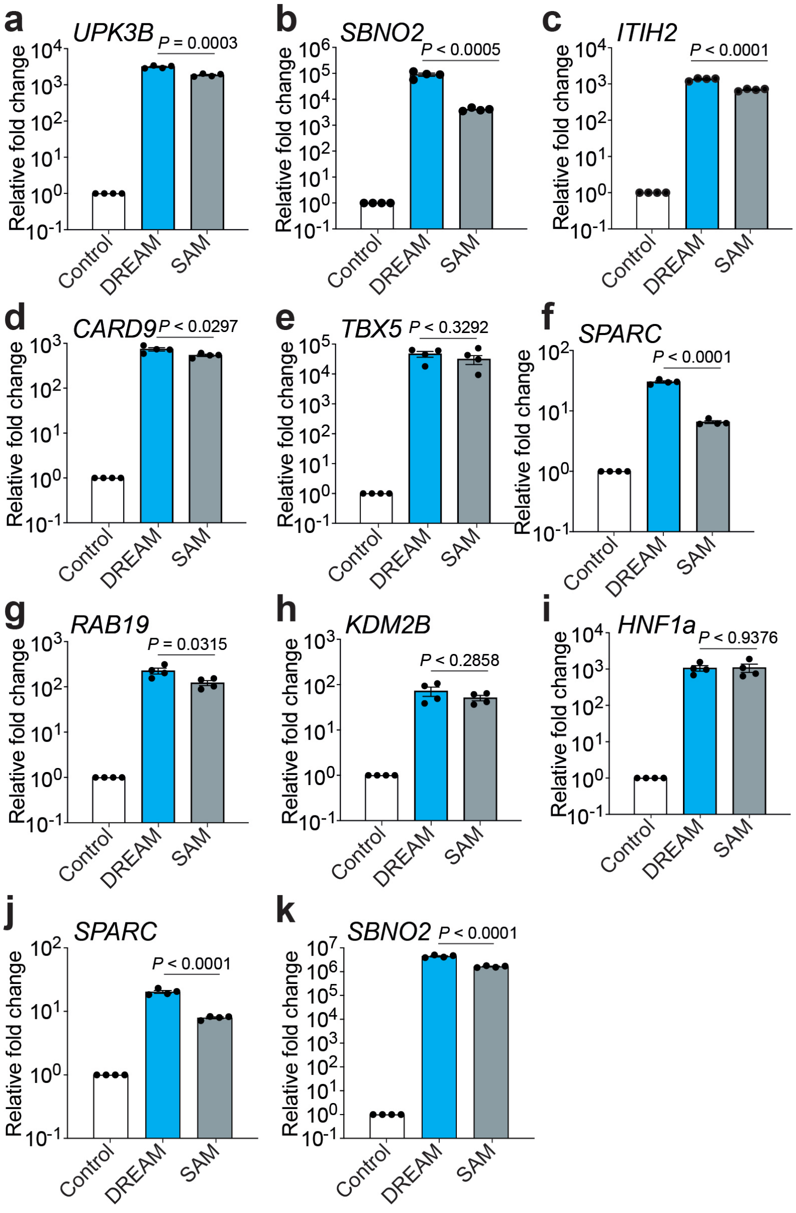


**Supplementary Fig. 11. CRISPR-DREAM mediated activation of coding genes in HEK293T cells using single gRNAs. a – i.** Relative expression levels of 9 different endogenous human genes 72 hours after Control, DREAM, or SAM systems were targeted to their respective promoters using a single gRNA in HEK293T cells**. j and k.** Relative expression of *SPARC* or *SBNO2* after equimolar amounts of control (187.5ng dCas9 + 187.05ng MCP-mCherry), DREAM (187.5ng dCas9 + 187.5ng MCP-MSN), or SAM (189.77ng dCas9-VP64 + 188.64ng MCP-p65-HSF1) systems were targeted to their respective promoters using a single gRNA, respectively in HEK293T cells. All samples were processed for QPCR analysis 72 hours post-transfection. Data are the result of 4 biological replicates for **panels a – k.** See source data for more information. Data are presented as mean values +/- SEM. *P* values determined using unpaired two-sided t-test.

## Supplementary Figure 12

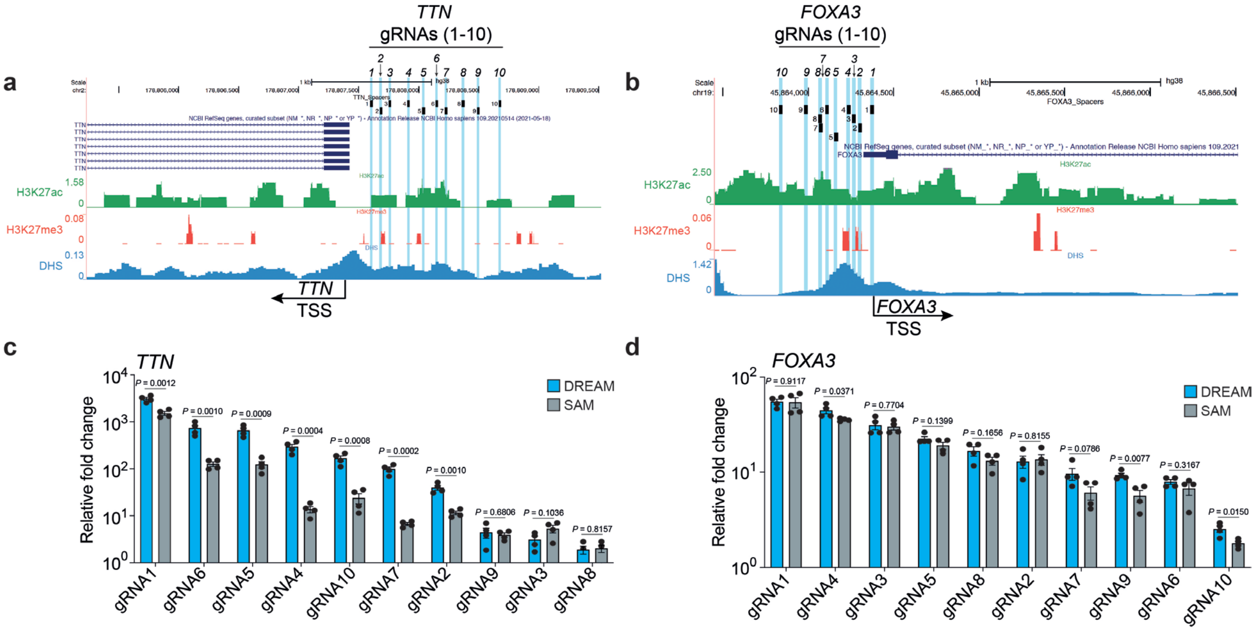


**Supplementary Fig. 12. Comparison of gene activation potency between DREAM and SAM systems spanning ~1kb regions upstream from human TSSs. a and b.** The genomic regions (hg38) encompassing the human *TTN* (**panel a**) and *FOXA3* (**panel b**) genes on chromosomes 2 and 19, respectively, are shown. Genes and isoforms are shown in dark blue; gRNA target regions are indicated by black lines, numbers, and light blue highlighting. H3K27ac (from GSE174866), H3K27me3 (from DRX013192), and DNase Hypersensitivity Sites (DHSs; from GSE32970) are shown in green, red, and blue, respectively. Transcription Start Sites (TSSs) for each gene indicated by black arrows. **c and d**. Comparison of transactivation potency between DREAM and SAM systems when targeted to indicated sites within the *TTN* or *FOXA3* promoters, respectively. 10 gRNAs were designed to tile across ~1kb upstream of respective promoter regions. Non-transfected HEK293T cells were used as control. All samples were processed for QPCR analysis 72 hours post-transfection. Data are the result of 4 biological replicates for **panels c and d.** See source data for more information. Data are presented as mean values +/- SEM. *P* values determined using multiple unpaired t-test.

## Supplementary Figure 13

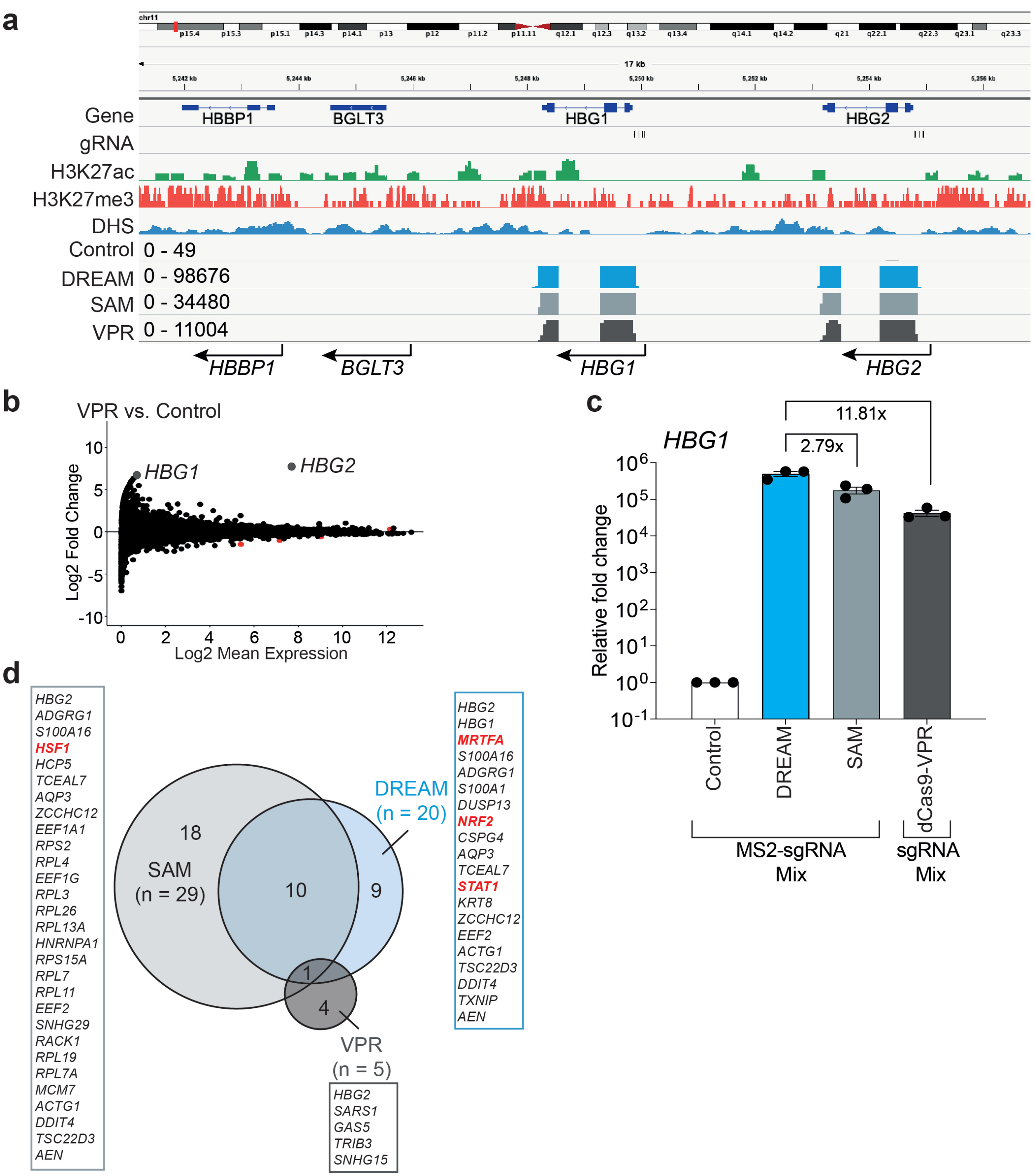


**Supplementary Fig. 13. CRISPR-DREAM mediated activation of *HBG1/HBG2* is specific, robust, and potent. a.** The genomic region encompassing the human *HBG1* and *HBG2* genes, along with two nearby genes *BGLT3* and *HBBP1* on chromosome 11 (hg38) is shown. Genes are shown in dark blue; gRNA target regions are indicated by black lines. H3K27ac (from GSE174866), H3K27me3 (from DRX013192), and DNase

Hypersensitivity Sites (DHSs; from GSE32970) are shown in green, red, and blue, respectively. **b.** An MA plot generated from DESeq2 analysis 72 hours after HEK293T cells were transiently co-transfected with dCas9-VPR and four *HBG1/HBG2* promoter targeting gRNAs. mRNAs corresponding to *HBG1* and *HBG2* isoforms (statistically significant differentially expressed with a fold change (FC) > 2 or < -2 and a false discovery rate (FDR) < 0.05) are shown in deep gray. Red dots indicate other statistically significant differentially expressed genes (FC > 2 or < -2, and FDR < 0.05). **c.** QPCR analysis showing *HBG1* expression after targeting with DREAM, SAM, or dCas9-VPR in HEK293T cells. dCas9 + MCP-mCherry was used as a control. All samples were processed for QPCR analysis 72 hours post-transfection in HEK293T cells and are the result of at least 3 biological replicates. Error bars; SEM. **d.** Venn diagram showing all statistically significant differentially regulated genes (FC > 2 or < -2, and FDR< 0.05) in HEK293T cells after the *HBG1/HBG2* promoters were targeted as above using the DREAM, SAM, or dCas9-VPR systems. Genes in bold red font are components of human TADs used in respective DREAM or SAM systems. Data are the result of 3 biological replicates for **panel c.** See source data for more information. Data are presented as mean values +/- SEM.

## Supplementary Figure 14

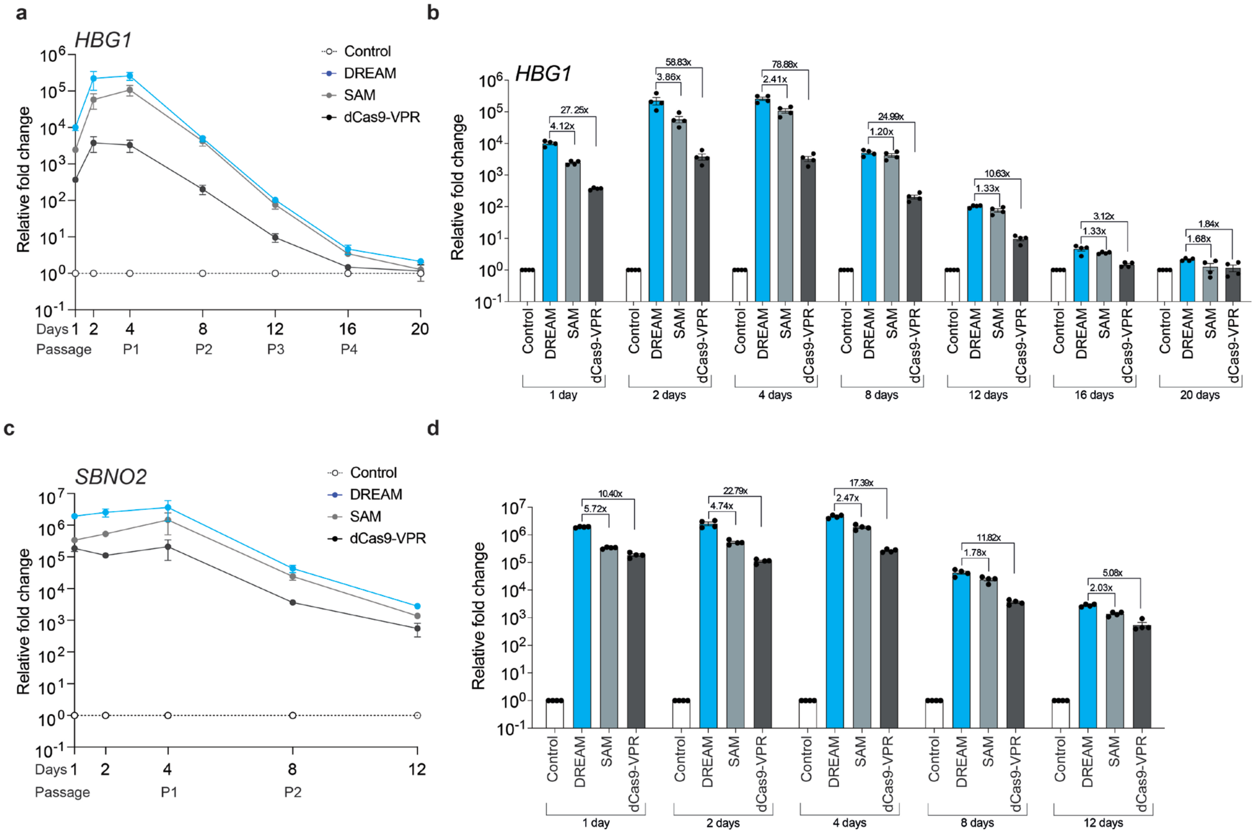


**Supplementary Fig. 14. Comparisons of functional durability between the DREAM, SAM, and dCas9-VPR systems in HEK293T cells. a and b.** *HBG1* mRNA expression is shown over 20 days post-transfection of 4 *HBG1* gRNAs and either DREAM (MS2-modified gRNAs), SAM (MS2-modified gRNAs), or dCas9-VPR compared with the dCas9 + MCP-mCherry control (MS2-modified gRNAs). ~15% of cells were passaged into a new 24 well plate at days 4, 8, 12, and 16 post-transfection. **c and d.** *SBNO2* mRNA expression is shown over 12 days post-transfection of 1 *SBNO2* gRNA and either DREAM (MS2-modified gRNA), SAM (MS2-modified gRNA), or dCas9-VPR compared with the dCas9 + MCP-mCherry control (MS2-modified gRNA). ~15% of cells were passaged into a new 24 well plate at days 4 and 8 post-transfection. Data are the result of 4 biological replicates for **panels a – d.** See source data for more information. Data are presented as mean values +/- SEM.

## Supplementary Figure 15

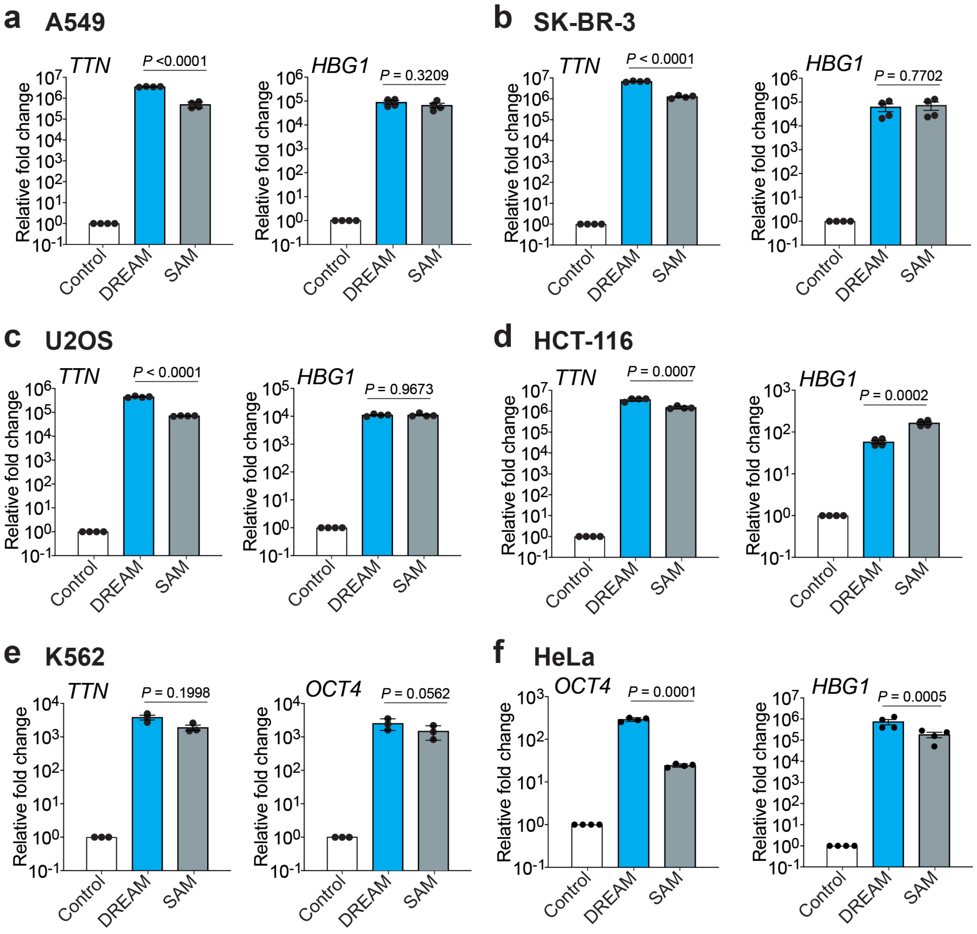


**Supplementary Fig. 15. CRISPR-DREAM is robust across diverse human cancer cell lines. a – f.** Transactivation potencies of DREAM and SAM systems are shown when targeted to indicated human promoters in a lung adenocarcinoma cell line (A549; **panel a**), a breast cancer cell line (SK-BR-3; **panel b**), a bone osteosarcoma epithelial cell line (U2OS **panel c**), a colorectal carcinoma cell line (HCT-116; **panel d**), a myelogenous leukemia cell line (K562; **panel e**), and a cervical cancer cell line (HeLa; **panel f**). All samples were processed for QPCR analysis 72 hours post-transfection. Data are the result of 4 biological replicates for **panels a – d, and f,** and 3 biological replicates for **panel e.** See source data for more information. Data are presented as mean values +/- SEM. *P* values determined using unpaired two-sided t-test.

**Supplementary Figure 16**

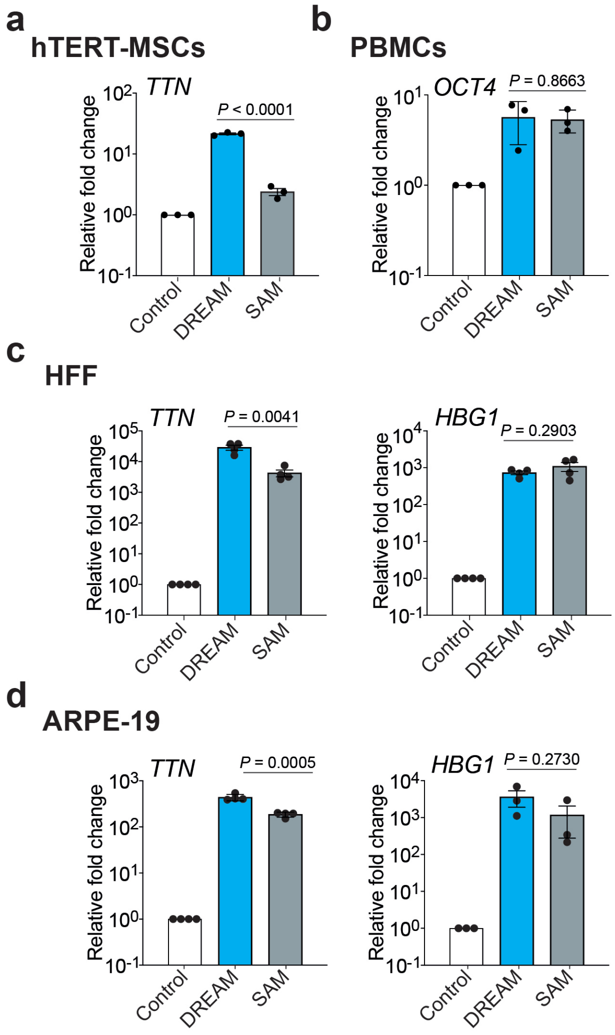


**Supplementary Fig. 16. CRISPR-DREAM is robust across diverse karyotypically normal human cells. a. a – c.** Transactivation potencies of DREAM and SAM systems are shown when targeted to indicated human promoters in hTERT-MSCs (**panel a**), PBMCs (**panel b**), Human Foreskin Fibroblasts (HFF; **panel c**), or retinal pigmented epithelial cells (ARPE-19; **panel d**). All samples were processed for QPCR analysis 72 hours post-transfection. Data are the result of 3 biological replicates for **panel a and b,** 4 biological replicates for **panel c,** and 3 – 4 biological replicates for **panel d.** See source data for more information. Data are presented as mean values +/- SEM. *P* values determined using unpaired two-sided t-test.

**Supplementary Figure 17**

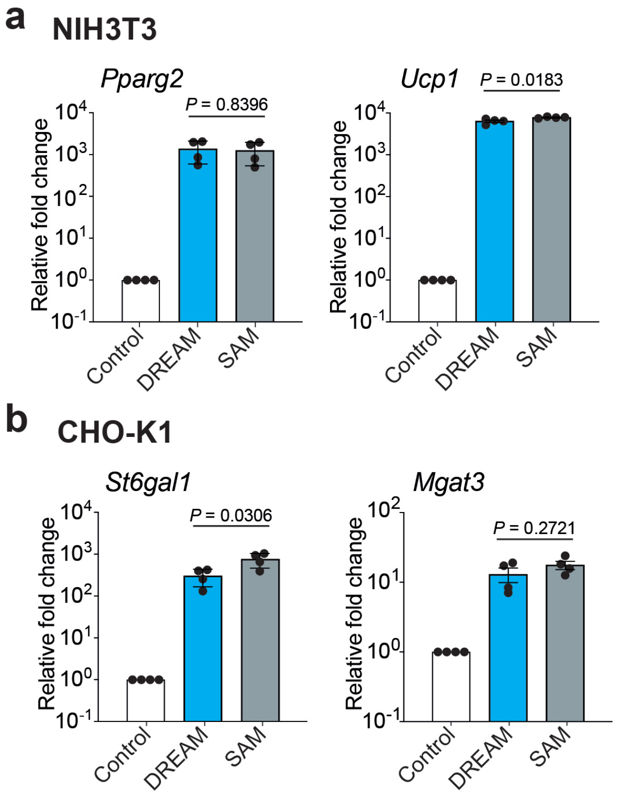


**Supplementary Fig. 17. CRISPR-DREAM is robust in rodent cells. a and b.** Transactivation potencies of DREAM and SAM systems are shown when targeted to indicated human promoters in murine NIH3T3 cells (**panel a**) or Chinese hamster ovary (CHO-K1) cells (**panel b**). All samples were processed for QPCR analysis 72 hours post-transfection. Data are the result of 4 biological replicates for **panels a and b.** See source data for more information. Data are presented as mean values +/- SEM. *P* values determined using unpaired two-sided t-test.

**Supplementary Figure 18**

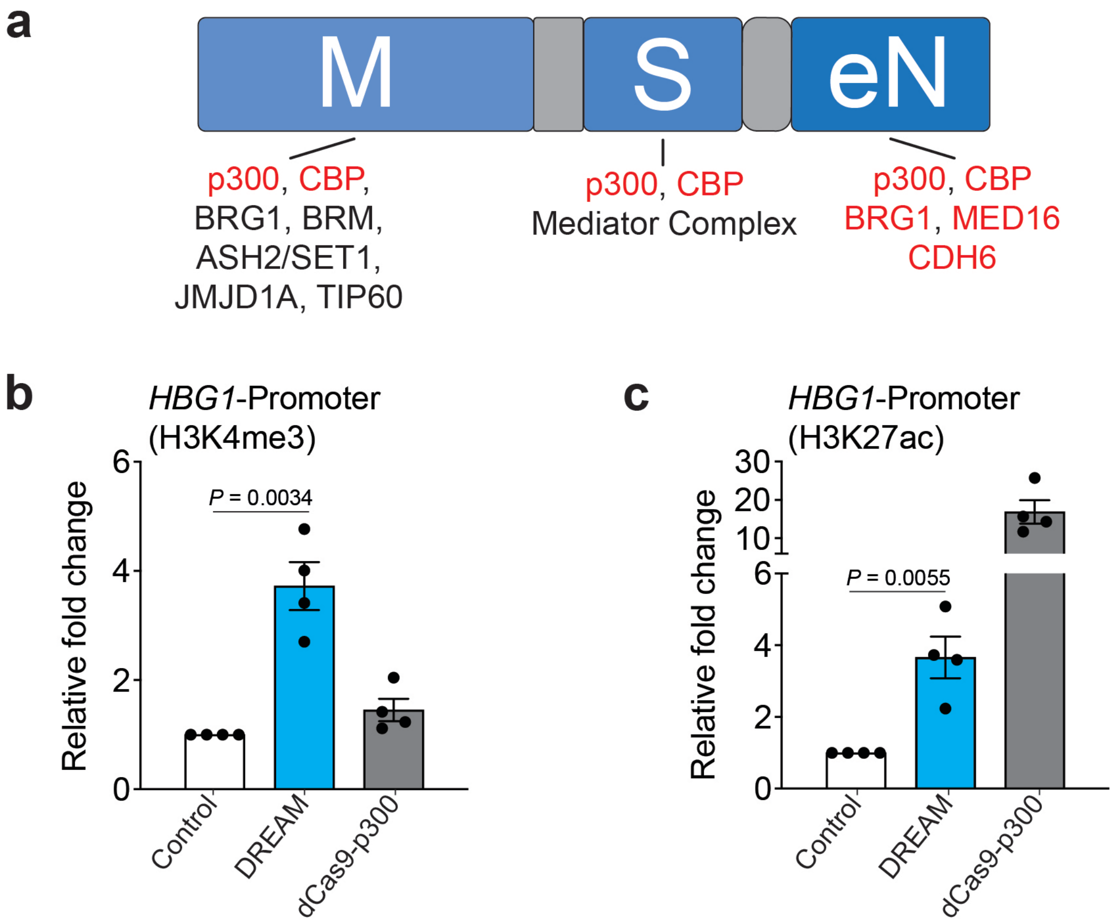


**Supplementary Fig. 18. DREAM deposits epigenetic marks related to transcriptional activation at a targeted promoter. a.** Schematic showing previously described interactions among different epigenetic modifiers/chromatin remodelers and MRTF-A, STAT1, and NRF2. Epigenetic modifiers/chromatin remodelers highlighted in red have been shown to interact directly with the TAD regions of MRTF-A, STAT1 and NRF2 (or eNRF2). Epigenetic modifiers/chromatin remodelers highlighted in black have been observed to interact with MRTF-A, STAT1, and NRF2, but interaction contacts are incompletely characterized to the best of our knowledge. **b and c.** Relative level of H3K4me3 and H3K27ac enrichment after control (dCas9 + MCP-mCherry), DREAM, or dCas9-p300 were targeted to the *HBG1* promoter using pools of 4 MS2-modified gRNA (control or DREAM) or pools of 4 standard gRNAs (dCas9-p300) in HEK293T cells. All samples were processed for CUT&RUN analysis 72 hours post-transfection. Data are the result of 4 biological replicates for **panels b and c.** See source data for more information. Data are presented as mean values +/- SEM. *P* values determined using unpaired two-sided t-test

**Supplementary Figure 19**

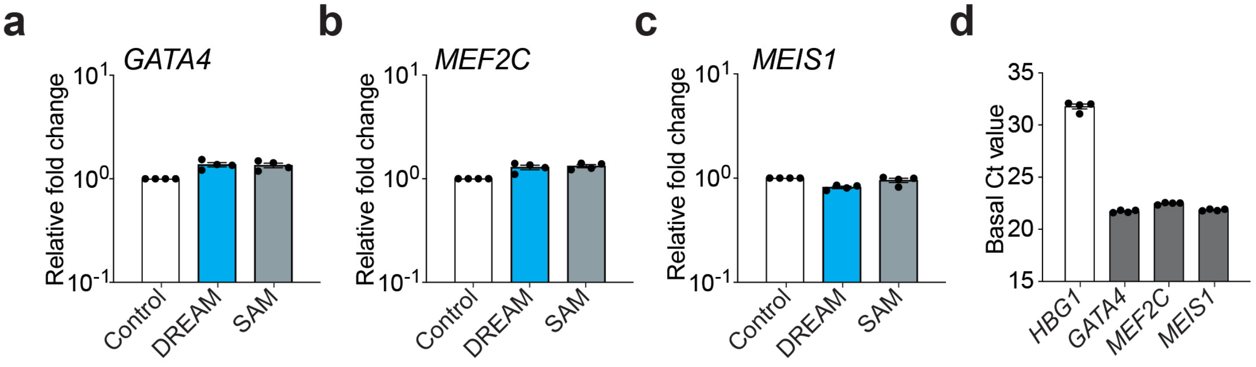


**Supplementary Fig. 19. DREAM and SAM systems fail to activate cardiomyocyte reprogramming genes in HEK293T cells. a – c.** Relative expression of *GATA4, MEF2C and MEIS1* after dCas9 control, DREAM, or SAM systems were targeted to their respective promoters using a single MS2-modifed gRNAs in HEK293T cells. **d.** Relative basal expression of *HBG1* (a relatively lowly expressed gene), *GATA4*, *MEF2C*, and *MEIS1* (relatively highly expressed genes) in HEK293T cells. Raw cycle threshold (Ct) values are shown as a proxy for expression levels. All samples were processed for QPCR analysis 72 hours post-transfection. Data are the result of 4 biological replicates for **panels a – d.** See source data for more information. Data are presented as mean values +/- SEM.

## Supplementary Figure 20

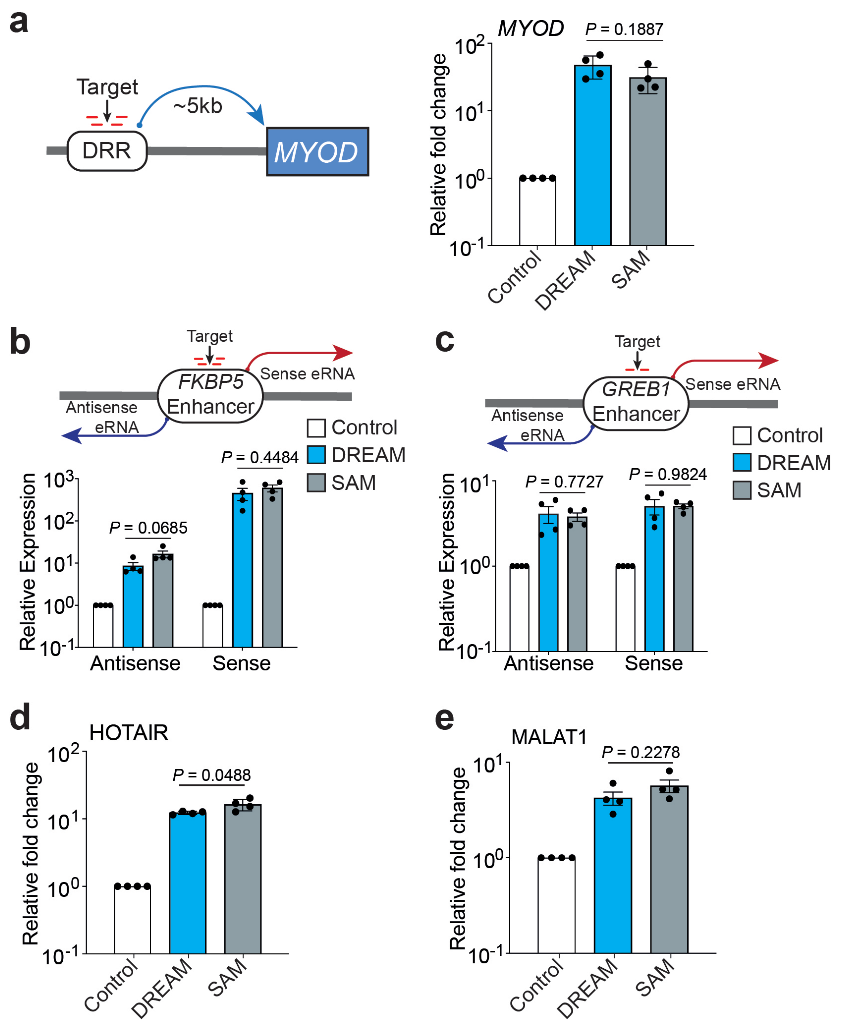


**Supplementary Fig. 20. CRISPR-DREAM activates gene expression when targeted to enhancers, activates eRNAs, and activates lncRNAs in human cells. a.** *MYOD* mRNA levels after DREAM or SAM systems were targeted to the *MYOD* distal regulatory region (DRR) using 4 MS2-modified gRNAs. **b and c**. eRNA levels induced after DREAM or SAM systems were targeted to the *FKBP5* (**panel b**) or *GREB1* (**panel c**) enhancer in HEK293T cells. **d and e**. lncRNA levels induced after DREAM or SAM systems were targeted to the HOTAIR (**panel d**) or MALAT1 (**panel e**) lncRNA promoters in HEK293T cells. All samples were processed for QPCR analysis 72 hours post-transfection and are the result of at least 4 biological replicates. See source data for more information. Data are the result of 4 biological replicates for **panels b – e.** See source data for more information. Data are presented as mean values +/- SEM. *P* values determined using unpaired two-sided t-test.

## Supplementary Figure 21

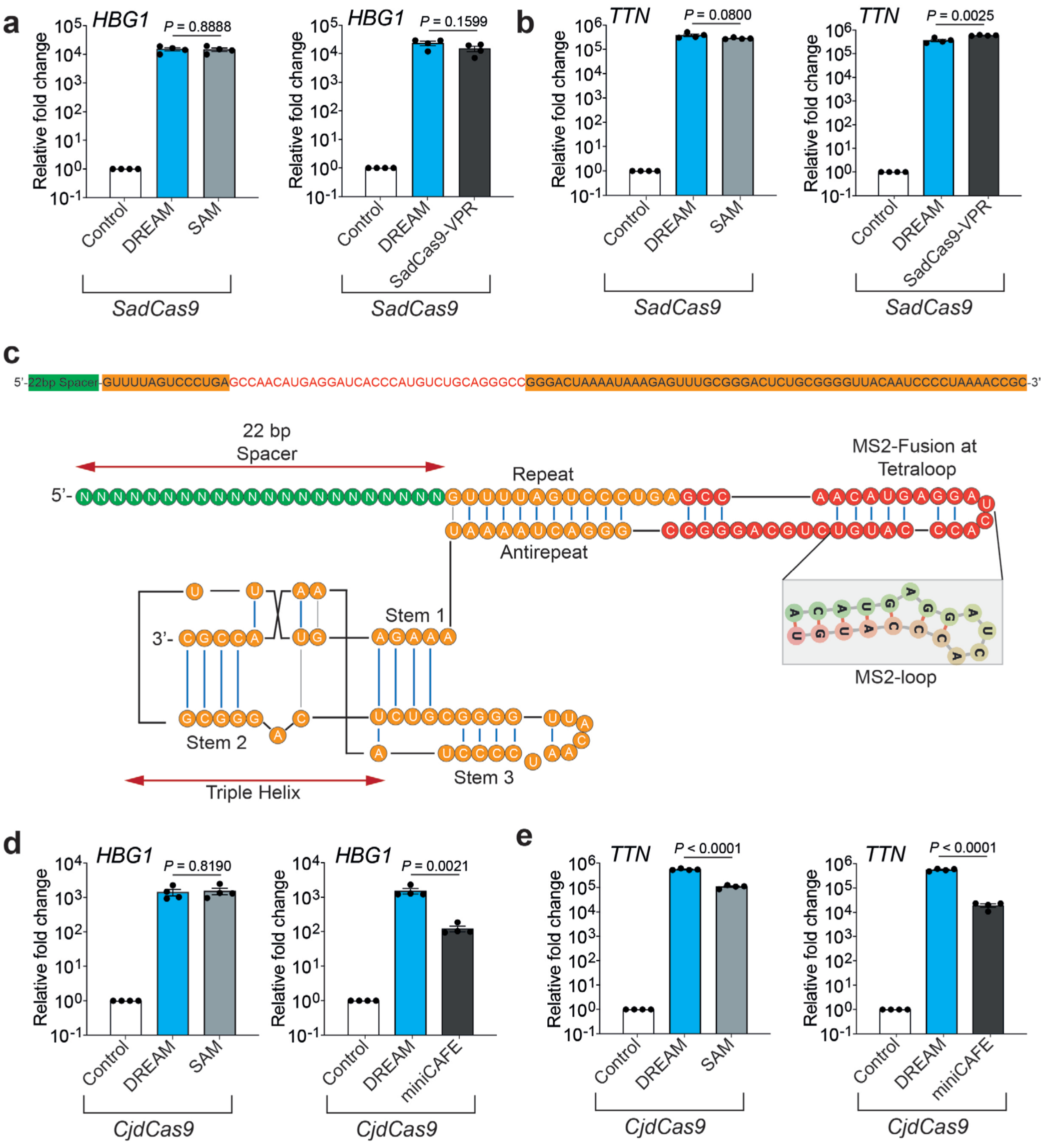


**Supplementary Fig. 21. Orthogonal CRISPR-DREAM systems are potent in HeLa cells. a and b.** SadCas9-DREAM mediated transactivation when targeted to the promoters of 2 different endogenous genes (*HBG1*; **panel a**, and *TTN;* **panel b**) in comparison to SadCas9-SAM or SadCas9-VPR systems. **c.** RNA sequence of the MS2 loop containing gRNA designed for CjdCas9 and schematics of CjCas9 gRNA with MS2 loop (magnified in inset) incorporated within the tetraloop region. **d and e.** CjdCas9-DREAM mediated transactivation when targeted to the promoters of 2 different endogenous genes (*HBG1*; **panel d**, and *TTN;* **panel e**) in comparison to CjdCas9-SAM or MiniCAFE systems. All samples were process for QPCR analysis 72 hours post-transfection. Data are the result of 4 biological replicates for **panels a, b, d,** and **e.** See source data for more information. Data are presented as mean values +/- SEM. *P* values determined using unpaired two-sided t-test.

## Supplementary Figure 22

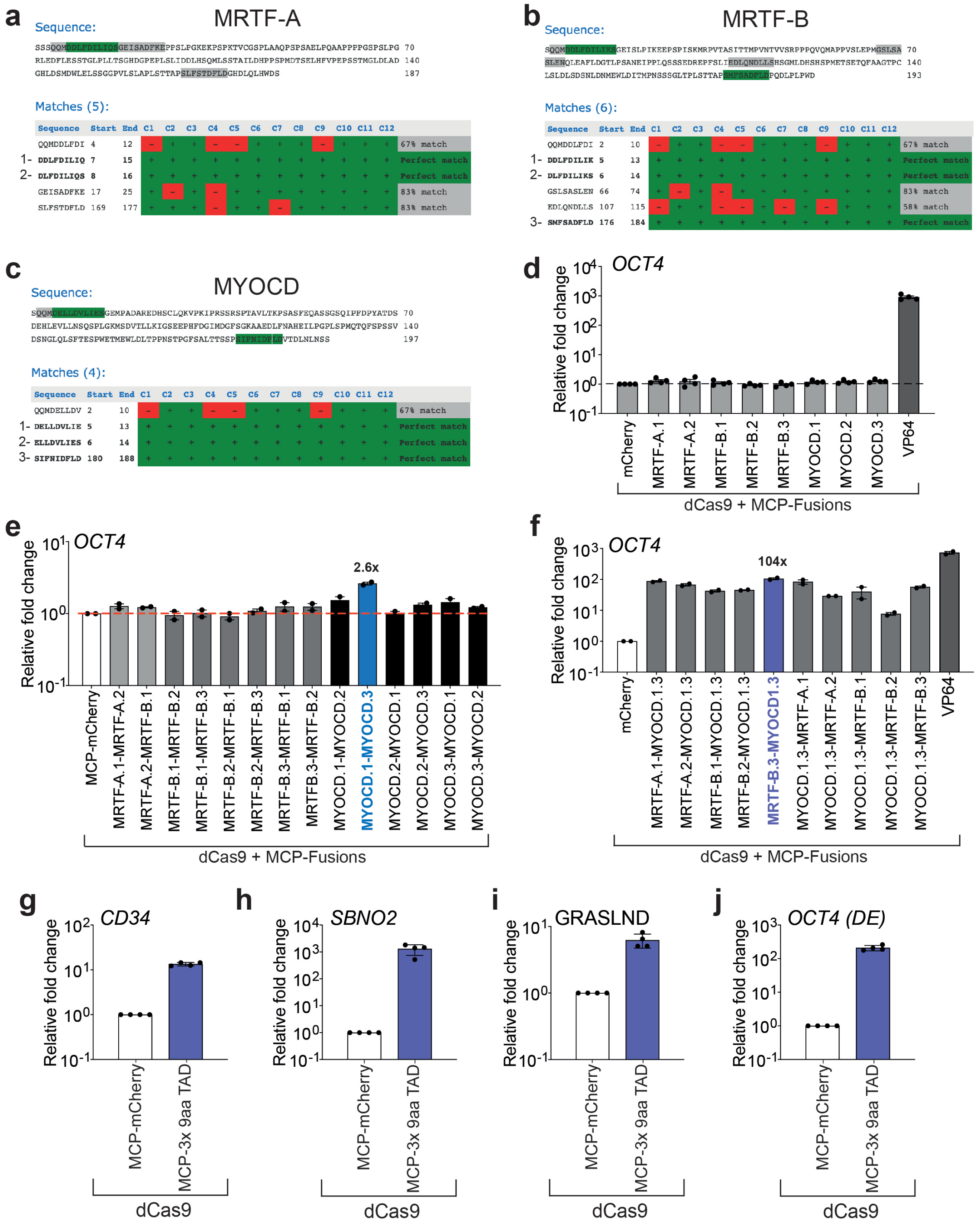


**Supplementary Fig. 22. Prediction, construction, and validation of transactivation potential among different 9aa TADs. a – c.** different 9aa TADs from MRTF-A (**panel a**), MRTF-B (**panel b**) or MYOCD (**panel c**) were predicted using the Nine Amino Acids Transactivation Domain 9aaTAD Prediction Tool (https://www.med.muni.cz/9aaTAD/). We selected 9aa TADs that showed 100% matches to database predictions. **d.** *OCT4*

gene activation when indicated 9aa TADs were fused to MCP and then recruited to *OCT4* promoter using dCas9 and a pool of 4 MS2-modified gRNAs. **e.** *OCT4* gene activation when indicated bipartite TADs, built using heterotypic 1x 9aa TADs, were fused to MCP and recruited to the *OCT4* promoter using dCas9 and a pool of 4 MS2-modified gRNAs. The blue bar (MCP-MYOCD.1-MYOCD.3) showed the highest gene activation among all 2x 9aa TADs and was selected for generating heterotypic 3x 9aa TADs. **f.** *OCT4* gene activation when indicated tripartite TADs, built using heterotypic 2x 9aa TADs, were fused to MCP and recruited to the *OCT4* promoter using dCas9 and a pool of 4 MS2-modified gRNAs. The purple bar (MCP-MRTF-B.3-MYOCD.1-MYOCD.3) showed the highest gene activation among all 3x 9aa TADs and was selected for further analysis. **g – h.** Relative gene activation when the selected 3x 9aa TAD (MCP-MRTF-B.3-MYOCD.1-MYOCD.3) was fused to MCP and recruited to either the *CD34* (**panel g**) or *SBNO2* (**panel h**) promoter using dCas9 and a pool of 4 MS2-modified gRNAs or a single MS2-modified gRNA, respectively. **i-j.** Levels of GRASLND lncRNA (**panel i**) or *OCT4* mRNA (**panel j**) after the selected 3x 9aa was recruited via dCas9 and targeted to either the *GRASLND* promoter or OCT distal enhancer (*OCT4*-DE), respectively, using pools of 4 MS2-modifed gRNAs. All samples were processed for QPCR analysis 72 hours post-transfection in HEK293T cells. Data are the result of 4 biological replicates for **panels d, and g – j,** and 2 biological replicates for **panels e and f.** See source data for more information. Data are presented as mean values +/- SEM.

**Supplementary Figure 23**

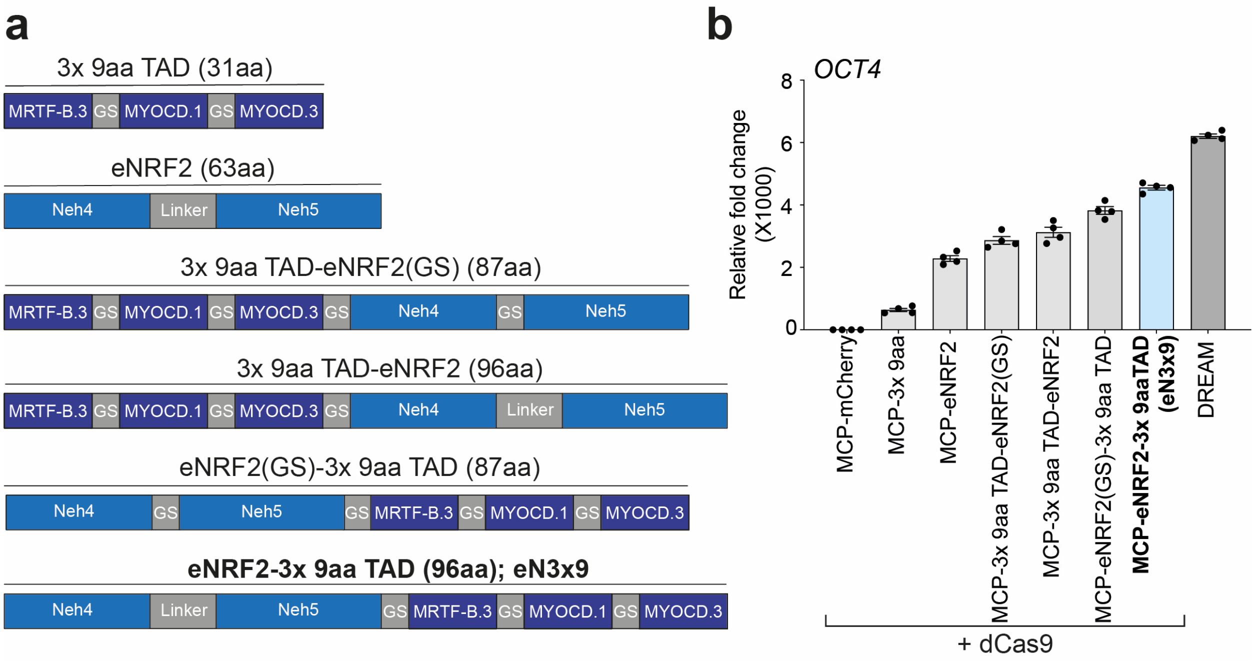


**Supplementary Fig. 23. Construction and validation of the eN3x9 TAD domain for the mini-DREAM system. a.** The 3x 9aa TADs derived from MRTF-B or MYOCD are depicted schematically. eNRF2 and different combinations of fusion architectures between indicated 3x 9aa TADs with eNRF2 fusions are also shown. 3x 9aa TADs were either cloned to the C- or N-terminal of eNRF2 and separated by either single glycine-serine linker (GS) or a 11 amino acid extended glycine-serine linker (Linker; see **Supplementary Fig. 3a**). Respective aa sizes of each fusion proteins are also shown. **b.** transcriptional activation of *OCT4* after 3x 9aa TAD, eNRF2, or indicated TAD fusions were recruited to the *OCT4* promoter via dCas9 and 4 pooled of MS2 modified gRNAs. The CRISPR-DREAM system is shown for comparison. All samples were processed for QPCR analysis 72 hours post-transfection in HEK293T cells. Data are the result of 4 biological replicates for **panel b.** See source data for more information. Data are presented as mean values +/- SEM.

## Supplementary Figure 24

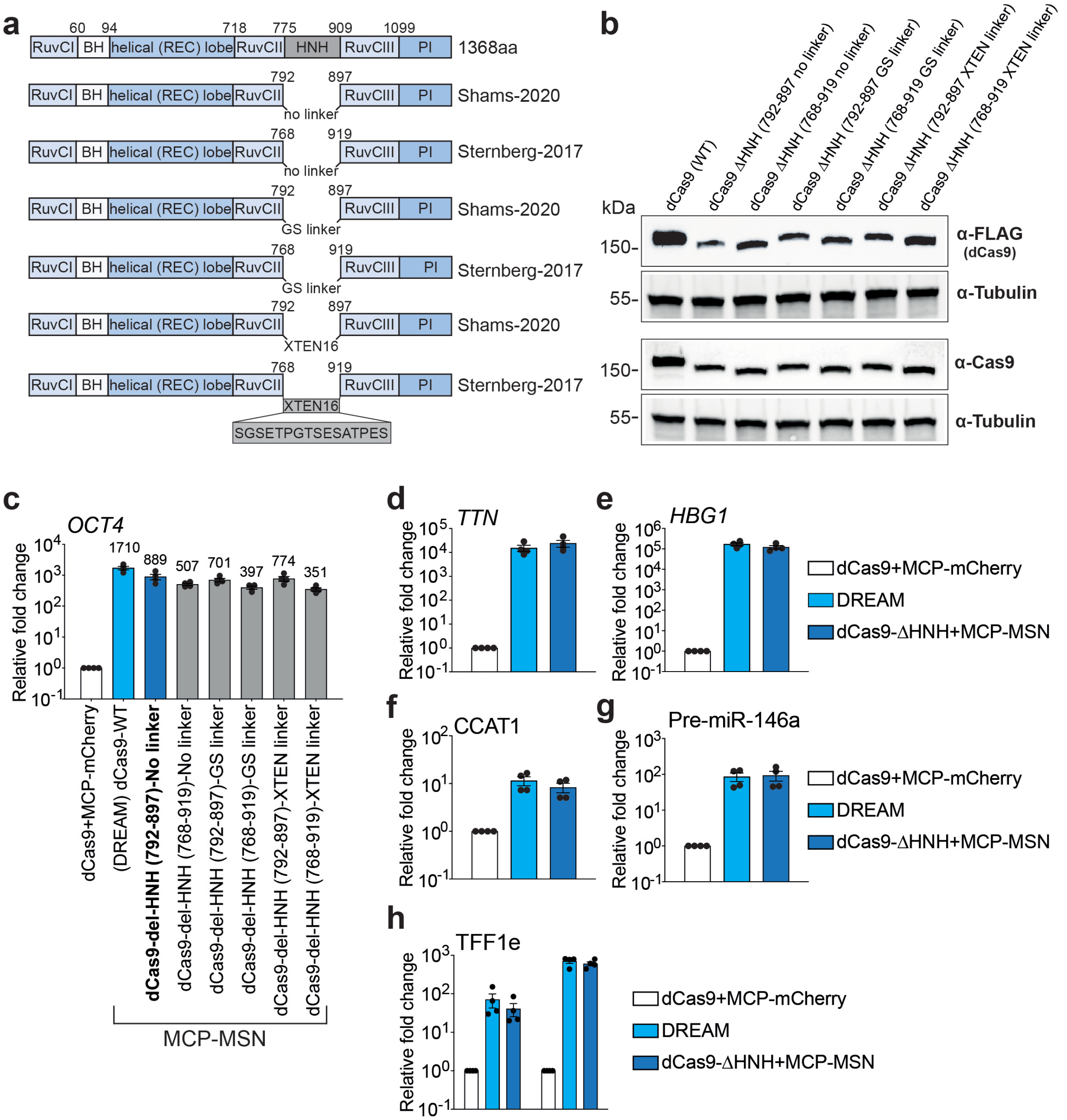


**Supplementary Fig. 24. Design, construction, and validation of HNH domain-deleted dCas9 variants for the mini-DREAM system. a.** Different HNH domain-deleted SpdCas9 variants, along with wildtype dCas9, are schematically depicted. Two different HNH domain-deleted SpCas9 variants (amino acid, aa 792-897, or aa 768-919 deleted, respectively) were selected for analysis and reconstructed using either no linker, a single glycine-serine linker, or an XTEN16 linker separating dCas9 protein segments. **b.** All HHN domain-deleted dCas9 variants were expressed in HEK293T cells and Western blotting was performed 72 hours post-transfection in HEK293T cells using either anti-FLAG or anti-Cas9 antibodies (Tubulin was used as a loading control). **c.** *OCT4* transactivation when MCP-MSN was recruited to the *OCT4* promoter using different indicated HNH domain-deleted dCas9 variants and a pool of 4 MS2-modified gRNAs. **d – h.** Comparison

of transactivation potential between CRISPR-DREAM and selected HNH domain-deleted dCas9 (with no linker) + MCP-MSN at indicated endogenous loci. All samples were processed for QPCR analysis 72 hours post-transfection in HEK293T cells. Data are the result of 4 biological replicates for **panels c – h**. Western blot data shown in **panel b** was from one independent experiment. See source data for more information. Data are presented as mean values +/- SEM.

## Supplementary Figure 25

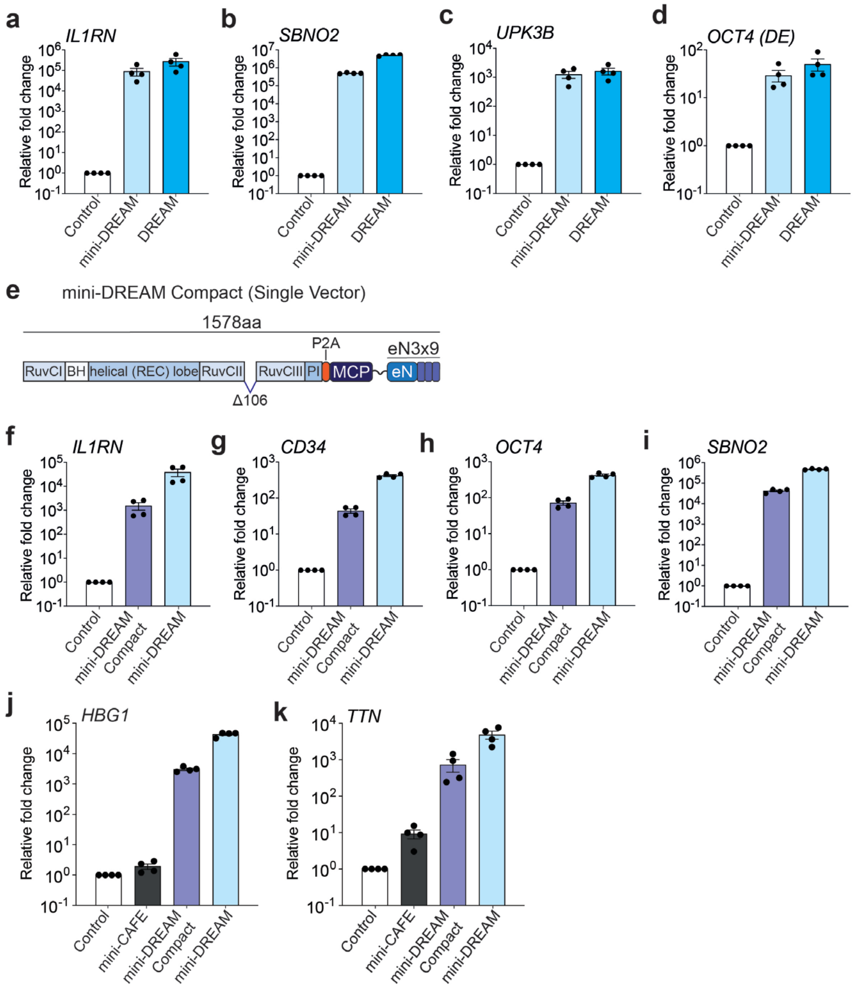


**Supplementary Fig. 25. mini-DREAM and mini-DREAM Compact systems display robust transactivation potencies in HEK293T cells. a – d.** Transactivation potencies of mini-DREAM and CRISPR-DREAM systems are shown when targeted to the *IL1RN* promoter (**panel a**) using pooled MS2-modified gRNAs, the *SBNO2* (**panel b**) or *UPK3B* promoters (**panel c**) using a single MS2-modified gRNA, respectively, and the *OCT4* distal enhancer (DE; **panel d**) using pooled MS2-modified gRNAs. **e.** The mini-DREAM Compact system is schematically depicted, P2A; self-cleaving peptide. **f – i.** Transactivation potencies of mini-DREAM and mini-DREAM Compact systems are shown when targeted to the *IL1RN* (**panel f**), *CD34* (**panel g**) or *OCT4* (**panel h**) respectively, using pooled MS2-modified gRNAs, or to the *SBNO2* promoter using a single MS2-modified gRNA (**panel i**). **j – k.** *HBG1* (left) or *TTN* (right) gene activation when either the CjdCas9 based mini-CAFE, SpdCas9 based mini-DREAM Compact or mini-DREAM system was targeted to each corresponding promoter using a pool of 4 MS2-modified gRNAs, respectively. All samples were processed for QPCR 72 hours post-transfection. Data are the result of 4 biological replicates for **panels a – d,** and **f – k.** See source data for more information. Data are presented as mean values +/- SEM.

## Supplementary Figure 26

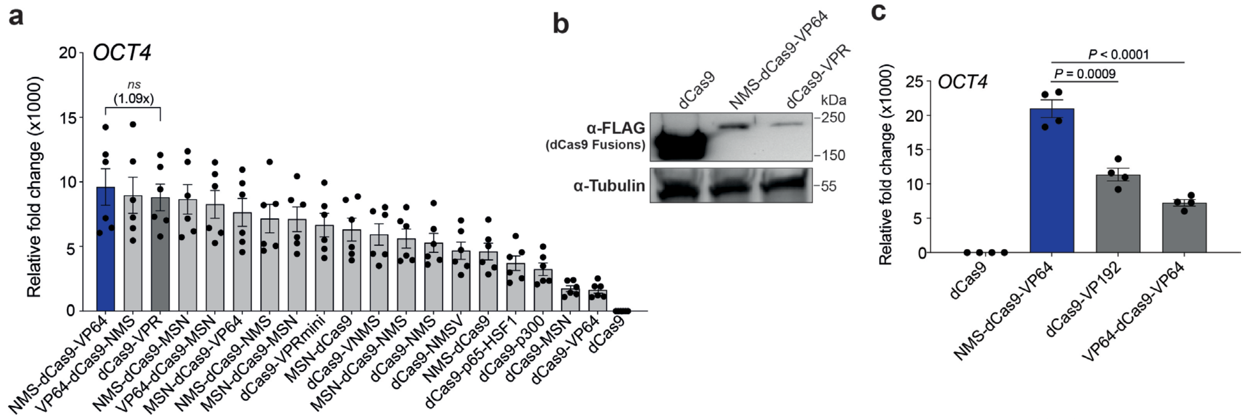


**d**

| dCas9 Fusion | N-Term | C-term | Total |
|---|---|---|---|
| NMS-dCas9-VP64 | 870 bp | 150 bp | 1020 bp |
| VP64-dCas9-NMS | 150 bp | 870 bp | 1020 bp |
| dCas9-VPR | | 1569 bp | 1569 bp |
| NMS-dCas9-MSN | 870 bp | 870 bp | 1740 bp |
| VP64-dCas9-MSN | 150 bp | 870 bp | 1020 bp |
| MSN-dCas9-VP64 | 870 bp | 150 bp | 1020 bp |
| NMS-dCas9-NMS | 870 bp | 870 bp | 1740 bp |
| MSN-dCas9-MSN | 870 bp | 870 bp | 1740 bp |
| dCas9-VPRmini | | 894 bp | 894 bp |
| MSN-dCas9 | 870 bp | | 870 bp |
| dCas9-VNMS | | 1026 bp | 1026 bp |
| MSN-dCas9-NMS | 870 bp | 870 bp | 1740 bp |
| dCas9-NMS | | 870 bp | 870 bp |
| dCas9-NMSV | | 1026 bp | 1026 bp |
| NMS-dCas9 | 870 bp | | 870 bp |
| dCas9-p65-HSF1 | | 939 bp | 939 bp |
| dCas9-p300-core | | 1851 bp | 1851 bp |
| dCas9-MSN | | 870 bp | 870 bp |
| dCas9-VP64 | | 150 bp | 150 bp |
| VP64-dCas9-VP64 | 150 bp | 150 bp | 300 bp |
| dCas9-VP192 | | 597 bp | 597 bp |

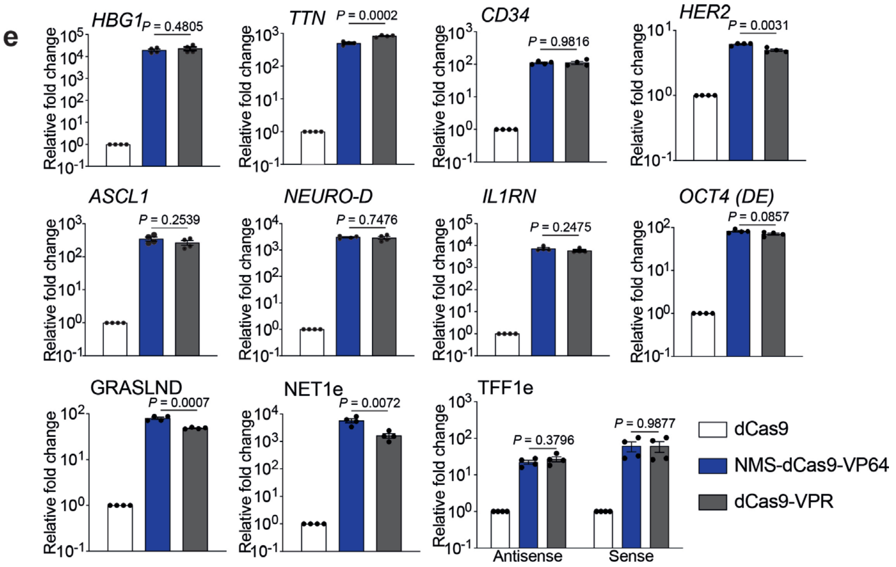


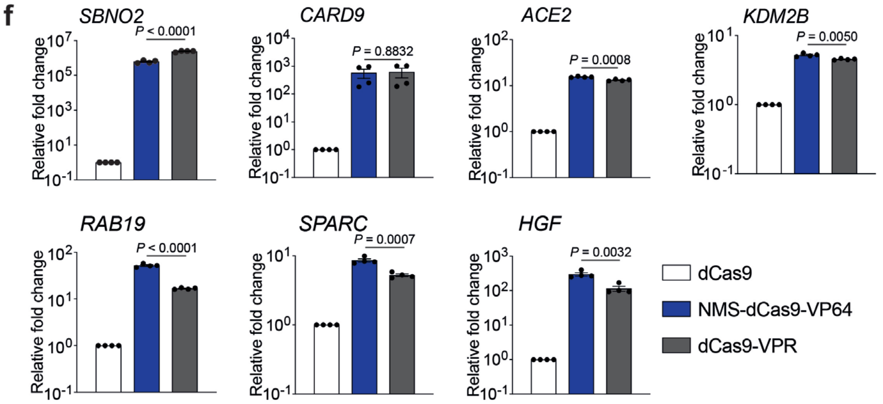


**Supplementary Fig. 26. Generating and validating tripartite TADs in direct fusion architectures. a.** *OCT4* mRNA levels after different dCas9 direct fusions were targeted to the *OCT4* promoter using pooled gRNAs. Indicated direct fusions were generated by linking MSN or NMS domains to either the C-terminus, N-terminus, or both termini of dCas9, along with selected combinations also containing VP64 as indicated. **b.** The

expression levels of dCas9, NMS-dCas9-VP64, dCas9-VPR are shown as detected by Western blotting in HEK293T cells 72 hours post-transfection. **c.** *OCT4* mRNA levels after NMS-dCas9-VP4 and other VP64-based dCas9 fusion (VP64-dCas9-VP64 and dCas9-VP192) were targeted to the *OCT4* promoter using pooled gRNAs. **d.** Lengths (in bp) of different fusion proteins and modules are shown. **e.** Relative expression levels of 11 different endogenous human coding and noncoding loci after dCas9, NMS-dCas9-VP64, or dCas9-VPR systems were targeted to their respective promoters/enhancers using pooled gRNAs. **f.** Relative expression levels of 7 different endogenous human genes after dCas9, NMS-dCas9-VP64, or dCas9-VPR systems were targeted to their respective promoters/enhancers using single gRNAs. All samples were processed for QPCR analysis 72 – 84 hours post-transfection in HEK293T cells. Data are the result of 6 biological replicates for **panel a,** and 4 biological replicates for **panels c, e and f.** Western blot data shown in **panel b** was from two independent experiments. See source data for more information. Data are presented as mean values +/- SEM. *P* values determined using unpaired two-sided t-test.

**Supplementary Figure 27**

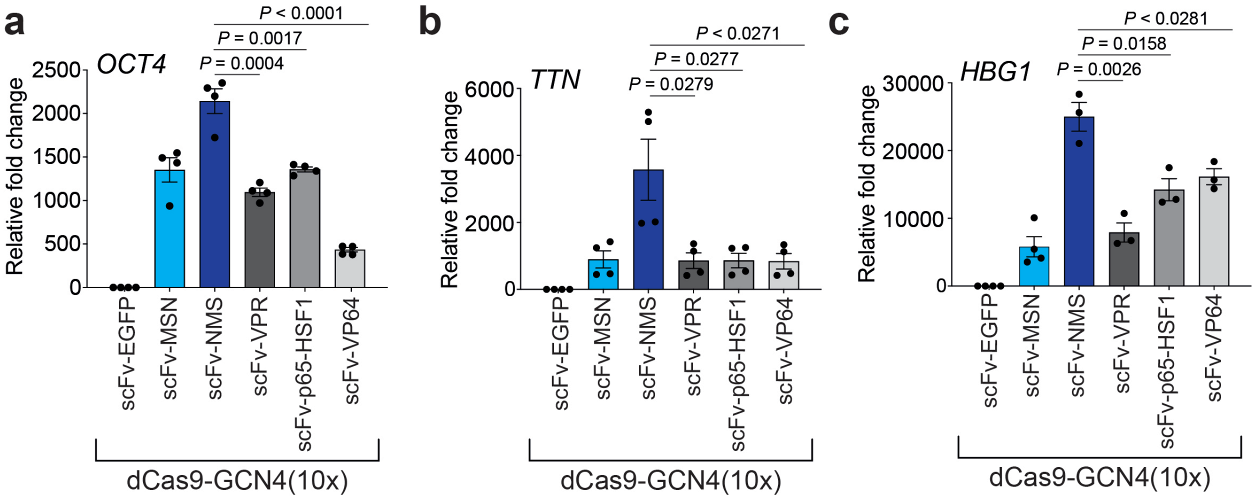


**Supplementary Fig. 27. The NMS effector domain is compatible with, and robust in, the dCas9 SunTag system. a – c.** *OCT4* (**panel a**), *TTN* (**panel b**) and *HBG1* (**panel c**) mRNA levels after the indicated TADs were fused to scFv and recruited via dCas9 harboring a 10xGCN4 C-terminal fusion protein (the SunTag system) along with 4 pooled gRNAs targeting each respective promoter. All samples were processed for QPCR analysis 72 hours post-transfection in HEK293T cells. Data are the result of 4 biological replicates for **panels a, and b,** and 3 biological replicates for **panel c.** See source data for more information. Data are presented as mean values +/- SEM. *P* values determined using unpaired two-sided t-test.

**Supplementary Figure 28**

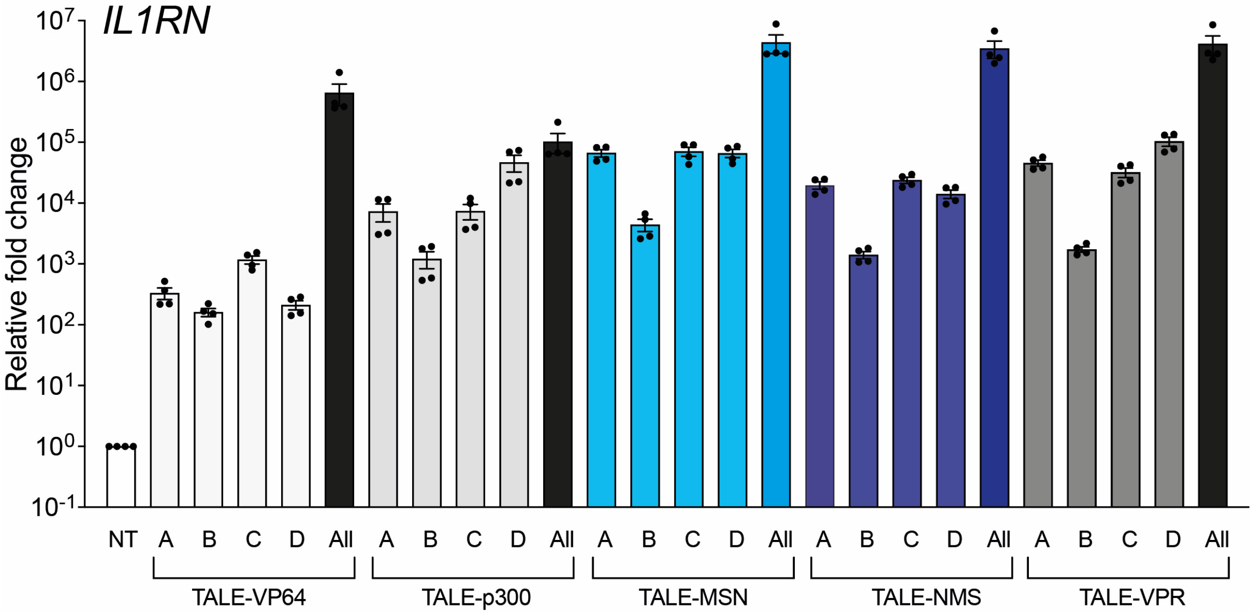


**Supplementary Fig. 28. The tripartite MSN and NMS TADs are portable to synthetic TALE DNA binding systems. a.** Relative *IL1RN* mRNA levels after individual (A – D) or pooled ("all") *IL1RN* promoter-targeting TALEs (TALE-VP64, TALE-p300, TALE-MSN, TALE-NMS and TALE-VPR) were co-transfected into HEK293T cells. All samples were processed for QPCR analysis 72 hours post-transfection in HEK293T cells. Data are the result of 4 biological replicates. See source data for more information. Data are presented as mean values +/- SEM.

**Supplementary Figure 29**

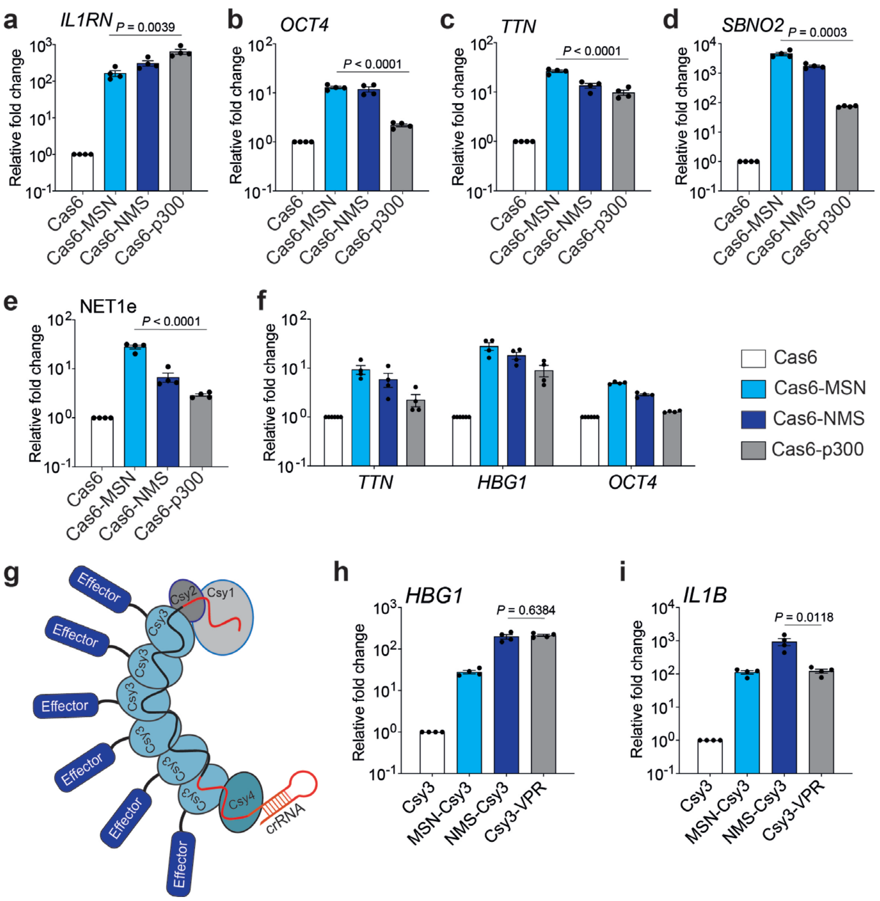


**Supplementary Fig. 29. The tripartite MSN and NMS TADs are portable to different Type I CRISPRa systems. a – d.** *IL1RN* (**panel a**), *OCT4* (**panel b**), *TTN* (**panel c**) or *SBNO2* (**panel d**) mRNA activation when the indicated Cas6 fusion protein encoding plasmids (and plasmids encoding the other components of Eco-cascade complex) were targeted to each corresponding promoter using a single crRNA. **e.** NET1 eRNA activation when the indicated Cas6 fusion protein encoding plasmids (and plasmids encoding the other components of Eco-cascade complex) were targeted to respective enhancer using a single crRNA. **f.** Multiplexed activation of 3 protein coding genes (*TTN*, *HBG1* and *OCT4*) following co-transfection of the indicated Cas6 fusion protein encoding plasmids (and plasmids encoding the other components of Eco-cascade complex) and a multiplexed crRNA expression plasmid encoding 1 crRNA/locus. **g.** The Type I CRISPR system derived from *P. aeruginosa* (*Pae*-Cascade) is schematically depicted along with a representative effector fused to the Csy3 protein subunit. **h – i.** *HBG1* (**panel h**) and *IL1B* (**panel i)** mRNA activation using the indicated Csy3 fusion proteins when targeted to each corresponding promoter using a single crRNA, respectively. All samples were processed for QPCR analysis 72 hours post-transfection in HEK293T cells. Data are the result of 4 biological replicates for **panels a – f, h and i**. See source data for more information. Data are presented as mean values +/- SEM. *P* values determined using unpaired two-sided t-test.

**Supplementary Figure 30**

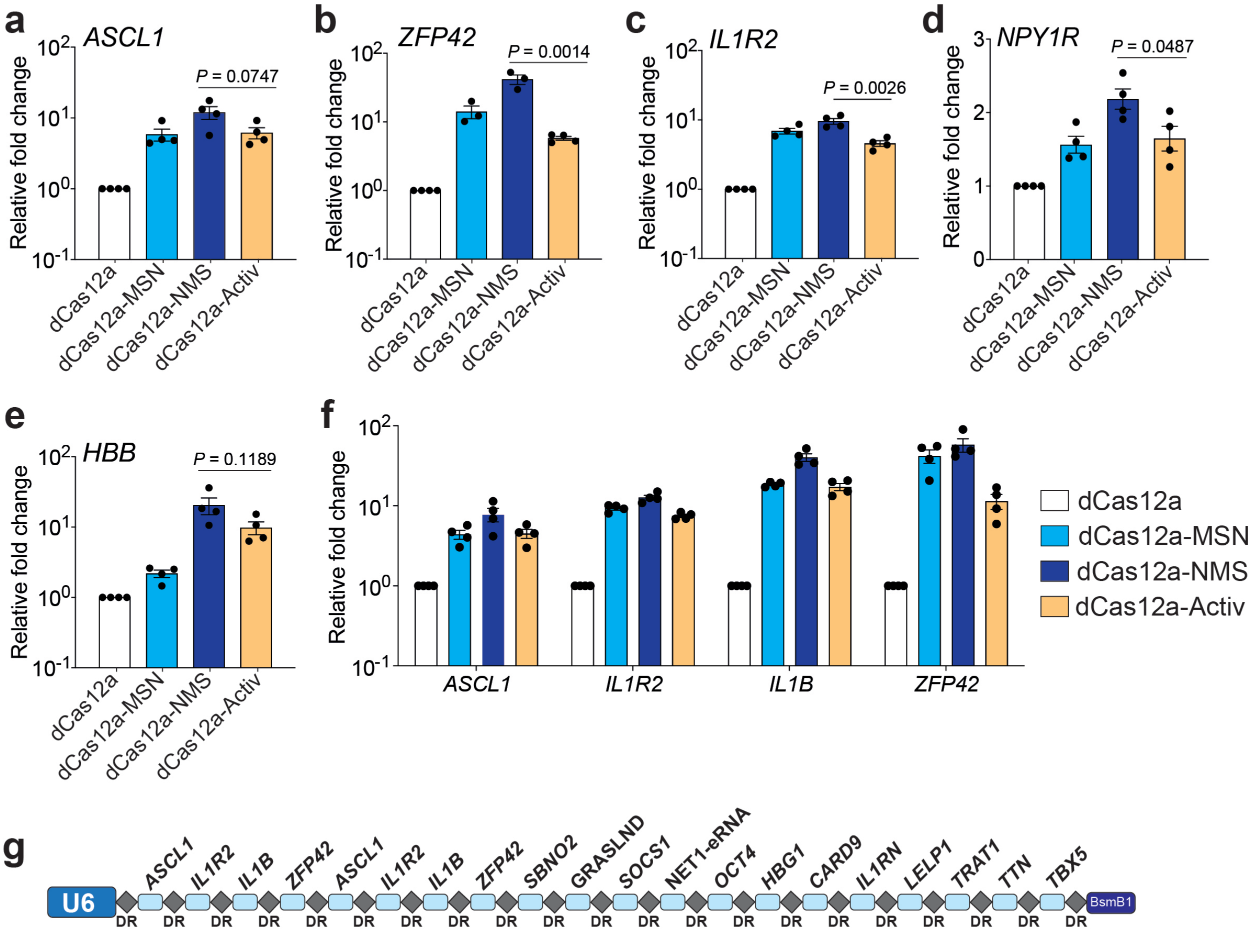


**Supplementary Fig. 30. The tripartite MSN and NMS TADs are portable to the dCas12a-based CRISPRa system. a – c.** *ASCL1* (**panel a**), *ZFP42/REX1* (**panel b**) *and IL1R2* (**panel c**) transactivation after the indicated dCas12a fusion proteins were targeted to each corresponding promoter using 2 crRNAs per respective locus. **d – e.** *NPY1R* (**panel d**) and *HBB* (**panel e**) mRNA activation after the indicated dCas12a fusion proteins were targeted to each corresponding promoter using a single crRNA. **f**. Multiplexed activation of 4 indicated endogenous genes 72 hours after co-transfection of indicated dCas12a fusion protein encoding plasmids and a single plasmid encoding an 8-crRNA expression array (2 crRNAs/gene promoter). **g**. The crRNA expression array encoding 20 crRNA targeting 16 loci used in main text **Figure 4i**, is schematically depicted. All samples were processed for QPCR analysis 72 hours post-transfection in HEK293T cells. Data are the result of 4 biological replicates for **panels a, c – f, h** and 3 - 4 biological replicates for **panel b**. See source data for more information. Data are presented as mean values +/- SEM. *P* values determined using unpaired two-sided t-test.

## Supplementary Figure 31

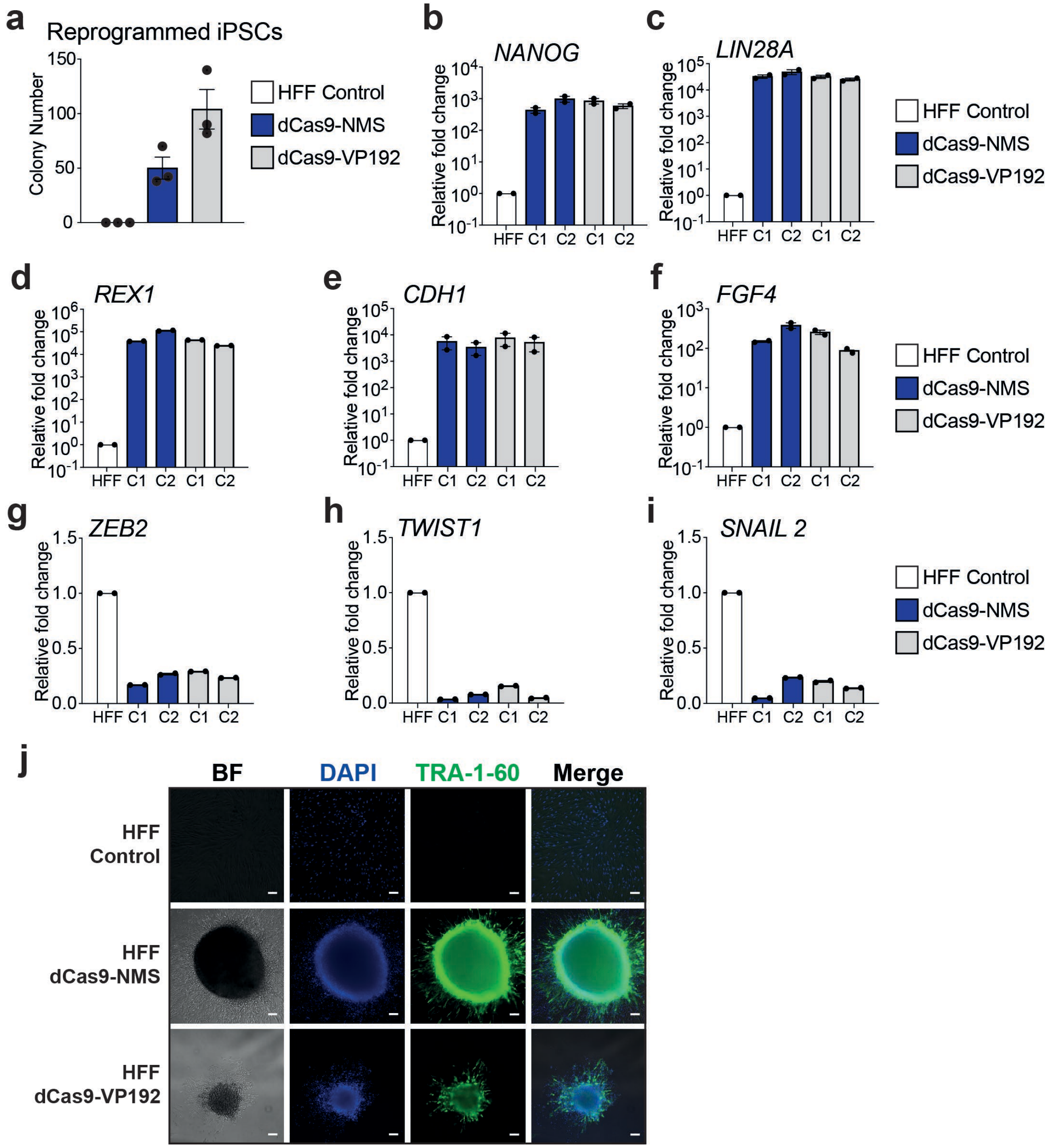


**Supplementary Fig. 31. dCas9-NMS permits efficient *in vitro* reprogramming of human fibroblasts. a.** Bar graph showing iPSC colonies (the total number of colonies per million HFFs) generated from HFFs nucleofected with either dCas9-NMS and dCas9-VP192 and a gRNA cocktail (see main text and methods). **b – f.** Relative expression of pluripotency-associated genes *NANOG* (**panel b**), *LIN28A* (**panel c**), *REX1* (**panel d**), *CDH1* (**panel e**), *FGF4* (**panel f**) in representative colonies (C1 or C2) ~40 days after nucleofection of either dCas9-NMS (blue) or dCas9-VP192 (gray) and multiplexed gRNAs compared to untreated HFF controls. **g – i.** Relative expression of mesenchymal-

associated genes *ZEB2* (**panel g**), *TWIST1* (**panel h**) and *SNAIL2* (**panel i**), in representative colonies (C1 or C2) ~40 days after nucleofection of either dCas9-NMS (blue) or dCas9-VP192 (gray) and multiplexed gRNAs compared to untreated HFF controls. **j.** Immunofluorescence microscopy of HFFs ~40 days after nucleofection of either dCas9-NMS or dCas9-VP192 and multiplexed gRNAs compared to untreated HFF controls (white scale bars, 100μm). Cells were immunostained for the expression of pluripotency-associated cell surface marker TRA-1-60 (**panel j**, green). All cells were counterstained with DAPI to visualize the nucleus. Data are the result of 3 independent experiments for **panels b – i,** two independent measurements from independent subclones per colony. Data are presented as mean values +/- SEM.

## Supplementary Figure 32

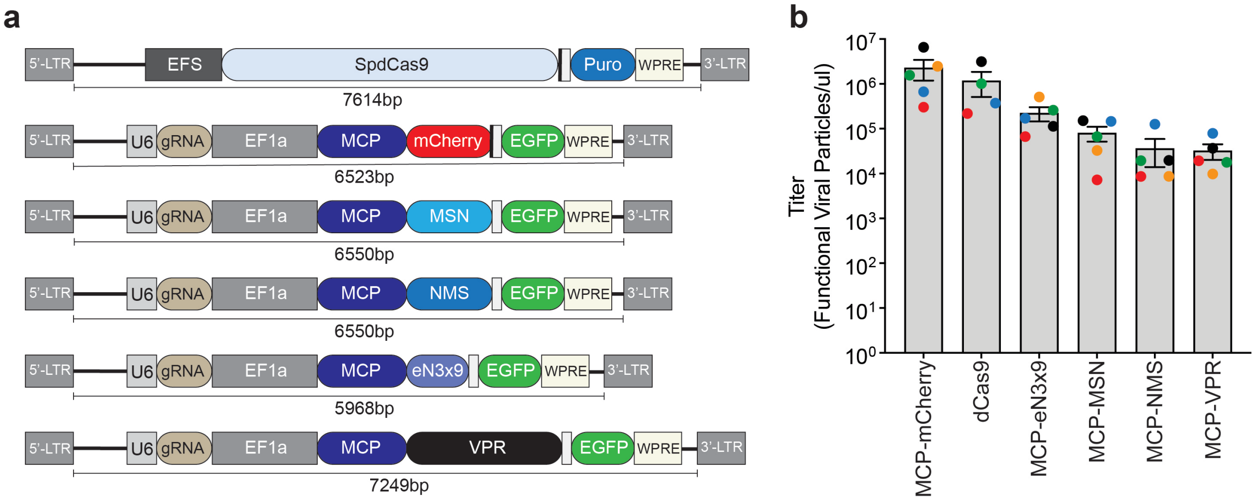


**Supplementary Fig. 32. Lentiviral titers are influenced by the presence of TAD domains. a**. Schematic representation of different lentiviral vectors with their respective sizes shown (in bp). **b**. Titers of different lentiviruses used in this study. Each colored dot indicates a lentiviral titer from an independent preparation. Data are the result of 4-5 independent experiments for **panel b**. Data are presented as mean values +/- SEM.

## Supplementary Figure 33

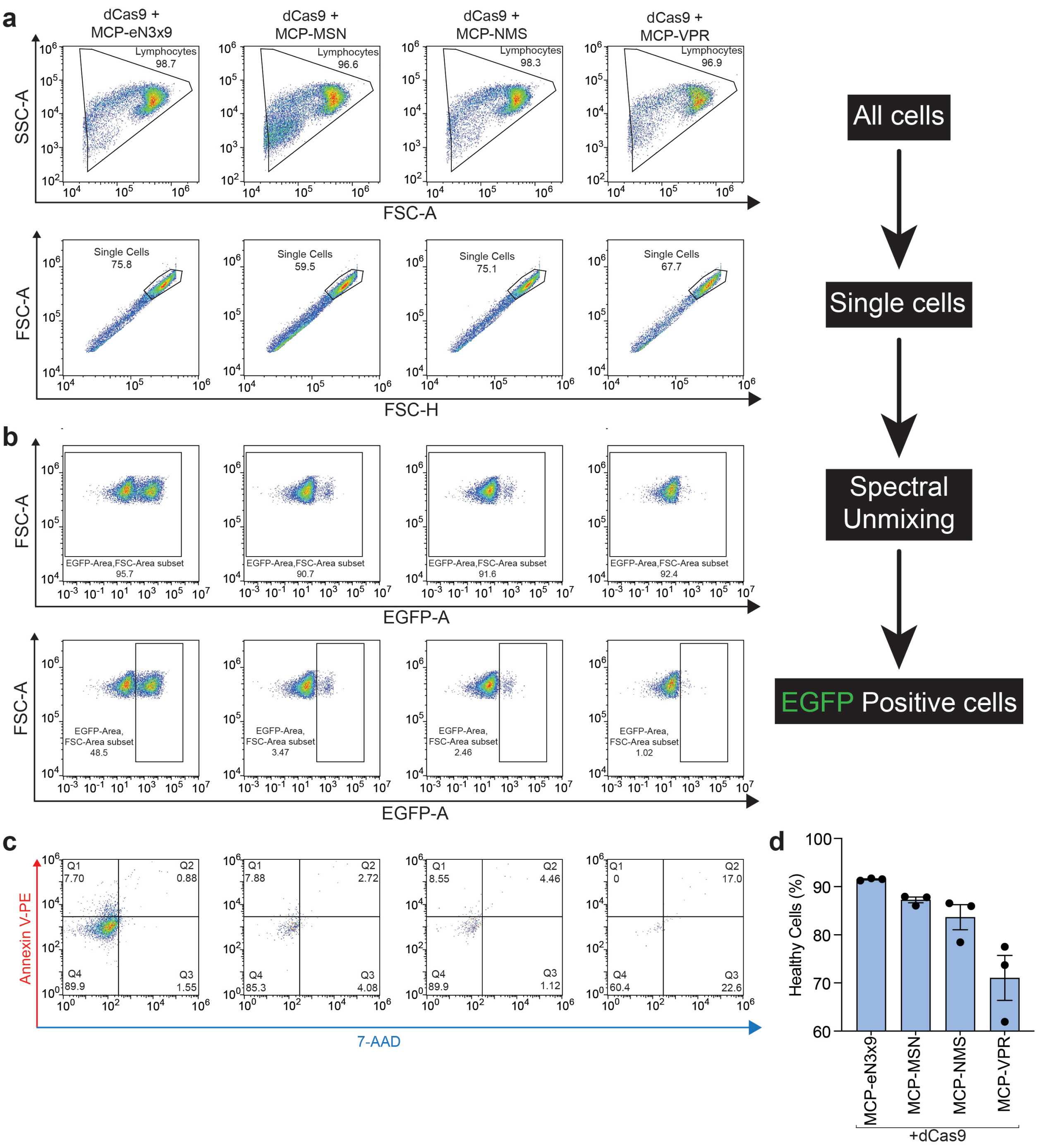


**Supplementary Fig. 33. Engineered TADs from MTFs exhibit lower toxicity compared to VPR in primary T cells. a.** Density plots indicating gating for bulk (top) and single (bottom) primary human T cells after cells were transduced with dCas9 along with MCP-eN3x9 (column 1), MCP-MSN (column 2), MCP-NMS (column 3), or MCP-VPR (column 4). All MCP-fusion encoding vectors also encode an MS2-modifed gRNA targeting human *TTN*. **b.** Density plots indicating the gating strategy to remove events

with EGFP signal < $1x10^{-3}$ resulting from spectral unmixing (top) and to select EGFP positive cells (bottom). **c.** Flow cytometry plot showing $7AAD^{+}$/Annexin $V^{+}$ cells (after enriching for EGFP positive cells) co-transduced with indicated lentiviral vectors. **d**. Bar graph showing the percentage of healthy primary T cells among the EGFP positive (from flow cytometry data) transduced with indicated lentiviral particles. All experiments were performed in triplicate and density plots shown are representative of all replicates. See source data for more information. Representative density plot (**panels a and b**) and flow cytometry (**panel c**) are from 3 biological replicates (and each biological replicate with technical duplicates). Data are the result of are from 3 biological replicates (and each biological replicate with technical duplicates) for **panel d**. Data are presented as mean values +/- SEM.

**Supplementary Figure 34**

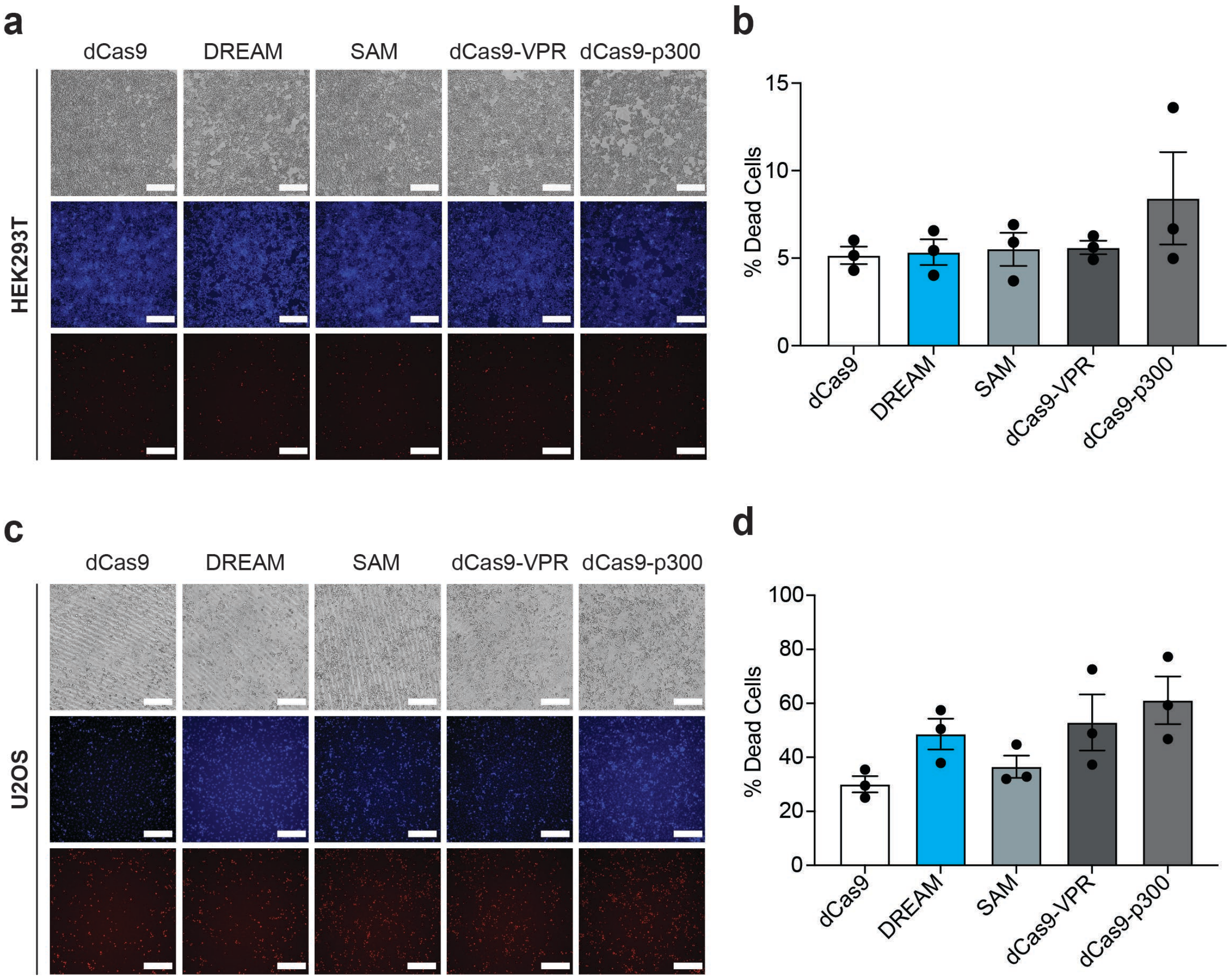


**Supplementary Fig. 34. Comparison of toxicity of different CRISPRa platforms in cultured adherent cell lines. a.** Microscopy images showing brightfield, Hoechst, and 7-AAD staining of HEK293T cells 48 hours post-transfection of indicated CRISPRa tools in presence of 4 pooled gRNAs (MS2 modified for DREAM and SAM systems) targeting *HBG1* (white scale bars, 250μm). **b.** Bar graph showing percentage of dead cells obtained from quantification of microscopic images HEK293T cells. **c.** Microscopy images showing brightfield, Hoechst, and 7-AAD staining of U2OS cells 48 hours post-transfection of indicated CRISPRa tools in presence of 4 pooled gRNAs (MS2 modified for DREAM and SAM systems) targeting *HBG1* (white scale bars, 250μm). **d.** Bar graph showing percentage of dead cells obtained from quantification of microscopic images in U2OS cells. Microscopic images (**panels a and c**) are representative of 3 biological replicates. Data are the result of are from 3 biological replicates for **b panel d**. Data are presented as mean values +/- SEM.

## Supplementary Figure 35

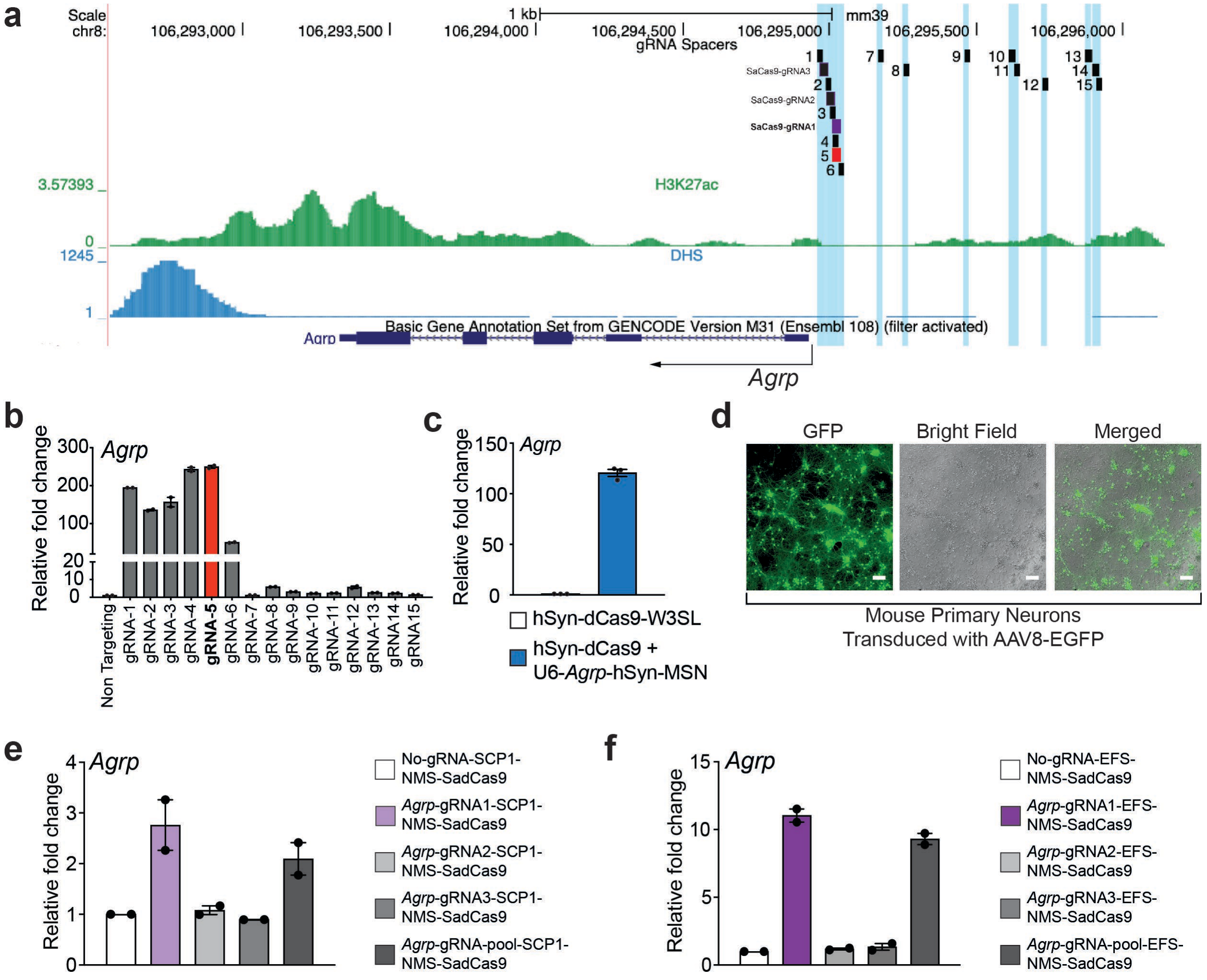


**Supplementary Fig. 35. Selection and validation of optimal *Agrp* gRNA and construct designs for dual and all-in-one (AIO) AAV vectors. a and b.** The genomic region (mm10) encompassing the mouse *Agrp* gene on chromosomes 8 is shown. *Agrp* gene is shown in dark blue; SpdCas9 gRNA (1-15) target regions are indicated by black lines and light blue highlighting except for the most potent gRNA (gRNA 5), which was selected for future experiments and is shown in red. SadCas9 gRNA (1-3) target regions are indicated by black lines and light blue highlighting except most potent gRNA1, which was selected for future experiments was shown in purple. H3K27ac (from GSE10666), and DNase Hypersensitivity Sites (DHSs; from GSE37074) are shown in green, and blue, respectively. Transcription Start Sites (TSSs) for *Agrp* is indicated by black arrows. **b**. Comparison of transactivation potency of different gRNA targeting *Agrp* promoter using the DREAM system. 15 gRNAs were designed to tile across ~1.5kb upstream of the *Agrp* promoter. Non-transfected Neuro-2A cells were used as a control. **c**. Dual AAV plasmid (comprising CRISPR-DREAM and *Agrp* gRNA5) mediated *Agrp* induction in Neuro-2A cells. **d**. Immunofluorescence microscopy showing EGFP expression in AAV8-EGFP transduced (2.5 X $10^4$ vg/ cell) mouse primary cortical neurons 72 hours post-transduction

(white scale bars, 100μm). **e**. *Agrp* induction in Neuro-2A cells 72 hours after transfection of the AIO AAV construct consisting of the SCP1 promoter driven NMS-SadCas9. **f**. *Agrp* induction in Neuro-2A cells 72 hours after transfection of the AIO AAV construct consisting of EFS promoter driven NMS-SadCas9. Data are the result of 2 biological replicates for **panel b, e, and f** and 3 biological replicates for **panel c.** Microscopic images shown in **panel d** are representative of 4 biological replicates. Data are presented as mean values +/- SEM.

## Supplementary Table 1: *Streptococcus pyogenes* Cas9 Protospacer sequences used in this study.

| Target Gene | Protospacer Sequence (SpCas9) (5'-3') | Genomic Location (GRCh38/hg38 Assembly) | Reference |
|---|---|---|---|
| *OCT4*_Promoter-1 | ACTCCACTGCACTCCAGTCT | chr6:31,170,934-31,170,953 | Hu et al., Nucleic Acids Res., 2014 |
| *OCT4*_Promoter-2 | TCTGTGGGGGACCTGCACTG | chr6:31,170,866-31,170,885 | Hu et al., Nucleic Acids Res., 2014 |
| *OCT4*_Promoter-3 | GGGGCGCCAGTTGTGTCTCC | chr6:31,170,836-31,170,855 | Hu et al., Nucleic Acids Res., 2014 |
| *OCT4*_Promoter-4 | ACACCATTGCCACCACCATT | chr6:31,170,797-31,170,816 | Hu et al., Nucleic Acids Res., 2014 |
| *OCT4*_DE_1 | GGAGGAACATGCTTCGGAAC | chr6:31,173,032-31,173,051 | Hilton et al., Nat Biotech, 2015 |
| *OCT4*_DE_2 | GGTCTGCCGGAAGGTCTACA | chr6:31,172,930-31,172,949 | Hilton et al., Nat Biotech, 2015 |
| *OCT4*_DE_3 | GCATGACAAAGGTGCCGTGA | chr6:31,173,098-31,173,117 | Hilton et al., Nat Biotech, 2015 |
| *IL1RN*_Promoter-1 | TGTACTCTCTGAGGTGCTC | chr2:113,117,865-113,117,883 | Perez-Pinera et al., Nat. Methods, 2013 |
| *IL1RN*_Promoter-2 | ACGCAGATAAGAACCAGTT | chr2:113,117,714-113,117,732 | Perez-Pinera et al., Nat. Methods, 2013 |
| *IL1RN*_Promoter-3 | CATCAAGTCAGCCATCAGC | chr2:113,117,781-113,117,799 | Perez-Pinera et al., Nat. Methods, 2013 |
| *IL1RN*_Promoter-4 | GAGTCACCCTCCTGGAAAC | chr2:113,117,749-113,117,767 | Perez-Pinera et al., Nat. Methods, 2013 |
| *TTN*_Promoter-1 | CCTTGGTGAAGTCTCCTTTG | chr2:178,807,596-178,807,615 | Chavez et al., Nat Methods, 2015 |
| *TTN*_Promoter-2 | ATGTTAAAATCCGAAAATGC | chr2:178,807,676-178,807,695 | Chavez et al., Nat Methods, 2015 |
| *TTN*_Promoter-3 | GGGCACAGTCCTCAGGTTTG | chr2:178,807,753-178,807,772 | Chavez et al., Nat Methods, 2015 |
| *TTN*_Promoter-4 | ATGAGCTCTCTTCAACGTTA | chr2:178,807,907-178,807,926 | Chavez et al., Nat Methods, 2015 |
| *TTN*_Promoter-5 | GCAAAATTGGAACTCTGCTT | chr2:178,808,032-178,808,051 | This Study |
| *TTN*_Promoter-6 | TAAGACCTGGCTACTTACTA | chr2:178,808,142-178,808,161 | This Study |
| *TTN*_Promoter-7 | CTGGAGTTTGCTGTTACTAA | chr2:178,808,220-178,808,239 | This Study |
| *TTN*_Promoter-8 | GTGAAACTCTACTTAGAGGG | chr2:178,808,363-178,808,382 | This Study |
| *TTN*_Promoter-9 | GGCCAAATTCTTCCATAACA | chr2:178,808,488-178,808,507 | This Study |
| *TTN*_Promoter-10 | AGATACCACCACTTAAGTAA | chr2:178,808,668-178,808,687 | This Study |
| *FOXA3*_Promoter-1 | GGCACGGCCCGAGCTCCCAC | chr19:45,864,366-45,864,385 | This Study |
| *FOXA3*_Promoter-2 | AGGCGGCCACGCTTTATAGC | chr19:45,864,294-45,864,313 | This Study |
| *FOXA3*_Promoter-3 | GGGGAGCCCGGGGCGGGCGA | chr19:45,864,258-45,864,277 | This Study |
| *FOXA3*_Promoter-4 | GTCCGAGCGCGCCGCCCTCG | chr19:45,864,227-45,864,246 | This Study |
| *FOXA3*_Promoter-5 | CAGCCCCAGGACGCGACAGC | chr19:45,864,154-45,864,173 | This Study |
| *FOXA3*_Promoter-6 | AGCGACGCTGCAGATACCAA | chr19:45,864,101-45,864,120 | This Study |
| *FOXA3*_Promoter-7 | TCTGGAGCCTTTCTACCTCA | chr19:45,864,063-45,864,082 | This Study |
| *FOXA3*_Promoter-8 | TGGAGCCTTTCTACCTCAGG | chr19:45,864,061-45,864,080 | This Study |
| *FOXA3*_Promoter-9 | AAAGGGGAATCAGAACGCTA | chr19:45,863,979-45,863,998 | This Study |
| *FOXA3*_Promoter-10 | TCCTGCAGTGCTGGAATTAC | chr19:45,863,831-45,863,850 | This Study |
| *HBG1/2*_Promoter-1 | GCTAGGGATGAAGAATAAA | chr11:5,254,807-5,254,825 | Perez-Pinera et al., Nat. Methods, 2013 |
| *HBG1/2*_Promoter-2 | TTGACCAATAGCCTTGACA | chr11:5,254,882-5,254,901 | Perez-Pinera et al., Nat. Methods, 2013 |
| *HBG1/2*_Promoter-3 | TGCAAATATCTGTCTGAAA | chr11:5,254,944-5,254,963 | Perez-Pinera et al., Nat. Methods, 2013 |
| *HBG1/2*_Promoter-4 | AAATTAGCAGTATCCTCTT | chr11:5,250,066-5,250,085 | Perez-Pinera et al., Nat. Methods, 2013 |
| *CD34*_Promoter-1 | GAAAGCTGAACGAGGCATC | chr1:207,911,360-207,911,378 | Johnston et al., CRISPR J, 2020 |
| *CD34*_Promoter-2 | CCTTTTGCAAGATTGTTAC | chr1:207,911,538-207,911,556 | Johnston et al., CRISPR J, 2020 |
| *CD34*_Promoter-3 | CACTAAATGTGCCACATTG | chr1:207,911,621-207,911,639 | Johnston et al., CRISPR J, 2020 |
| *CD25*_Promoter-1 | AGTTCAATTGCTGGAGGTGT | chr10:6,062,477-6,062,496 | This Study |
| *CD25*_Promoter-2 | CTTGCTCACCCTACCTTCAA | chr10:6,062,585-6,062,604 | This Study |
| *CD25*_Promoter-3 | ATAAGCTGAGTCCTCCCCTC | chr10:6,062,637-6,062,656 | Simeonov et al., Nature, 2017 |
| *CD25*_Promoter-4 | GTCCTGACAAGATTCTGGTC | chr10:6,062,711-6,062,730 | Simeonov et al., Nature, 2017 |

| | | | |
|---|---|---|---|
| *TRK-C*_Promoter-1 | GAGGAGGCCGCGACGAGGGA | chr15:88,256,818-88,256,837 | This Study |
| *TRK-C*_Promoter-2 | CAACTTTTTCCCTTCGGTCT | chr15:88,257,068-88,257,087 | This Study |
| *TRK-C*_Promoter-3 | GTTCATTTCACTTCCTACCG | chr15:88,257,214-88,257,233 | This Study |
| *NEURO-D*_Promoter-1 | AGGGGAGCGGTTGTCGGAGG | chr2:181,680,699-181,680,718 | Chavez et al., Nat Methods, 2015 |
| *NEURO-D*_Promoter-2 | ACCTGCCCATTTGTATGCCG | chr2:181,680,810-181,680,829 | Chavez et al., Nat Methods, 2015 |
| *NEURO-D*_Promoter-3 | AGGTCCGCGGAGTCTCTAAC | chr2:181,680,887-181,680,906 | Chavez et al., Nat Methods, 2015 |
| *NEURO-D*_Promoter-4 | TAGAGGGGCCGACGGAGATT | chr2:181,681,243-181,681,262 | Chavez et al., Nat Methods, 2015 |
| *RHOXF2*_Promoter-1 | ACGCGTGCTCTCCCTCATC | chrX:120,077,787-120,077,805 | Chavez et al., Nat Methods, 2015 |
| *RHOXF2*_Promoter-2 | CTGTGGGTTGGGCCTGCTG | chrX:120,157,899-120,157,918 | Chavez et al., Nat Methods, 2015 |
| *RHOXF2*_Promoter-3 | ATTAATAGTGGACTTTCTCA | chrX:120,078,107-120,078,126 | Chavez et al., Nat Methods, 2015 |
| *MYOD*_Promoter-1 | CCTGGGCTCCGGGGCGTTT | chr11:17,719,508-17,719,527 | Perez-Pinera et al, Nat Methods. 2013 |
| *MYOD*_Promoter-2 | GGCCCCTGCGGCCACCCCG | chr11:17,719,422-17,719,439 | Perez-Pinera et al, Nat Methods. 2013 |
| *MYOD*_Promoter-3 | CTCCCTCCCTGCCCGGTAG | chr11:17,719,350-17,719,368 | Perez-Pinera et al, Nat Methods. 2013 |
| *MYOD*_Promoter-4 | AGGTTTGGAAAGGGCGTGC | chr11:17,719,290-17,719,308 | Perez-Pinera et al, Nat Methods. 2013 |
| *MYOD*_DRR-1 | TGTTTTCAGCTTCCAAACT | chr11:17,714,981-17,714,999 | Hilton et al., Nat Biotech, 2015 |
| *MYOD*_DRR-2 | CATGAAGACAGCAGAAGCC | chr11:17,714,764-17,714,782 | Hilton et al., Nat Biotech, 2015 |
| *MYOD*_DRR-3 | GGCCCACATTCCTTTCCAG | chr11:17,714,611-17,714,629 | Hilton et al., Nat Biotech, 2015 |
| *MYOD*_DRR-4 | GGCTGGATTGGGTTTCCAG | chr11:17,714,517-17,714,536 | Hilton et al., Nat Biotech, 2015 |
| *ASCL1*_Promoter-1 | CGGGAGAAAGGAACGGGAGG | chr12:102,957,473-102,957,492 | Chavez et al., Nat Methods, 2015 |
| *ASCL1*_Promoter-2 | AAGAACTTGAAGCAAAGCGC | chr12:102,957,232-102,957,251 | Chavez et al., Nat Methods, 2015 |
| *ASCL1*_Promoter-3 | TCCAATTTCTAGGGTCACCG | chr12:102,957,097-102,957,116 | Chavez et al., Nat Methods, 2015 |
| *ASCL1*_Promoter-4 | GTTGTGAGCCGTCCTGTAGG | chr12:102,956,797-102,956,816 | Chavez et al., Nat Methods, 2015 |
| *HER2*_Promoter-1 | GAATTTATCCCGGACTCCG | chr17:39,699,761-39,699,779 | O'Geen et al., Nucleic Acid Res., 2017 |
| *HER2*_Promoter-2 | GTTGGAATGCAGTTGGAGG | chr17:39,699,942-39,699,960 | O'Geen et al., Nucleic Acid Res., 2017 |
| *HER2*_Promoter-3 | ATTCCAGAAGATATGCCCC | chr17:39,699,548-39,699,566 | O'Geen et al., Nucleic Acid Res., 2017 |
| *TRK-A*_Promoter-1 | GACGGGAATGTGGAATGCAC | chr1:156,815,673-156,815,692 | This Study |
| *TRK-A*_Promoter-2 | GATGCAGGAAGTCACCCTAG | chr1:156,815,562-156,815,581 | This Study |
| *TRK-A*_Promoter-3 | AATTGTACCCTAAAATAGAC | chr1:156,815,327-156,815,346 | This Study |
| *TRK-B*_Promoter-1 | CTCGGAAGTGTCAGGCACTG | chr9:84,668,467-84,668,486 | This Study |
| *TRK-B*_Promoter-2 | AGGGAACGTAAAACATCTAT | chr9:84,668,088-84,668,107 | This Study |
| *TRK-B*_Promoter-3 | TCTATCTCACCACTGTCACT | chr9:84,667,966-84,667,985 | This Study |
| *CARD9*_Promoter-1 | TGGGAGCAGCTTTCCTCTGG | chr9:136,373,785-136,373,804 | Morita et al, IJMS, 2020 |
| *KDM2B*_Promoter-1 | GGACTGCAAAGGCTCTGGTC | chr12:121,581,038-121,581,057 | Morita et al, IJMS, 2020 |
| *RAB19*_Promoter-1 | GCCTCAGGGTACAAAGCGCC | chr7:140,403,994-140,404,013 | Morita et al, IJMS, 2020 |
| *SBNO2*_Promoter-1 | GGTAATTGCCCTGACTTACG | chr19:1,132,346-1,132,365 | Morita et al, IJMS, 2020 |
| *SPARC*_Promoter-1 | GAAGGGCCAAGCAATTCAAG | chr5:151,687,366-151,687,385 | Morita et al, IJMS, 2020 |
| *HGF*_Promoter-1 | GCTCCTATCCGAGTAAGGAA | chr7:81,399,671-81,399,690 | Morita et al, IJMS, 2020 |
| *ACE2*_Promoter-1 | TCATGAGGAGGTTTTAGTCT | chrX:15,600,956-15,600,975 | This Study |
| *TBX5*_Promoter-1 | GTTCTCCGTAATGTGCCTTG | chr12:114,408,620-114,408,639 | Wang et al., Acta Pharm. Sin. B., 2020 |
| *HS2*_Enhancer-1 | AATATGTCACATTCTGTCTC | chr11:5,280,570-5,280,589 | Hilton et al., Nat Biotech, 2015 |
| *HS2*_ Enhancer-2 | GGACTATGGGAGGTCACTAA | chr11:5,280,878-5,280,897 | Hilton et al., Nat Biotech, 2015 |
| *HS2*_ Enhancer -3 | GAAGGTTACACAGAACCAGA | chr11:5,280,803-5,280,822 | Hilton et al., Nat Biotech, 2015 |
| *HS2*_ Enhancer -4 | GCCCTGTAAGCATCCTGCTG | chr11:5,280,668-5,280,687 | Hilton et al., Nat Biotech, 2015 |
| *SOCS1* +15kb Enhancer -1 | GATTTCTAAGAAACCAGTTG | chr16:11,240,852-11,240,871 | This Study |

| | | | |
|---|---|---|---|
| *SOCS1* +15kb Enhancer -2 | TTCTGGAGCTGGAGCAAAGC | chr16:11,240,925-11,240,944 | This Study |
| *SOCS1* +15kb Enhancer -3 | GATTTCTAAGAAACCAGTTG | chr16:11,240,852-11,240,871 | This Study |
| *SOCS1* +50kb Enhancer -1 | CTTGACCTGGAAGACTCAGC | chr16:11,206,702-11,206,721 | This Study |
| *SOCS1* +50kb Enhancer -2 | CTGAGAACGACCCCTAGGAG | chr16:11,206,779-11,206,798 | This Study |
| *KLK3*_Enhancer-1 | CTGGTCACCCTACAAGATTT | chr19:50,850,653-50,850,672 | This Study |
| *KLK3*_Enhancer-2 | CTGGTGAGAAACCTGAGATT | chr19:50,850,713-50,850,732 | This Study |
| *KLK3*_Enhancer-3 | GTTGTCCCAGTATAAGATTG | chr19:50,850,742-50,850,761 | This Study |
| *KLK3*_Enhancer-4 | GATTGAAAACAGACCTACTC | chr19:50,850,816-50,850,835 | This Study |
| *NET1*_Enhancer-1 | GAGGAATGTGCAAACGGCCC | chr10:5,493,900-5,493,919 | Zhang et al., Nat Comm., 2019 |
| *NET1*_Enhancer-2 | AGCCTACACCCTGGAGGTAC | chr10:5,493,772-5,493,791 | Zhang et al., Nat Comm., 2019 |
| *TFF1*_Enhancer-1 | CACTTGTTTTATCCACCAGG | chr21:42,367,559-42,367,578 | This Study |
| *TFF1*_Enhancer-2 | GATTAAGTCTCCTTCTGGGA | chr21:42,367,305-42,367,324 | This Study |
| *TFF1*_Enhancer-3 | TCATGAAGCCCATTCCGTCT | chr21:42,366,963-42,366,982 | This Study |
| *GREB1*_Enhancer-1 | GGGACAAGCACACACATCGC | chr2:11,532,725-11,532,744 | This Study |
| *GREB1*_Enhancer-2 | TGTGAATGGTTTCCTTGATC | chr2:11,532,374-11,532,393 | This Study |
| *FKBP5*_Enhancer-1 | GGTGCTCCAAGGCCGGTGAG | chr6:35,727,917-35,727,936 | This Study |
| *FKBP5*_Enhancer-2 | CTGGAACACAATGTCCCGAG | chr6:35,728,111-35,728,130 | This Study |
| *FKBP5*_Enhancer-3 | GAACGGGAGGGGCAAGCATA | chr6:35,728,264-35,728,283 | This Study |
| *FKBP5*_Enhancer-4 | TGGGAGACGCTTTAATAGAG | chr6:35,728,367-35,728,386 | This Study |
| GRASLND_Promoter-1 | CCACTGGGGATAGTTCCCTG | chr2:6,918,864-6,918,883 | Huynh et al., eLife, 2020 |
| GRASLND_Promoter-2 | TGACACCGTGAACTTTCCCA | chr2:6,919,268-6,919,287 | This Study |
| GRASLND_Promoter-3 | TAAGTTGGTGTAGACGCCTG | chr2:6,919,064-6,919,083 | This Study |
| GRASLND_Promoter-4 | TGAATTCTAGGCCAAGACTT | chr2:6,919,475-6,919,494 | This Study |
| MALAT1_Promoter-1 | CCAACTTCCATTTTCAGTCT | chr11:65,497,346-65,497,365 | This Study |
| MALAT1_Promoter-2 | ACGAGCCAGCGCCTCACAAA | chr11:65,497,594-65,497,613 | This Study |
| MALAT1_Promoter-3 | CCTGTTCTGGGGAATGGCAT | chr11:65,497,461-65,497,480 | This Study |
| MALAT1_Promoter-4 | GGGCACACAAAGCTTGGAGC | chr11:65,497,063-65,497,082 | This Study |
| HOTAIR_Promoter-1 | TGTATATCAACAAAGAGAAG | chr12:53,975,262-53,975,281 | This Study |
| HOTAIR_Promoter-2 | GCCACAGTCCAGTTTTCCAC | chr12:53,975,507-53,975,526 | This Study |
| HOTAIR_Promoter-3 | CCCTTCTCCTAGCCCACCGC | chr12:53,975,072-53,975,091 | This Study |
| miR-146a_Promoter-1 | CTAGAAGGGACTTTCCAGAG | chr5:160,468,125-160,468,144 | This Study |
| miR-146a_Promoter-2 | CTCCTGACACGTGCTGCAAG | chr5:160,467,954-160,467,973 | This Study |
| miR-146a_Promoter-3 | GCCTGACCAGAACTTCCTCG | chr5:160,467,862-160,467,881 | This Study |
| miR-146a_Promoter-4 | CTGCCTGATCTTCTCCCCAA | chr5:160,467,648-160,467,667 | This Study |
| CCAT1_Promoter-1 | GATTTACGTGGCAATTACCA | chr8:127,219,316-127,219,335 | This Study |
| CCAT1_Promoter-2 | GTTGCAAGATCTTAGTGTGA | chr8:127,219,413-127,219,432 | This Study |
| CCAT1_Promoter-3 | CATTCTGGTTGCCAAGGAAA | chr8:127,219,575-127,219,594 | This Study |
| CCAT1_Promoter-4 | AAGGACTTCTTCATGTACCC | chr8:127,219,750-127,219,769 | This Study |
| *TMPRSS2*_Promoter-1 | TGAGATTAAAGCGAGAGCCA | chr21:41,508,173-41,508,192 | This Study |
| *TMPRSS2*_Promoter-2 | TTAACCCAAACTCGAGCCCT | chr21:41,508,426-41,508,445 | This Study |
| *TMPRSS2*_Promoter-3 | ACCTCACGAGCTCCTGTAGG | chr21:41,508,501-41,508,520 | This Study |
| *TMPRSS2*_Promoter-4 | CTGGCCCAAGCACGAGTGGC | chr21:41,508,597-41,508,616 | This Study |
| *UPK3B*_Promoter-1 | TACTCCCCACTCCTGACTCT | chr7:76,510,215-76,510,234 | This Study |
| *ITIH2*_Promoter-1 | ATTGGGACAAATATTTGTTC | chr10:7,703,261-7,703,280 | This Study |

| | | | |
|---|---|---|---|
| *HNF1A*_Promoter-1 | ACACGGATAAATATGAACCT | chr12:120,978,509-120,978,528 | This Study |
| *STAR*_Promoter-1 | GAAGGCTGTGCATCATCATC | chr8:38,150,993-38,151,012 | This Study |
| *CYP11A1*_Promoter-1 | GTGAGGTTCTCCAAAGGACA | chr15:74,367,802-74,367,821 | This Study |
| *HSD3B2*_Promoter-1 | GATTGCAGATCCCAGACAGC | chr1:119,414,885-119,414,904 | This Study |
| *HSD3B2*_Promoter-2 | CTTGGAGACTTCTCCCAGTT | chr1:119,414,850-119,414,869 | This Study |
| *MEF2C*_Promoter-1 | GAAGACGGAGCACGAATGGT | chr5:88,883,557-88,883,576 | Wang et al., Acta Pharm Sin B, 2019 |
| *GATA4*_Promoter-1 | CGCCCAGCGGAGGTGTAGCC | chr8:11,704,084-11,704,103 | Wang et al., Acta Pharm Sin B, 2019 |
| *MEIS1*_Promoter-1 | CTTGCAAAGAGGGAGAGAGA | chr2:66,435,279-66,435,298 | Wang et al., Acta Pharm Sin B, 2019 |
| *Pparg2* (Mouse) | GAATAAACACAGAAAGAATC | chr6:115,398,896-115,398,915 | Lundh et al., Mol Metab, 2017 |
| *Ucp1* (Mouse) | GGGAGTGACGCGCGGCTGGG | chr8:84,016,825-84,016,844 | Lundh et al., Mol Metab, 2017 |
| *St6gal1*_Promoter-1 (Chinese Hamster) | ATGGTGCATAGACTAAGCG | Not annotated | Karottki et al., Biotech & Bioeng, 2019 |
| *St6gal1*_Promoter-2 (Chinese Hamster) | CAGAGGACTTCCGAGAATC | Not annotated | Karottki et al., Biotech & Bioeng, 2019 |
| *Mgat3*_Promoter-1 (Chinese Hamster) | GGTGTGGTTACCACGCCCA | Not annotated | Karottki et al., Biotech & Bioeng, 2019 |
| *Mgat3*_Promoter-2 (Chinese Hamster) | GTGATGGGGTCTCGGGTAT | Not annotated | Karottki et al., Biotech & Bioeng, 2019 |
| *Mgat3*_Promoter-3 (Chinese Hamster) | ACAGGTCAGGGCAAGCCGG | Not annotated | Karottki et al., Biotech & Bioeng, 2019 |
| *Agrp*_Promoter-1 (Mouse) | CCTGACAACACAGGGCGGAA | chr8:106,294,961-106,294,980 | This Study |
| *Agrp*_Promoter-2 (Mouse) | AAGCTGATGAGGCCAGGCGT | chr8:106,294,990-106,295,009 | This Study |
| *Agrp* _Promoter-3 (Mouse) | CTTACTCTGTCAAGCTGATG | chr8:106,295,001-106,295,020 | This Study |
| *Agrp*_Promoter-4 (Mouse) | GAGTAAGGACTGCACATTTG | chr8:106,295,001-106,295,020 | This Study |
| *Agrp*_Promoter-5 (Mouse) | AGTAAGGACTGCACATTTGT | chr8:106,295,015-106,295,034 | This Study |
| *Agrp*_Promoter-6 (Mouse) | GTGGGTGTAACAGGACCCAT | chr8:106,295,033-106,295,052 | This Study |
| *Agrp*_Promoter-7 (Mouse) | ATTTGCCATGACACTTGGTG | chr8:106,295,167-106,295,186 | This Study |
| *Agrp*_Promoter-8 (Mouse) | TGCATGGCCAGTACCCACGA | chr8:106,295,254-106,295,273 | This Study |
| *Agrp*_Promoter-9 (Mouse) | GGGATATACCCCAGTCCACA | chr8:106,295,461-106,295,480 | This Study |
| *Agrp*_Promoter-10 (Mouse) | TAGAGTCCACATCAATGTTT | chr8:106,295,614-106,295,633 | This Study |
| *Agrp*_Promoter-11 (Mouse) | TGTTTGGGTTCAAGCTTCAC | chr8:106,295,629-106,295,648 | This Study |
| *Agrp*_Promoter-12 (Mouse) | CTGTATCCCGAGTCACCTGC | chr8:106,295,726-106,295,745 | This Study |
| *Agrp*_Promoter-13 (Mouse) | AACTGCCTATACCATTCTTA | chr8:106,295,875-106,295,894 | This Study |
| *Agrp*_Promoter-14 (Mouse) | GAGGCTGTCATTGACCTGTT | chr8:106,295,899-106,295,918 | This Study |
| *Agrp*_Promoter-15 (Mouse) | GACCTGTTTGGAAGCTGATC | chr8:106,295,911-106,295,930 | This Study |

## Supplementary Table 2: *Staphylococcus aureus* Cas9 Protospacer sequences used in this study.

| Target Gene | Protospacer Sequence (SaCas9) (5'-3') | Genomic Location (GRCh38/hg38 Assembly) | Reference |
|---|---|---|---|
| *HBG1*_Promoter-1 | GGCCGGCGGCTGGCTAGGGATG | chr11:5,249,892-5,249,913 | This Study |
| *HBG1*_Promoter-2 | AGGCAAACTTGACCAATAGTCT | chr11:5,254,860-5,254,881 | This Study |
| *HBG1*_Promoter-3 | CTAGTTTCCTTCTCCCATCATA | chr11:5,250,153-5,250,174 | This Study |
| *HBG1*_Promoter-4 | GAAATGTGTTTTAGGCATAGGTC | chr11:5,255,218-5,255,240 | This Study |
| *TTN*_Promoter-1 | TGTGGGGCTGTCAGCAGATCTG | chr2:178,807,457-178,807,478 | This Study |
| *TTN*_Promoter-2 | CAAGGAAATAGAACTGTATTTA | chr2:178,807,611-178,807,632 | This Study |
| *TTN*_Promoter-3 | CCCCAAACCTGAGGACTGTGCC | chr2:178,807,611-178,807,632 | This Study |
| *TTN*_Promoter-4 | CCCGAAATACAAGTGGATTCTG | chr2:178,807,946-178,807,967 | This Study |
| *Agrp*_Promoter-1 (Mouse) | AAATGTGCAGTCCTTACTCTG | chr8:105,568,380-105,568,400 | This Study |
| *Agrp*_Promoter-2 (Mouse) | TCAAGCTGATGAGGCCAGGCG | chr8:105,568,359-105,568,379 | This Study |
| *Agrp*_Promoter-3 (Mouse) | GTAGGCAGAGACCTGACAACA | chr8:105,568,339-105,568,359 | This Study |

## Supplementary Table 3: *Campylobacter jejuni* Cas9 Protospacer sequences used in this study.

| Target Gene | Protospacer Sequence (CjCas9) (5'-3') | Genomic Location (GRCh38/hg38 Assembly) | Reference |
|---|---|---|---|
| *HBG1*_Promoter-1 | CTAGTTTCCTTCTCCCATCATA | chr11:5,250,153-5,250,174 | Zhang et al., Nucleic Acid Res., 2021 |
| *HBG1*_Promoter-2 | TCAAAAATCCTGGACCTATGCC | chr11:5,255,231-5,255,252 | Zhang et al., Nucleic Acid Res., 2021 |
| *HBG1*_Promoter-3 | AGTATCCTCTTGGGGGCCCCTT | chr11:5,254,979-5,255,000 | Zhang et al., Nucleic Acid Res., 2021 |
| *TTN*_Promoter-1 | TCAATCAGAAGAACAGGTGGTA | chr2:178,807,554-178,807,575 | This Study |
| *TTN*_Promoter-2 | CAACCAATGCTCTTCTCTGAGC | chr2:178,807,651-178,807,672 | This Study |
| *TTN*_Promoter-3 | ACTGCCCACAAGTGCTATCTAC | chr2:178,807,716-178,807,737 | This Study |

## Supplementary Table 4: *Acidaminococcus sp.* Cas12a crRNA sequences used in this study.

| Target Gene | crRNA Spacer Sequence (dAsCas12a) (5'-3') | Genomic Location (GRCh38/hg38 Assembly) | Reference |
|---|---|---|---|
| *ASCL1*_crRNA1 | GGGAGTGGGTGGGAGGAAGA | chr12:102,957,523-102,957,542 | Campa et al., Nat Methods, 2019 |
| *ASCL1*_crRNA2 | CAATGGGACACCCAGCCCCA | chr12:102,957,628-102,957,647 | Campa et al., Nat Methods, 2019 |
| *IL1R2*_crRNA1 | CTTGGCCACTTCCCCATCTG | chr2:101,991,780-101,991,799 | Campa et al., Nat Methods, 2019 |
| *IL1R2*_crRNA2 | CTAACCGGGTGGTGTCACCC | chr2:101,991,888-101,991,907 | This Study |
| *IL1B*_crRNA1 | CATGGTGATACATTTGCAAA | chr2:112,836,988-112,837,007 | Campa et al., Nat Methods, 2019 |
| *IL1B*_crRNA2 | CTACTCCTTGCCCTTCCATG | chr2:112,836,953-112,836,972 | Campa et al., Nat Methods, 2019 |
| *ZFP42*_crRNA1 | TTGAGCGCTCACCACGTGCC | chr4:187,995,398-187,995,417 | Campa et al., Nat Methods, 2019 |
| *ZFP42*_crRNA2 | CGTGCGGGCCGGGTGCCTGG | chr4:187,995,529-187,995,548 | Campa et al., Nat Methods, 2019 |
| *SBNO2*_crRNA1 | AGGAAATGATGCCGGCCCCG | chr19:1,132,328-1,132,347 | This Study |
| GRASLND-crRNA1 | CAGGGACACCCTGACTCCCT | chr2:6,919,115-6,919,134 | This Study |
| *SOCS1* +15KB-crRNA1 | TTAGAAATCAGCCACCTGGG | chr16:11,240,841-11,240,860 | This Study |
| NET1eRNA-crRNA1 | TCTAGGTAAACAGCAGTTTC | chr10:5,493,860-5,493,879 | This Study |
| *OCT4*-crRNA1 | CCCTTCCACAGACACCATTG | chr6:31,170,808-31,170,827 | This Study |
| *HBG1*-crRNA1 | TTCTTCATCCCTAGCCAGCC | chr11:5,254,811-5,254,830 | This Study |
| *CARD9*-crRNA1 | CTCTGGAGGGCAGCGGACTT | chr9:136,373,771-136,373,790 | This Study |
| *IL1RN*-crRNA1 | TGCTAGCCTGAGTCACCCTC | chr2:113,117,740-113,117,759 | This Study |
| *LELP1*-crRNA1 | ACCAGCCTGAATGTTGAGGG | chr1:153,203,239-153,203,258 | This Study |
| *TRAT1*-crRNA1 | TAACTGAAACAAAGTACACA | chr3:108,822,729-108,822,748 | This Study |
| *TTN*-crRNA1 | AGGTACTAAATTTAGCACTG | chr2:178,807,576-178,807,595 | This Study |
| *TTN*-crRNA2 (15bp) | CTTGGTGAAGTCTCC | chr2:178,807,600-178,807,614 | Breinig et al., Nat Methods, 2018 |
| *TTN*-crRNA3 (15bp) | GCACTGTCAATCAGA | chr2:178,807,567-178,807,581 | Breinig et al., Nat Methods, 2018 |
| *TTN*-crRNA4 (15bp) | TGGGGGAAGGGAACA | chr2:178,807,791-178,807,805 | Breinig et al., Nat Methods, 2018 |
| *TBX5*-crRNA1 | TCTCCGCAAGGCACATTACG | chr12:114,408,626-114,408,645 | This Study |

## Supplementary Table 5: *Escherichia coli* Type I CRISPR crRNA sequences used in this study.

| Target Gene | crRNA Spacer Sequence (Eco-Cascade) (5'-3') | Genomic Location (GRCh38/hg38 Assembly) | Reference |
|---|---|---|---|
| *HBG1*-crRNA | GGTGCTTCCTTTTATTCTTCATCCCTAGCC | chr11:5,254,797-5,254,826 | Pickar-Oliver et al., Nat Biotech, 2019 |
| *IL1RN*-crRNA | GAGGGGCAGCTCCACCCTGGGAGGGACTGTTC | chr2:113,117,890-113,117,919 | Pickar-Oliver et al., Nat Biotech, 2019 |
| *TTN*-crRNA | TCTCCTTTGAGGTACTAAATTTAGCACTGT | chr2:178,807,575-178,807,603 | This study |
| *SBNO2*-crRNA | TCAGCCCCGGCCTTAGAGGAAGTAGGCGCC | chr19:1,132,293-1,132,322 | This Study |
| *OCT4*-crRNA | GGGAAAACCGGGAGACACAACTGGCGCCCC | chr19:1,132,291-1,132,322 | This Study |
| *CD34*-crRNA | AGCGCGTCCTGGCCAAGCCGAGTAGTGTCT | chr1:207,911,119-207,911,148 | This Study |
| NET1eRNA-crRNA | GTCATCCTCTAACCCTGGGCCGTTTGCACA | chr10:5,493,906-5,493,935 | This Study |

## Supplementary Table 6: *Pseudomonas aeruginosa* Type I CRISPR crRNA sequences used in this study.

| Target Gene | crRNA Spacer Sequence (Pae-Cascade) (5'-3') | Genomic Location (GRCh38/hg38 Assembly) | Reference |
|---|---|---|---|
| *HBG1*-crRNA | ACACTATCTCAATGCAAATATCTGTCTGAAAC | chr11:5,250,019-5,250,050 | Chen et al., Nat Comm, 2020 |
| *IL1B*-crRNA1 | ATGAACCAGAGAATTATCTCAGTTTATTAGTC | chr12:31,532,095-31,532,127 | Chen et al., Nat Comm, 2020 |

## Supplementary Table 7: QPCR Primer Sequences.

| Target | Forward Primer (5'-3') | Reverse Primer (5'-3') | Cycling Parameters | |
|---|---|---|---|---|
| *GAPDH* | CAATGACCCCTTCATTGACC | TTGATTTTGGAGGGATCTCG | 95˚C 30 sec<br>95˚C 10 sec<br>60˚C 30 sec | 40X-45X |
| *OCT4* | CGAAAGAGAAAGCGAACCAGTATC GAGAAC | CGTTGTGCATAGTCGCTGCTTGAT CGC | 95˚C 30 sec<br>95˚C 10 sec<br>60˚C 30 sec | 40X-45X |
| *IL1RN* | GGAATCCATGGAGGGAAGAT | TGTTCTCGCTCAGGTCAGTG | 95˚C 30 sec<br>95˚C 10 sec<br>60˚C 30 sec | 40X-45X |
| *TTN* | TGTTGCCACTGGTGCTAAAG | ACAGCAGTCTTCTCCGCTTC | 95˚C 30 sec<br>95˚C 10 sec<br>60˚C 30 sec | 40X-45X |
| *HBG1* | GGAAGATGCTGGAGGAGAAACC | GTCAGCACCTTCTTGCCATGTG | 95˚C 30 sec<br>95˚C 10 sec<br>60˚C 30 sec | 40X-45X |
| *HBE* | TCACTAGCAAGCTCTCAGGC | AACAACGAGGAGTCTGCCC | 95˚C 30 sec<br>95˚C 10 sec<br>60˚C 30 sec | 40X-45X |
| *HBD* | GCACGTGGATCCTGAGAACT | CAGGAAACAGTCCAGGATCTCA | 95˚C 30 sec<br>95˚C 10 sec<br>60˚C 30 sec | 40X-45X |
| TRK-A | CCAGTGACCTCAACAGGAAG | GTTGACCTGAACAGAGACCTC | 95˚C 30 sec<br>95˚C 10 sec<br>60˚C 30 sec | 40X-45X |
| *TRK-B* | GAATATGGACCCTGAAGCCTG | CAAGGGACTTGACCTGTGTAC | 95˚C 30 sec<br>95˚C 10 sec<br>60˚C 30 sec | 40X-45X |
| *TRK-C* | CCATCAAGAACTCAGGACTTCG | TGCAATTCCCGAAGACTCAG | 95˚C 30 sec<br>95˚C 10 sec<br>60˚C 30 sec | 40X-45X |
| *CD34* | AATAGCCAGTGATGCCCAAG | GGTATGCTCCCTGCTCCTT | 95˚C 30 sec<br>95˚C 10 sec<br>60˚C 30 sec | 40X-45X |
| *NEUDO-D* | GGATGACGATCAAAAGCCCAA | GCGTCTTAGAATAGCAAGGCA | 95˚C 30 sec<br>95˚C 10 sec<br>60˚C 30 sec | 40X-45X |
| *ASCL1* | GGAGCTTCTCGACTTCACCA | AACGCCACTGACAAGAAAGC | 95˚C 30 sec<br>95˚C 10 sec<br>60˚C 30 sec | 40X-45X |
| *HER2* | GGGAAACCTGGAACTCACCT | GACCTGCCTCACTTGGTTGT | 95˚C 30 sec<br>95˚C 10 sec<br>60˚C 30 sec | 40X-45X |
| *CARD9* | CAGGCTCCTGGTGTGTCTG | CTCCAGCACTCGTCATCGT | 95˚C 30 sec<br>95˚C 10 sec<br>60˚C 30 sec | 40X-45X |
| *KDM2B* | CATGCAGCAGAAAAGCAAAA | TCCGACAAGTCCTCGTTCTC | 95˚C 30 sec<br>95˚C 10 sec<br>60˚C 30 sec | 40X-45X |
| *RAB19* | GCTGAAAAGCAAACCCAGAA | AGCTGGAGAAGTGCATGGTT | 95˚C 30 sec<br>95˚C 10 sec<br>60˚C 30 sec | 40X-45X |

| | | | | |
|---|---|---|---|---|
| *SBNO2* | GCAGTTTGAGGCTCTGAACA | ATGGTGGACACCTGGTTGAG | 95˚C 30 sec<br>95˚C 10 sec<br>60˚C 30 sec | 40X-45X |
| *SPARC* | GGTTTCCTGTTGCCTGTCTC | AAGAAGATCCAGGCCCTCAT | 95˚C 30 sec<br>95˚C 10 sec<br>60˚C 30 sec | 40X-45X |
| *HGF* | CATGTCCTCCTGCATCTCCT | TGCTGGATCTATTTTGATTAGGG | 95˚C 30 sec<br>95˚C 10 sec<br>60˚C 30 sec | 40X-45X |
| *TBX5* | ACCCAGCATAGGAGCTGGC | ACACTCAGCCTCACATCTTAC | 95˚C 30 sec<br>95˚C 10 sec<br>60˚C 30 sec | 40X-45X |
| *SOCS1* | CAGCTTAACTGTATCTGGAGCC | AAATAAAGCCAGAGACCCTCC | 95˚C 30 sec<br>95˚C 10 sec<br>60˚C 30 sec | 40X-45X |
| *FOXA3* | GCTCAGTGAAGATGGAGGC | GCTTAGAGGATTCAGGGTCATG | 95˚C 30 sec<br>95˚C 10 sec<br>60˚C 30 sec | 40X-45X |
| NET1-eRNA | CAGGCCTACCAGGATGGATA | AGTTGACTTGGGTGGGACAG | 95˚C 30 sec<br>95˚C 10 sec<br>60˚C 30 sec | 40X-45X |
| KLK3-eRNA-Sense | AGGGTATCACCAGCCCTTCT | GAGGATGTCGGCAGCTCTAC | 95˚C 30 sec<br>95˚C 10 sec<br>60˚C 30 sec | 40X-45X |
| KLK3-eRNA-Antisense | GACGATCAAATGTGGTCACG | CTCCATCAAATGAGGCCAGT | 95˚C 30 sec<br>95˚C 10 sec<br>60˚C 30 sec | 40X-45X |
| GRASLND | CTTTAGTGGCCTCCAGAAGAC | GCCCTGGTTTACAGCGTT | 95˚C 30 sec<br>95˚C 10 sec<br>60˚C 30 sec | 40X-45X |
| MALAT1 | CAGACCCAGAGCAGTGTAAAC | CATGGAAAGCGAGTTCAAGTG | 95˚C 30 sec<br>95˚C 10 sec<br>60˚C 30 sec | 40X-45X |
| HOTAIR | CAGTGGGGAACTCTGACTCG | GTGCCTGGTGCTCTCTTACC | 95˚C 30 sec<br>95˚C 10 sec<br>60˚C 30 sec | 40X-45X |
| *TRAT1* | ACAGCTACTCCAGTGACCACAC | CAGATTTCTCTGGTCGGGCTTTC | 95˚C 30 sec<br>95˚C 10 sec<br>60˚C 30 sec | 40X-45X |
| *IL1B* | AAACAGATGAAGTGCTCCTTCC | AAGATGAAGGGAAAGAAGGTGC | 95˚C 30 sec<br>95˚C 10 sec<br>60˚C 30 sec | 40X-45X |
| *IL1R2* | ATGTTGCGCTTGTACGTGTTG | CCCGCTTGTAATGCCTCCC | 95˚C 30 sec<br>95˚C 10 sec<br>60˚C 30 sec | 40X-45X |
| *ZFP42* | AGAAACGGGCAAAGACAAGAC | GCTGACAGGTTCTATTTCCGC | 95˚C 30 sec<br>95˚C 10 sec<br>60˚C 30 sec | 40X-45X |
| *TRAT1* | ACAGCTACTCCAGTGACCACAC | CAGATTTCTCTGGTCGGGCTTTC | 95˚C 30 sec<br>95˚C 10 sec<br>60˚C 30 sec | 40X-45X |
| *MYOD* | TCCCTCTTTCACGGTCTCAC | AACACCCGACTGCTGTATCC | 95˚C 30 sec<br>95˚C 10 sec<br>60˚C 30 sec | 40X-45X |
| *RHOXF2* | GATTGCAATGCCTGCAGATAAG | AGTTCATGCTCACCCAAGAAA | 95˚C 30 sec<br>95˚C 10 sec<br>60˚C 30 sec | 40X-45X |
| *ACE2* | TCTCACAGTCAAGCTTCAGC | GTTCAACCGTTTGCTCTTGTC | 95˚C 30 sec<br>95˚C 10 sec<br>60˚C 30 sec | 40X-45X |
| *CD25* | AGGGATACAGGGCTCTACAC | TGGTCTCCATTTCACCTGTG | 95˚C 30 sec<br>95˚C 10 sec<br>60˚C 30 sec | 40X-45X |
| TFF1-eRNA Sense | GGATTAAGGTCAGGTTGGAGGA | ACGACATGTGGTGAGGTCAT | 95˚C 30 sec<br>95˚C 10 sec<br>60˚C 30 sec | 40X-45X |
| TFF1-eRNA Antisense | TGAGAAATTAGGCCCTGAGGA | AGAGGGGCTGCAGAAATGTA | 95˚C 30 sec<br>95˚C 10 sec<br>60˚C 30 sec | 40X-45X |

| | | | | |
|---|---|---|---|---|
| *GREB1-eRNA*-Sense | AGCCAGTAGATACCAGGCAC | CTCCCATTAGTTTGGAGTTGCC | 95˚C 30 sec<br>95˚C 10 sec<br>60˚C 30 sec | 40X-45X |
| *GREB1-eRNA*-Antisense | GCATATTCAAATGACAGAAAGTACG | ATGTGAATGATACCGCTTTCTCT | 95˚C 30 sec<br>95˚C 10 sec<br>60˚C 30 sec | 40X-45X |
| CCAT1 | CTTTGAAGTTGCACTGACCTG | CTCACAGTTTTCAAGGGATTTTAGG | 95˚C 30 sec<br>95˚C 10 sec<br>60˚C 30 sec | 40X-45X |
| *TMPRSS2* | CCTCTAACTGGTGTGATGGCGT | TGCCAGGACTTCCTCTGAGATG | 95˚C 30 sec<br>95˚C 10 sec<br>60˚C 30 sec | 40X-45X |
| *UPK3B* | TGTGTCTTCGATGGGCTTGCCA | CGGAATGTCAGCCAGTGTCTCC | 95˚C 30 sec<br>95˚C 10 sec<br>60˚C 30 sec | 40X-45X |
| *STAR* | TACGTGGCTACTCAGCATCGAC | TCAACACCTGGCTTCAGAGGCA | 95˚C 30 sec<br>95˚C 10 sec<br>60˚C 30 sec | 40X-45X |
| *CYP11A1* | TGGCATCCTCTACAGACTCCTG | CTTCAGGTTGCGTGCCATCTCA | 95˚C 30 sec<br>95˚C 10 sec<br>60˚C 30 sec | 40X-45X |
| *HSD3B2* | CGCCTGTATCATTGATGTCTTTGG | CTGGTGTAGATGAAGACTGGCAC | 95˚C 30 sec<br>95˚C 10 sec<br>60˚C 30 sec | 40X-45X |
| *MEF2C* | ACGTAACAGACAGGTGACAT | CGGCTCGTTGTACTCCGTG | 95˚C 30 sec<br>95˚C 10 sec<br>60˚C 30 sec | 40X-45X |
| *GATA4* | CCTCTCCTGTGCCAACTG | ATCCCCTCTTTCCGCATTG | 95˚C 30 sec<br>95˚C 10 sec<br>60˚C 30 sec | 40X-45X |
| *MEIS1* | GGCACAAGACACGGGACTCA | CATGGGCTGTCCATCAGGATTA | 95˚C 30 sec<br>95˚C 10 sec<br>60˚C 30 sec | 40X-45X |
| *ITIH2* | AATTCTACAACCAGGTCTCCAC | ACAATCTCTGAGCCTCCAAAG | 95˚C 30 sec<br>95˚C 10 sec<br>60˚C 30 sec | 40X-45X |
| *SOX2* | GCCCTGCAGTACAACTCCAT | TGCCCTGCTGCGAGTAGGA | 95˚C 30 sec<br>95˚C 10 sec<br>60˚C 30 sec | 40X-45X |
| *NANOG* | CTCAGCCTCCAGCAGATGC | TAGATTTCATTCTCTGGTTCTGG | 95˚C 30 sec<br>95˚C 10 sec<br>60˚C 30 sec | 40X-45X |
| *LIN28A* | AGGAGACAGGTGCTACAACTG | TCTTGGGCTGGGGTGGCAG | 95˚C 30 sec<br>95˚C 10 sec<br>60˚C 30 sec | 40X-45X |
| *ZFP42/REX1* (used in iPSC QPCR) | CGTTTCGTGTGTCCCTTTCAA | CCTCTTGTTCATTCTTGTTCGT | 95˚C 30 sec<br>95˚C 10 sec<br>60˚C 30 sec | 40X-45X |
| *CDH1* | GAGACAGTTTCGCTCCATCG | AGCTTGGGCAACATAGCAAG | 95˚C 30 sec<br>95˚C 10 sec<br>60˚C 30 sec | 40X-45X |
| *FGF4* | ACCTTGGTGCACTTTCTTCG | AAAAAACACACCCGCAGAAC | 95˚C 30 sec<br>95˚C 10 sec<br>60˚C 30 sec | 40X-45X |
| *THY1* | GAAGGTCCTCTACTTATCCGCC | TGATGCCCTCACACTTGACCAG | 95˚C 30 sec<br>95˚C 10 sec<br>60˚C 30 sec | 40X-45X |
| *ZEB1* | GCTAAGAACTGCTGGGAGGAT | ATCCTGCTTCATCTGCCTGA | 95˚C 30 sec<br>95˚C 10 sec<br>60˚C 30 sec | 40X-45X |
| *ZEB2* | AAGCCAGGGACAGATCAGC | CCACACTCTGTGCATTTGAACT | 95˚C 30 sec<br>95˚C 10 sec<br>60˚C 30 sec | 40X-45X |
| *TWIST1* | AAGGCATCACTATGGACTTTCTCT | GCCAGTTTGATCCCAGTATTTT | 95˚C 30 sec<br>95˚C 10 sec<br>60˚C 30 sec | 40X-45X |
| *SNAIL2* | TGGTTGCTTCAAGGACACAT | GTTGCAGTGAGGGCAAGAA | 95˚C 30 sec<br>95˚C 10 sec<br>60˚C 30 sec | 40X-45X |

| | | | | |
|---|---|---|---|---|
| Pre-miR-146a | TGAGAACTGAATTCCATGGGTT | ATCTACTCTCTCCAGGTCCTCA | 95˚C 30 sec<br>95˚C 10 sec<br>60˚C 30 sec | 40X-45X |
| Mature miR-146a | TGAGAACTGAATTCCATGGGTT | PerfeCTa Universal PCR primer (Quantabio) | 95˚C 30 sec<br>95˚C 10 sec<br>60˚C 30 sec | 40X-45X |
| U6 snRNA | CGCTTCGGCAGCACATATAC | PerfeCTa Universal PCR primer (Quantabio) | 95˚C 30 sec<br>95˚C 10 sec<br>60˚C 30 sec | 40X-45X |
| *Agrp* (Mouse) | GCCTCAAGAAGACAACTGCAGAC | AAGCAGGACTCGTGCAGCCTTA | 95˚C 30 sec<br>95˚C 10 sec<br>60˚C 30 sec | 40X-45X |

## Supplementary Table 8: QPCR Primer used in CUT&RUN.

| Target | Forward Primer (5’-3’) | Reverse Primer (5’-3’) | Cycling Parameters | |
|---|---|---|---|---|
| *HBG1* | CTTCAGCAGTTCCACACACT | GTCCTTCCTTCCCTCCCT | 95˚C 30 sec<br>95˚C 10 sec<br>60˚C 30 sec | 40X-45X |

**Supplementary Note 7.** Amino acid sequences of selected constructs in this study.

**SpdCas9**: amino acid sequence; Streptococcus pyogenes Cas9 (D10A, H840A), Nucleoplasmin Nuclear Localization Sequence, Glysine-Serine Linker Sequence, SV40 Nuclear Localization Sequence, 1 X "Flag" Epitope

MDKKYSIGLAIGTNSVGWAVITDEYKVPSKKFKVLGNTDRHSIKKNLIGALLFDSGETAEATRLKRTARRR
YTRRKNRICYLQEIFSNEMAKVDDSFFHRLEESFLVEEDKKHERHPIFGNIVDEVAYHEKYPTIYHLRKKLV
DSTDKADLRLIYLALAHMIKFRGHFLIEGDLNPDNSDVDKLFIQLVQTYNQLFEENPINASGVDAKAILSARL
SKSRRLENLIAQLPGEKKNGLFGNLIALSLGLTPNFKSNFDLAEDAKLQLSKDTYDDDLDNLLAQIGDQYA
DLFLAAKNLSDAILLSDILRVNTEITKAPLSASMIKRYDEHHQDLTLLKALVRQQLPEKYKEIFFDQSKNGYA
GYIDGGASQEEFYKFIKPILEKMDGTEELLVKLNREDLLRKQRTFDNGSIPHQIHLGELHAILRRQEDFYPF
LKDNREKIEKILTFRIPYYVGPLARGNSRFAWMTRKSEETITPWNFEEVVDKGASAQSFIERMTNFDKNLP
NEKVLPKHSLLYEYFTVYNELTKVKYVTEGMRKPAFLSGEQKKAIVDLLFKTNRKVTVKQLKEDYFKKIEC
FDSVEISGVEDRFNASLGTYHDLLKIIKDKDFLDNEENEDILEDIVLTLTLFEDREMIEERLKTYAHLFDDKV
MKQLKRRRYTGWGRLSRKLINGIRDKQSGKTILDFLKSDGFANRNFMQLIHDDSLTFKEDIQKAQVSGQG
DSLHEHIANLAGSPAIKKGILQTVKVVDELVKVMGRHKPENIVIEMARENQTTQKGQKNSRERMKRIEEGI
KELGSQILKEHPVENTQLQNEKLYLYYLQNGRDMYVDQELDINRLSDYDVDAIVPQSFLKDDSIDNKVLTR
SDKNRGKSDNVPSEEVVKKMKNYWRQLLNAKLITQRKFDNLTKAERGGLSELDKAGFIKRQLVETRQITK
HVAQILDSRMNTKYDENDKLIREVKVITLKSKLVSDFRKDFQFYKVREINNYHHAHDAYLNAVVGTALIKKY
PKLESEFVYGDYKVYDVRKMIAKSEQEIGKATAKYFFYSNIMNFFKTEITLANGEIRKRPLIETNGETGEIV
WDKGRDFATVRKVLSMPQVNIVKKTEVQTGGFSKESILPKRNSDKLIARKKDWDPKKYGGFDSPTVAYS
VLVVAKVEKGKSKKLKSVKELLGITIMERSSFEKNPIDFLEAKGYKEVKKDLIIKLPKYSLFELENGRKRMLA
SAGELQKGNELALPSKYVNFLYLASHYEKLKGSPEDNEQKQLFVEQHKHYLDEIIEQISEFSKRVILADANL
DKVLSAYNKHRDKPIREQAENIIHLFTLTNLGAPAAFKYFDTTIDRKRYTSTKEVLDATLIHQSITGLYETRID
LSQLGGDKRPAATKKAGQAKKKKSGGGSGGSGSGSPKKKRKVDYKDDDDK

**SpdCas9-VP64**: amino acid sequence; Streptococcus pyogenes Cas9 (D10A, H840A), Nucleoplasmin Nuclear Localization Sequence, Glysine-Serine Linker Sequence, VP64, SV40 Nuclear Localization Sequence,1 X "Flag" Epitope

MDKKYSIGLAIGTNSVGWAVITDEYKVPSKKFKVLGNTDRHSIKKNLIGALLFDSGETAEATRLKRTARRR
YTRRKNRICYLQEIFSNEMAKVDDSFFHRLEESFLVEEDKKHERHPIFGNIVDEVAYHEKYPTIYHLRKKLV
DSTDKADLRLIYLALAHMIKFRGHFLIEGDLNPDNSDVDKLFIQLVQTYNQLFEENPINASGVDAKAILSARL
SKSRRLENLIAQLPGEKKNGLFGNLIALSLGLTPNFKSNFDLAEDAKLQLSKDTYDDDLDNLLAQIGDQYA
DLFLAAKNLSDAILLSDILRVNTEITKAPLSASMIKRYDEHHQDLTLLKALVRQQLPEKYKEIFFDQSKNGYA
GYIDGGASQEEFYKFIKPILEKMDGTEELLVKLNREDLLRKQRTFDNGSIPHQIHLGELHAILRRQEDFYPF
LKDNREKIEKILTFRIPYYVGPLARGNSRFAWMTRKSEETITPWNFEEVVDKGASAQSFIERMTNFDKNLP
NEKVLPKHSLLYEYFTVYNELTKVKYVTEGMRKPAFLSGEQKKAIVDLLFKTNRKVTVKQLKEDYFKKIEC
FDSVEISGVEDRFNASLGTYHDLLKIIKDKDFLDNEENEDILEDIVLTLTLFEDREMIEERLKTYAHLFDDKV
MKQLKRRRYTGWGRLSRKLINGIRDKQSGKTILDFLKSDGFANRNFMQLIHDDSLTFKEDIQKAQVSGQG
DSLHEHIANLAGSPAIKKGILQTVKVVDELVKVMGRHKPENIVIEMARENQTTQKGQKNSRERMKRIEEGI
KELGSQILKEHPVENTQLQNEKLYLYYLQNGRDMYVDQELDINRLSDYDVDAIVPQSFLKDDSIDNKVLTR
SDKNRGKSDNVPSEEVVKKMKNYWRQLLNAKLITQRKFDNLTKAERGGLSELDKAGFIKRQLVETRQITK
HVAQILDSRMNTKYDENDKLIREVKVITLKSKLVSDFRKDFQFYKVREINNYHHAHDAYLNAVVGTALIKKY
PKLESEFVYGDYKVYDVRKMIAKSEQEIGKATAKYFFYSNIMNFFKTEITLANGEIRKRPLIETNGETGEIV
WDKGRDFATVRKVLSMPQVNIVKKTEVQTGGFSKESILPKRNSDKLIARKKDWDPKKYGGFDSPTVAYS
VLVVAKVEKGKSKKLKSVKELLGITIMERSSFEKNPIDFLEAKGYKEVKKDLIIKLPKYSLFELENGRKRMLA
SAGELQKGNELALPSKYVNFLYLASHYEKLKGSPEDNEQKQLFVEQHKHYLDEIIEQISEFSKRVILADANL
DKVLSAYNKHRDKPIREQAENIIHLFTLTNLGAPAAFKYFDTTIDRKRYTSTKEVLDATLIHQSITGLYETRID
LSQLGGDKRPAATKKAGQAKKKKSGGGSGGSGSGSDALDDFDLDMLGSDALDDFDLDMLGSDALDDF
DLDMLGSDALDDFDLDMLGSPKKKRKVDYKDDDDK

**MCP-mCherry**: amino acid sequence; **MS2-N55K**, Nuclear Localization Sequence, mCherry

**ASNFTQFVLVDNGGTGDVTVAPSNFANGVAEWISSNSRSQAYKVTCSVRQSSAQKRKYTIKVEVPKVA
TQTVGGVELPVAAWRSYLNMELTIPIFATNSDCELIVKAMQGLLKDGNPIPSAIAANSGIY**SAGGGGSGG
GGSGGGGSGPKKKRKVAAAGSMVSKGEEDNMAIIKEFMRFKVHMEGSVNGHEFEIEGEGEGRPYEGT
QTAKLKVTKGGPLPFAWDILSPQFMYGSKAYVKHPADIPDYLKLSFPEGFKWERVMNFEDGGVVTVTQD
SSLQDGEFIYKVKLRGTNFPSDGPVMQKKTMGWEASSERMYPEDGALKGEIKQRLKLKDGGHYDAEVK
TTYKAKKPVQLPGAYNVNIKLDITSHNEDYTIVEQYERAEGRHSTGGMDELYK

**MCP-MSN**: amino acid sequence; **MS2-N55K**, Nuclear Localization Sequence, MRTF-A, STAT1, eNRF2

**ASNFTQFVLVDNGGTGDVTVAPSNFANGVAEWISSNSRSQAYKVTCSVRQSSAQKRKYTIKVEVPKVA
TQTVGGVELPVAAWRSYLNMELTIPIFATNSDCELIVKAMQGLLKDGNPIPSAIAANSGIY**SAGGGGSGG
GGSGGGGSGPKKKRKVAAAGSSSSQQMDDLFDILIQSGEISADFKEPPSLPGKEKPSPKTVCGSPLAAQ
PSPSAELPQAAPPPPGSPSLPGRLEDFLESSTGLPLLTSGHDGPEPLSLIDDLHSQMLSSTAILDHPPSPM
DTSELHFVPEPSSTMGLDLADGHLDSMDWLELSSGGPVLSLAPLSTTAPSLFSTDFLDGHDLQLHWDSS
GSEVHPSRLQTTDNLLPMSPEEFDEVSRIVGSVEFDSSGSDALYFDDCMQLLAQTFPFVDDNESGGGSG
GSGSSQDIEQVWEELLSIPELQCLNIENDKLVE

**MCP-NMS**: amino acid sequence; **MS2-N55K**, Nuclear Localization Sequence, eNRF2, MRTF-A, STAT1

**ASNFTQFVLVDNGGTGDVTVAPSNFANGVAEWISSNSRSQAYKVTCSVRQSSAQKRKYTIKVEVPKVA
TQTVGGVELPVAAWRSYLNMELTIPIFATNSDCELIVKAMQGLLKDGNPIPSAIAANSGIY**SAGGGGSGG
GGSGGGGSGPKKKRKVAAAGSSDALYFDDCMQLLAQTFPFVDDNESGGGSGGSGSSQDIEQVWEELL
SIPELQCLNIENDKLVESGSSSQQMDDLFDILIQSGEISADFKEPPSLPGKEKPSPKTVCGSPLAAQPSPSA
ELPQAAPPPPGSPSLPGRLEDFLESSTGLPLLTSGHDGPEPLSLIDDLHSQMLSSTAILDHPPSPMDTSEL
HFVPEPSSTMGLDLADGHLDSMDWLELSSGGPVLSLAPLSTTAPSLFSTDFLDGHDLQLHWDSSGSEVH
PSRLQTTDNLLPMSPEEFDEVSRIVGSVEFDS

**MCP-p65-HSF1**: amino acid sequence; **MS2-N55K**, Nuclear Localization Sequence, P65, HSF1

**ASNFTQFVLVDNGGTGDVTVAPSNFANGVAEWISSNSRSQAYKVTCSVRQSSAQKRKYTIKVEVPKVA
TQTVGGVELPVAAWRSYLNMELTIPIFATNSDCELIVKAMQGLLKDGNPIPSAIAANSGIY**SAGGGGSGG
GGSGGGGSGPKKKRKVAAAGSPSGQISNQALALAPSSAPVLAQTMVPSSAMVPLAQPPAPAPVLTPGP
PQSLSAPVPKSTQAGEGTLSEALLHLQFDADEDLGALLGNSTDPGVFTDLASVDNSEFQQLLNQGVSMS
HSTAEPMLMEYPEAITRLVTGSQRPPDPAPTPLGTSGLPNGLSGDEDFSSIADMDFSALLSQISSSGQGG
GGSGFSVDTSALLDLFSPSVTVPDMSLPDLDSSLASIQELLSPQEPPRPPEAENSSPDSGKQLVHYTAQP
LFLLDPGSVDTGSNDLPVLFELGEGSYFSEGDGFAEDPTISLLTGSEPPKAKDPTVS

**MCP-VPR**: amino acid sequence; **MS2-N55K**, Nuclear Localization Sequence, VPR,

**ASNFTQFVLVDNGGTGDVTVAPSNFANGVAEWISSNSRSQAYKVTCSVRQSSAQKRKYTIKVEVPKVA
TQTVGGVELPVAAWRSYLNMELTIPIFATNSDCELIVKAMQGLLKDGNPIPSAIAANSGIY**SAGGGGSGG
GGSGGGGSGPKKKRKVAAAGSDALDDFDLDMLGSDALDDFDLDMLGSDALDDFDLDMLGSDALDDFDL
DMLINSRSSGSPKKKRKVGSQYLPDTDDRHRIEEKRKRTYETFKSIMKKSPFSGPTDPRPPPRRIAVPSR
SSASVPKPAPQPYPFTSSLSTINYDEFPTMVFPSGQISQASALAPAPPQVLPQAPAPAPAPAMVSALAQA
PAPVPVLAPGPPQAVAPPAPKPTQAGEGTLSEALLQLQFDDEDLGALLGNSTDPAVFTDLASVDNSEFQ
QLLNQGIPVAPHTTEPMLMEYPEAITRLVTGAQRPPDPAPAPLGAPGLPNGLLSGDEDFSSIADMDFSALL
GSGSGSRDSREGMFLPKPEAGSAISDVFEGREVCQPKRIRPFHPPGSPWANRPLPASLAPTPTGPVHEP
VGSLTPAPVPQPLDPAPAVTPEASHLLEDPDEETSQAVKALREMADTVIPQKEEAAICGQMDLSHPPPRG
HLDELTTTLESMTEDLNLDSPLTPELNEILDTFLNDECLLHAMHISTGLSIFDTSLF

**SadCas9**: amino acid sequence; *Staphylococcus aureus* Cas9 (D10A, N580A), Nucleoplasmin Nuclear Localization Sequence, Glysine-SerineLinker Sequence, SV40 Nuclear Localization Sequence, 1 X “Flag” Epitope

MKRNYILGLAIGITSVGYGIIDYETRDVIDAGVRLFKEANVENNEGRRSKRGARRLKRRRRHRIQRVKKLLF
DYNLLTDHSELSGINPYEARVKGLSQKLSEEEFSAALLHLAKRRGVHNVNEVEEDTGNELSTKEQISRNS
KALEEKYVAELQLERLKKDGEVRGSINRFKTSDYVKEAKQLLKVQKAYHQLDQSFIDTYIDLLETRRTYYE
GPGEGSPFGWKDIKEWYEMLMGHCTYFPEELRSVKYAYNADLYNALNDLNNLVITRDENEKLEYYEKFQI
IENVFKQKKKPTLKQIAKEILVNEEDIKGYRVTSTGKPEFTNLKVYHDIKDITARKEIIENAELLDQIAKILTIYQ
SSEDIQEELTNLNSELTQEEIEQISNLKGYTGTHNLSLKAINLILDELWHTNDNQIAIFNRLKLVPKKVDLSQ
QKEIPTTLVDDFILSPVVKRSFIQSIKVINAIIKKYGLPNDIIIELAREKNSKDAQKMINEMQKRNRQTNERIEEI
IRTTGKENAKYLIEKIKLHDMQEGKCLYSLEAIPLEDLLNNPFNYEVDHIIPRSVSFDNSFNNKVLVKQEEAS
KKGNRTPFQYLSSSDSKISYETFKKHILNLAKGKGRISKTKKEYLLEERDINRFSVQKDFINRNLVDTRYAT
RGLMNLLRSYFRVNNLDVKVKSINGGFTSFLRRKWKFKKERNKGYKHHAEDALIIANADFIFKEWKKLDKA
KKVMENQMFEEKQAESMPEIETEQEYKEIFITPHQIKHIKDFKDYKYSHRVDKKPNRELINDTLYSTRKDD
KGNTLIVNNLNGLYDKDNDKLKKLINKSPEKLLMYHHDPQTYQKLKLIMEQYGDEKNPLYKYYEETGNYLT
KYSKKDNGPVIKKIKYYGNKLNAHLDITDDYPNSRNKVVKLSLKPYRFDVYLDNGVYKFVTVKNLDVIKKE
NYYEVNSKCYEEAKKLKKISNQAEFIASFYNNDLIKINGELYRVIGVNNDLLNRIEVNMIDITYREYLENMND
KRPPRIIKTIASKTQSIKKYSTDILGNLYEVKSKKHPQIIKKGKRPAATKKAGQAKKKKSGGGSGGSGSGSP
KKKRKVDYKDDDDK

**SadCas9-VP64**: amino acid sequence; *Staphylococcus aureus* Cas9 (D10A, N580A), Nucleoplasmin Nuclear Localization Sequence, Glysine-SerineLinker Sequence, VP64, SV40 Nuclear Localization Sequence, 1 X “Flag” Epitope

MKRNYILGLAIGITSVGYGIIDYETRDVIDAGVRLFKEANVENNEGRRSKRGARRLKRRRRHRIQRVKKLLF
DYNLLTDHSELSGINPYEARVKGLSQKLSEEEFSAALLHLAKRRGVHNVNEVEEDTGNELSTKEQISRNS
KALEEKYVAELQLERLKKDGEVRGSINRFKTSDYVKEAKQLLKVQKAYHQLDQSFIDTYIDLLETRRTYYE
GPGEGSPFGWKDIKEWYEMLMGHCTYFPEELRSVKYAYNADLYNALNDLNNLVITRDENEKLEYYEKFQI
IENVFKQKKKPTLKQIAKEILVNEEDIKGYRVTSTGKPEFTNLKVYHDIKDITARKEIIENAELLDQIAKILTIYQ
SSEDIQEELTNLNSELTQEEIEQISNLKGYTGTHNLSLKAINLILDELWHTNDNQIAIFNRLKLVPKKVDLSQ
QKEIPTTLVDDFILSPVVKRSFIQSIKVINAIIKKYGLPNDIIIELAREKNSKDAQKMINEMQKRNRQTNERIEEI
IRTTGKENAKYLIEKIKLHDMQEGKCLYSLEAIPLEDLLNNPFNYEVDHIIPRSVSFDNSFNNKVLVKQEEAS
KKGNRTPFQYLSSSDSKISYETFKKHILNLAKGKGRISKTKKEYLLEERDINRFSVQKDFINRNLVDTRYAT
RGLMNLLRSYFRVNNLDVKVKSINGGFTSFLRRKWKFKKERNKGYKHHAEDALIIANADFIFKEWKKLDKA
KKVMENQMFEEKQAESMPEIETEQEYKEIFITPHQIKHIKDFKDYKYSHRVDKKPNRELINDTLYSTRKDD
KGNTLIVNNLNGLYDKDNDKLKKLINKSPEKLLMYHHDPQTYQKLKLIMEQYGDEKNPLYKYYEETGNYLT
KYSKKDNGPVIKKIKYYGNKLNAHLDITDDYPNSRNKVVKLSLKPYRFDVYLDNGVYKFVTVKNLDVIKKE
NYYEVNSKCYEEAKKLKKISNQAEFIASFYNNDLIKINGELYRVIGVNNDLLNRIEVNMIDITYREYLENMND
KRPPRIIKTIASKTQSIKKYSTDILGNLYEVKSKKHPQIIKKGKRPAATKKAGQAKKKKSGGGSGGSGSGSD
ALDDFDLDMLGSDALDDFDLDMLGSDALDDFDLDMLGSDALDDFDLDMLGSPKKKRKVDYKDDDDK

**SadCas9-VPR**: amino acid sequence; *Staphylococcus aureus* Cas9 (D10A, N580A), Nucleoplasmin Nuclear Localization Sequence, Glysine-SerineLinker Sequence, VPR, SV40 Nuclear Localization Sequence, 1 X “Flag” Epitope

MKRNYILGLAIGITSVGYGIIDYETRDVIDAGVRLFKEANVENNEGRRSKRGARRLKRRRRHRIQRVKKLLF
DYNLLTDHSELSGINPYEARVKGLSQKLSEEEFSAALLHLAKRRGVHNVNEVEEDTGNELSTKEQISRNS
KALEEKYVAELQLERLKKDGEVRGSINRFKTSDYVKEAKQLLKVQKAYHQLDQSFIDTYIDLLETRRTYYE
GPGEGSPFGWKDIKEWYEMLMGHCTYFPEELRSVKYAYNADLYNALNDLNNLVITRDENEKLEYYEKFQI
IENVFKQKKKPTLKQIAKEILVNEEDIKGYRVTSTGKPEFTNLKVYHDIKDITARKEIIENAELLDQIAKILTIYQ
SSEDIQEELTNLNSELTQEEIEQISNLKGYTGTHNLSLKAINLILDELWHTNDNQIAIFNRLKLVPKKVDLSQ
QKEIPTTLVDDFILSPVVKRSFIQSIKVINAIIKKYGLPNDIIIELAREKNSKDAQKMINEMQKRNRQTNERIEEI

IRTTGKENAKYLIEKIKLHDMQEGKCLYSLEAIPLEDLLNNPFNYEVDHIIPRSVSFDNSFNNKVLVKQEEAS
KKGNRTPFQYLSSSDSKISYETFKKHILNLAKGKGRISKTKKEYLLEERDINRFSVQKDFINRNLVDTRYAT
RGLMNLLRSYFRVNNLDVKVKSINGGFTSFLRRKWKFKKERNKGYKHHAEDALIIANADFIFKEWKKLDKA
KKVMENQMFEEKQAESMPEIETEQEYKEIFITPHQIKHIKDFKDYKYSHRVDKKPNRELINDTLYSTRKDD
KGNTLIVNNLNGLYDKDNDKLKKLINKSPEKLLMYHHDPQTYQKLKLIMEQYGDEKNPLYKYYEETGNYLT
KYSKKDNGPVIKKIKYYGNKLNAHLDITDDYPNSRNKVVKLSLKPYRFDVYLDNGVYKFVTVKNLDVIKKE
NYYEVNSKCYEEAKKLKKISNQAEFIASFYNNDLIKINGELYRVIGVNNDLLNRIEVNMIDITYREYLENMND
KRPPRIIKTIASKTQSIKKYSTDILGNLYEVKSKKHPQIIKKGKRPAATKKAGQAKKKKSGGGSGGSGSGSD
ALDDFDLDMLGSDALDDFDLDMLGSDALDDFDLDMLGSDALDDFDLDMLINSRSSGSPKKKRKVGSQYL
PDTDDRHRIEEKRKRTYETFKSIMKKSPFSGPTDPRPPPRRIAVPSRSSASVPKPAPQPYPFTSSLSTINY
DEFPTMVFPSGQISQASALAPAPPQVLPQAPAPAPAPAMVSALAQAPAPVPVLAPGPPQAVAPPAPKPT
QAGEGTLSEALLQLQFDDEDLGALLGNSTDPAVFTDLASVDNSEFQQLLNQGIPVAPHTTEPMLMEYPEA
ITRLVTGAQRPPDPAPAPLGAPGLPNGLLSGDEDFSSIADMDFSALLGSGSGSRDSREGMFLPKPEAGS
AISDVFEGREVCQPKRIRPFHPPGSPWANRPLPASLAPTPTGPVHEPVGSLTPAPVPQPLDPAPAVTPEA
SHLLEDPDEETSQAVKALREMADTVIPQKEEAAICGQMDLSHPPPRGHLDELTTTLESMTEDLNLDSPLT
PELNEILDTFLNDECLLHAMHISTGLSIFDTSLFGSPKKKRKVDYKDDDDK

**CjdCas9**: amino acid sequence; *Campylobacter jejuni* Cas9 (D8A, H559A), Nucleoplasmin Nuclear Localization Sequence, Glysine-SerineLinker Sequence, SV40 Nuclear Localization Sequence, 1 X “Flag” Epitope

MARILAFAIGISSIGWAFSENDELKDCGVRIFTKVENPKTGESLALPRRLARSARKRLARRKARLNHLKHLI
ANEFKLNYEDYQSFDESLAKAYKGSLISPYELRFRALNELLSKQDFARVILHIAKRRGYDDIKNSDDKEKG
AILKAIKQNEEKLANYQSVGEYLYKEYFQKFKENSKEFTNVRNKKESYERCIAQSFLKDELKLIFKKQREFG
FSFSKKFEEEVLSVAFYKRALKDFSHLVGNCSFFTDEKRAPKNSPLAFMFVALTRIINLLNNLKNTEGILYT
KDDLNALLNEVLKNGTLTYKQTKKLLGLSDDYEFKGEKGTYFIEFKKYKEFIKALGEHNLSQDDLNEIAKDI
TLIKDEIKLKKALAKYDLNQNQIDSLSKLEFKDHLNISFKALKLVTPLMLEGKKYDEACNELNLKVAINEDKK
DFLPAFNETYYKDEVTNPVVLRAIKEYRKVLNALLKKYGKVHKINIELAREVGKNHSQRAKIEKEQNENYK
AKKDAELECEKLGLKINSKNILKLRLFKEQKEFCAYSGEKIKISDLQDEKMLEIDAIYPYSRSFDDSYMNKVL
VFTKQNQEKLNQTPFEAFGNDSAKWQKIEVLAKNLPTKKQKRILDKNYKDKEQKNFKDRNLNDTRYIARL
VLNYTKDYLDFLPLSDDENTKLNDTQKGSKVHVEAKSGMLTSALRHTWGFSAKDRNNHLHHAIDAVIIAY
ANNSIVKAFSDFKKEQESNSAELYAKKISELDYKNKRKFFEPFSGFRQKVLDKIDEIFVSKPERKKPSGALH
EETFRKEEEFYQSYGGKEGVLKALELGKIRKVNGKIVKNGDMFRVDIFKHKKTNKFYAVPIYTMDFALKVL
PNKAVARSKKGEIKDWILMDENYEFCFSLYKDSLILIQTKDMQEPEFVYYNAFTSSTVSLIVSKHDNKFETL
SKNQKILFKNANEKEVIAKSIGIQNLKVFEKYIVSALGEVTKAEFRQREDFKKKRPAATKKAGQAKKKKSG
GGSGGSGSGSPKKKRKVDYKDDDDK

**CjdCas9-VP64**: amino acid sequence; *Campylobacter jejuni* Cas9 (D8A, N559A), Nucleoplasmin Nuclear Localization Sequence, Glysine-SerineLinker Sequence, VP64, SV40 Nuclear Localization Sequence, 1 X “Flag” Epitope

MARILAFAIGISSIGWAFSENDELKDCGVRIFTKVENPKTGESLALPRRLARSARKRLARRKARLNHLKHLI
ANEFKLNYEDYQSFDESLAKAYKGSLISPYELRFRALNELLSKQDFARVILHIAKRRGYDDIKNSDDKEKG
AILKAIKQNEEKLANYQSVGEYLYKEYFQKFKENSKEFTNVRNKKESYERCIAQSFLKDELKLIFKKQREFG
FSFSKKFEEEVLSVAFYKRALKDFSHLVGNCSFFTDEKRAPKNSPLAFMFVALTRIINLLNNLKNTEGILYT
KDDLNALLNEVLKNGTLTYKQTKKLLGLSDDYEFKGEKGTYFIEFKKYKEFIKALGEHNLSQDDLNEIAKDI
TLIKDEIKLKKALAKYDLNQNQIDSLSKLEFKDHLNISFKALKLVTPLMLEGKKYDEACNELNLKVAINEDKK
DFLPAFNETYYKDEVTNPVVLRAIKEYRKVLNALLKKYGKVHKINIELAREVGKNHSQRAKIEKEQNENYK
AKKDAELECEKLGLKINSKNILKLRLFKEQKEFCAYSGEKIKISDLQDEKMLEIDAIYPYSRSFDDSYMNKVL
VFTKQNQEKLNQTPFEAFGNDSAKWQKIEVLAKNLPTKKQKRILDKNYKDKEQKNFKDRNLNDTRYIARL
VLNYTKDYLDFLPLSDDENTKLNDTQKGSKVHVEAKSGMLTSALRHTWGFSAKDRNNHLHHAIDAVIIAY
ANNSIVKAFSDFKKEQESNSAELYAKKISELDYKNKRKFFEPFSGFRQKVLDKIDEIFVSKPERKKPSGALH
EETFRKEEEFYQSYGGKEGVLKALELGKIRKVNGKIVKNGDMFRVDIFKHKKTNKFYAVPIYTMDFALKVL
PNKAVARSKKGEIKDWILMDENYEFCFSLYKDSLILIQTKDMQEPEFVYYNAFTSSTVSLIVSKHDNKFETL

SKNQKILFKNANEKEVIAKSIGIQNLKVFEKYIVSALGEVTKAEFRQREDFKKKRPAATKKAGQAKKKKSG
GGSGGSGSGSDALDDFDLDMLGSDALDDFDLDMLGSDALDDFDLDMLGSDALDDFDLDMLGSPKKKRK
VDYKDDDDK

**CjdCas9-VPR**: amino acid sequence; *Campylobacter jejuni* Cas9 (D8A, N559A), Nucleoplasmin Nuclear Localization Sequence, Glysine-SerineLinker Sequence, VPR, SV40 Nuclear Localization Sequence, 1 X "Flag" Epitope

MARILAFAIGISSIGWAFSENDELKDCGVRIFTKVENPKTGESLALPRRLARSARKRLARRKARLNHLKHLI
ANEFKLNYEDYQSFDESLAKAYKGSLISPYELRFRALNELLSKQDFARVILHIAKRRGYDDIKNSDDKEKG
AILKAIKQNEEKLANYQSVGEYLYKEYFQKFKENSKEFTNVRNKKESYERCIAQSFLKDELKLIFKKQREFG
FSFSKKFEEEVLSVAFYKRALKDFSHLVGNCSFFTDEKRAPKNSPLAFMFVALTRIINLLNNLKNTEGILYT
KDDLNALLNEVLKNGTLTYKQTKKLLGLSDDYEFKGEKGTYFIEFKKYKEFIKALGEHNLSQDDLNEIAKDI
TLIKDEIKLKKALAKYDLNQNQIDSLSKLEFKDHLNISFKALKLVTPLMLEGKKYDEACNELNLKVAINEDKK
DFLPAFNETYYKDEVTNPVVLRAIKEYRKVLNALLKKYGKVHKINIELAREVGKNHSQRAKIEKEQNENYK
AKKDAELECEKLGLKINSKNILKLRLFKEQKEFCAYSGEKIKISDLQDEKMLEIDAIYPYSRSFDDSYMNKVL
VFTKQNQEKLNQTPFEAFGNDSAKWQKIEVLAKNLPTKKQKRILDKNYKDKEQKNFKDRNLNDTRYIARL
VLNYTKDYLDFLPLSDDENTKLNDTQKGSKVHVEAKSGMLTSALRHTWGFSAKDRNNHLHHAIDAVIIAY
ANNSIVKAFSDFKKEQESNSAELYAKKISELDYKNKRKFFEPFSGFRQKVLDKIDEIFVSKPERKKPSGALH
EETFRKEEEFYQSYGGKEGVLKALELGKIRKVNGKIVKNGDMFRVDIFKHKKTNKFYAVPIYTMDFALKVL
PNKAVARSKKGEIKDWILMDENYEFCFSLYKDSLILIQTKDMQEPEFVYYNAFTSSTVSLIVSKHDNKFETL
SKNQKILFKNANEKEVIAKSIGIQNLKVFEKYIVSALGEVTKAEFRQREDFKKKRPAATKKAGQAKKKKSG
GGSGGSGSGSDALDDFDLDMLGSDALDDFDLDMLGSDALDDFDLDMLGSDALDDFDLDMLINSRSSGS
PKKKRKVGSQYLPDTDDRHRIEEKRKRTYETFKSIMKKSPFSGPTDPRPPPRRIAVPSRSSASVPKPAPQ
PYPFTSSLSTINYDEFPTMVFPSGQISQASALAPAPPQVLPQAPAPAPAPAMVSALAQAPAPVPVLAPGP
PQAVAPPAPKPTQAGEGTLSEALLQLQFDDEDLGALLGNSTDPAVFTDLASVDNSEFQQLLNQGIPVAPH
TTEPMLMEYPEAITRLVTGAQRPPDPAPAPLGAPGLPNGLLSGDEDFSSIADMDFSALLGSGSGSRDSRE
GMFLPKPEAGSAISDVFEGREVCQPKRIRPFHPPGSPWANRPLPASLAPTPTGPVHEPVGSLTPAPVPQ
PLDPAPAVTPEASHLLEDPDEETSQAVKALREMADTVIPQKEEAAICGQMDLSHPPPRGHLDELTTTLES
MTEDLNLDSPLTPELNEILDTFLNDECLLHAMHISTGLSIFDTSLFGSPKKKRKVDYKDDDDK

**MCP-3x 9aa TAD**: amino acid sequence; **MS2-N55K**, Nuclear Localization Sequence, 3x 9aa TAD (MRTF-B.3, MYOCD.1, MYOCD.3)

**ASNFTQFVLVDNGGTGDVTVAPSNFANGVAEWISSNSRSQAYKVTCSVRQSSAQKRKYTIKVEVPKVA**
**TQTVGGVELPVAAWRSYLNMELTIPIFATNSDCELIVKAMQGLLKDGNPIPSAIAANSGIY**SAGGGGSGG
GGSGGGGSGPKKKRKVAAAGSSMFSADFLDSGDELLDVLIESGSIFNIDFLD

**MCP-eN3x9**: amino acid sequence; **MS2-N55K**, Nuclear Localization Sequence, eNRF2, 3x 9aa TAD (MRTF-B.3, MYOCD.1, MYOCD.3)

**ASNFTQFVLVDNGGTGDVTVAPSNFANGVAEWISSNSRSQAYKVTCSVRQSSAQKRKYTIKVEVPKVA**
**TQTVGGVELPVAAWRSYLNMELTIPIFATNSDCELIVKAMQGLLKDGNPIPSAIAANSGIY**SAGGGGSGG
GGSGGGGSGPKKKRKVAAAGSSDALYFDDCMQLLAQTFPFVDDNESGGGSGGSGSSQDIEQVWEELL
SIPELQCLNIENDKLVESGSMFSADFLDSGDELLDVLIESGSIFNIDFLD

**HNH domain deleted (792-897, without linker) SpdCas9**: amino acid sequence; Streptococcus pyogenes Cas9 (D10A,), Nucleoplasmin Nuclear Localization Sequence, Glysine-Serine Linker Sequence, SV40 Nuclear Localization Sequence, 1 X "Flag" Epitope

MDKKYSIGLAIGTNSVGWAVITDEYKVPSKKFKVLGNTDRHSIKKNLIGALLFDSGETAEATRLKRTARRR
YTRRKNRICYLQEIFSNEMAKVDDSFFHRLEESFLVEEDKKHERHPIFGNIVDEVAYHEKYPTIYHLRKKLV
DSTDKADLRLIYLALAHMIKFRGHFLIEGDLNPDNSDVDKLFIQLVQTYNQLFEENPINASGVDAKAILSARL

SKSRRLENLIAQLPGEKKNGLFGNLIALSLGLTPNFKSNFDLAEDAKLQLSKDTYDDDLDNLLAQIGDQYA
DLFLAAKNLSDAILLSDILRVNTEITKAPLSASMIKRYDEHHQDLTLLKALVRQQLPEKYKEIFFDQSKNGYA
GYIDGGASQEEFYKFIKPILEKMDGTEELLVKLNREDLLRKQRTFDNGSIPHQIHLGELHAILRRQEDFYPF
LKDNREKIEKILTFRIPYYVGPLARGNSRFAWMTRKSEETITPWNFEEVVDKGASAQSFIERMTNFDKNLP
NEKVLPKHSLLYEYFTVYNELTKVKYVTEGMRKPAFLSGEQKKAIVDLLFKTNRKVTVKQLKEDYFKKIEC
FDSVEISGVEDRFNASLGTYHDLLKIIKDKDFLDNEENEDILEDIVLTLTLFEDREMIEERLKTYAHLFDDKV
MKQLKRRRYTGWGRLSRKLINGIRDKQSGKTILDFLKSDGFANRNFMQLIHDDSLTFKEDIQKAQVSGQG
DSLHEHIANLAGSPAIKKGILQTVKVVDELVKVMGRHKPENIVIEMARENQTTQKGQKNSRERMKRIEEGI
KELDNLTKAERGGLSELDKAGFIKRQLVETRQITKHVAQILDSRMNTKYDENDKLIREVKVITLKSKLVSDF
RKDFQFYKVREINNYHHAHDAYLNAVVGTALIKKYPKLESEFVYGDYKVYDVRKMIAKSEQEIGKATAKYF
FYSNIMNFFKTEITLANGEIRKRPLIETNGETGEIVWDKGRDFATVRKVLSMPQVNIVKKTEVQTGGFSKES
ILPKRNSDKLIARKKDWDPKKYGGFDSPTVAYSVLVVAKVEKGKSKKLKSVKELLGITIMERSSFEKNPIDF
LEAKGYKEVKKDLIIKLPKYSLFELENGRKRMLASAGELQKGNELALPSKYVNFLYLASHYEKLKGSPEDN
EQKQLFVEQHKHYLDEIIEQISEFSKRVILADANLDKVLSAYNKHRDKPIREQAENIIHLFTLTNLGAPAAFK
YFDTTIDRKRYTSTKEVLDATLIHQSITGLYETRIDLSQLGGDKRPAATKKAGQAKKKKSGGGSGGSGSG
SPKKKRKVDYKDDDDK

**Mini-DREAM Compact**: amino acid sequence; Streptococcus pyogenes Cas9 (D10A,), Nucleoplasmin Nuclear Localization Sequence, Glysine-Serine Linker Sequence, SV40 Nuclear Localization Sequence, 1 X “Flag” Epitope; **P2A**, **MS2-N55K**, Nuclear Localization Sequence, eNRF2, 3x 9aa TAD (MRTF-B.3, MYOCD.1, MYOCD.3)

MDKKYSIGLAIGTNSVGWAVITDEYKVPSKKFKVLGNTDRHSIKKNLIGALLFDSGETAEATRLKRTARRR
YTRRKNRICYLQEIFSNEMAKVDDSFFHRLEESFLVEEDKKHERHPIFGNIVDEVAYHEKYPTIYHLRKKLV
DSTDKADLRLIYLALAHMIKFRGHFLIEGDLNPDNSDVDKLFIQLVQTYNQLFEENPINASGVDAKAILSARL
SKSRRLENLIAQLPGEKKNGLFGNLIALSLGLTPNFKSNFDLAEDAKLQLSKDTYDDDLDNLLAQIGDQYA
DLFLAAKNLSDAILLSDILRVNTEITKAPLSASMIKRYDEHHQDLTLLKALVRQQLPEKYKEIFFDQSKNGYA
GYIDGGASQEEFYKFIKPILEKMDGTEELLVKLNREDLLRKQRTFDNGSIPHQIHLGELHAILRRQEDFYPF
LKDNREKIEKILTFRIPYYVGPLARGNSRFAWMTRKSEETITPWNFEEVVDKGASAQSFIERMTNFDKNLP
NEKVLPKHSLLYEYFTVYNELTKVKYVTEGMRKPAFLSGEQKKAIVDLLFKTNRKVTVKQLKEDYFKKIEC
FDSVEISGVEDRFNASLGTYHDLLKIIKDKDFLDNEENEDILEDIVLTLTLFEDREMIEERLKTYAHLFDDKV
MKQLKRRRYTGWGRLSRKLINGIRDKQSGKTILDFLKSDGFANRNFMQLIHDDSLTFKEDIQKAQVSGQG
DSLHEHIANLAGSPAIKKGILQTVKVVDELVKVMGRHKPENIVIEMARENQTTQKGQKNSRERMKRIEEGI
KELDNLTKAERGGLSELDKAGFIKRQLVETRQITKHVAQILDSRMNTKYDENDKLIREVKVITLKSKLVSDF
RKDFQFYKVREINNYHHAHDAYLNAVVGTALIKKYPKLESEFVYGDYKVYDVRKMIAKSEQEIGKATAKYF
FYSNIMNFFKTEITLANGEIRKRPLIETNGETGEIVWDKGRDFATVRKVLSMPQVNIVKKTEVQTGGFSKES
ILPKRNSDKLIARKKDWDPKKYGGFDSPTVAYSVLVVAKVEKGKSKKLKSVKELLGITIMERSSFEKNPIDF
LEAKGYKEVKKDLIIKLPKYSLFELENGRKRMLASAGELQKGNELALPSKYVNFLYLASHYEKLKGSPEDN
EQKQLFVEQHKHYLDEIIEQISEFSKRVILADANLDKVLSAYNKHRDKPIREQAENIIHLFTLTNLGAPAAFK
YFDTTIDRKRYTSTKEVLDATLIHQSITGLYETRIDLSQLGGDKRPAATKKAGQAKKKKSGGGSGGSGSG
SPKKKRKVDYKDDDDK**EGRGSLLTCGDVEENPGPASNFTQFVLVDNGGTGDVTVAPSNFANGVAEWIS
SNSRSQAYKVTCSVRQSSAQKRKYTIKVEVPKVATQTVGGVELPVAAWRSYLNMELTIPIFATNSDCELI
VKAMQGLLKDGNPIPSAIAANSGIY**SAGGGGSGGGGSGGGGSGPKKKRKVAAAGSSDALYFDDCMQL
LAQTFPFVDDNESGGGSGGSGSSQDIEQVWEELLSIPELQCLNIENDKLVESGSMFSADFLDSGDELLDV
LIESGSIFNIDFLD

***Eco*-Cascade Cas6-MSN**: amino acid sequence; Cas6, Nucleoplasmin Nuclear Localization Sequence, Glysine-Serine Linker Sequence, MRTF-A, STAT1, eNRF2, 1 X “Flag” Epitope

MYLSKVIIARAWSRDLYQLHQGLWHLFPNRPDAARDFLFHVEKRNTPEGCHVLLQSAQMPVSTAVATVIK
TKQVEFQLQVGVPLYFRLRANPIKTILDNQKRLDSKGNIKRCRVPLIKEAEQIAWLQRKLGNAARVEDVHPI
SERPQYFSGDGKSGKIQTVCFEGVLTINDAPALIDLVQQGIGPAKSMGCGLLSLAPLRGKRPAATKKAGQ
AKKKKGSSSSQQMDDLFDILIQSGEISADFKEPPSLPGKEKPSPKTVCGSPLAAQPSPSAELPQAAPPPP
GSPSLPGRLEDFLESSTGLPLLTSGHDGPEPLSLIDDLHSQMLSSTAILDHPPSPMDTSELHFVPEPSSTM

GLDLADGHLDSMDWLELSSGGPVLSLAPLSTTAPSLFSTDFLDGHDLQLHWDSSGSEVHPSRLQTTDNL
LPMSPEEFDEVSRIVGSVEFDSSGSDALYFDDCMQLLAQTFPFVDDNESGGGSGGSGSSQDIEQVWEE
LLSIPELQCLNIENDKLVEGSDYKDDDDK

***Eco*-Cascade Cas6-NMS**: amino acid sequence; Cas6, Nucleoplasmin Nuclear Localization Sequence, Glysine-Serine Linker Sequence, eNRF2, MRTF-A, STAT1, 1 X "Flag" Epitope

MYLSKVIIARAWSRDLYQLHQGLWHLFPNRPDAARDFLFHVEKRNTPEGCHVLLQSAQMPVSTAVATVIK
TKQVEFQLQVGVPLYFRLRANPIKTILDNQKRLDSKGNIKRCRVPLIKEAEQIAWLQRKLGNAARVEDVHPI
SERPQYFSGDGKSGKIQTVCFEGVLTINDAPALIDLVQQGIGPAKSMGCGLLSLAPLRGKRPAATKKAGQ
AKKKKGSSDALYFDDCMQLLAQTFPFVDDNESGGGSGGSGSSQDIEQVWEELLSIPELQCLNIENDKLV
ESGSSSQQMDDLFDILIQSGEISADFKEPPSLPGKEKPSPKTVCGSPLAAQPSPSAELPQAAPPPPGSPS
LPGRLEDFLESSTGLPLLTSGHDGPEPLSLIDDLHSQMLSSTAILDHPPSPMDTSELHFVPEPSSTMGLDL
ADGHLDSMDWLELSSGGPVLSLAPLSTTAPSLFSTDFLDGHDLQLHWDSSGSEVHPSRLQTTDNLLPMS
PEEFDEVSRIVGSVEFDSGSDYKDDDDK

**AsdCas12a-MSN**: amino acid sequence; AsdCas12a(G993A), Nucleoplasmin Nuclear Localization Sequence, Glysine-Serine Linker Sequence, SV40 Nuclear Localization Sequence, MRTF-A, STAT1, eNRF2, **HA-tag**

MTQFEGFTNLYQVSKTLRFELIPQGKTLKHIQEQGFIEEDKARNDHYKELKPIIDRIYKTYADQCLQLVQLD
WENLSAAIDSYRKEKTEETRNALIEEQATYRNAIHDYFIGRTDNLTDAINKRHAEIYKGLFKAELFNGKVLK
QLGTVTTTEHENALLRSFDKFTTYFSGFYENRKNVFSAEDISTAIPHRIVQDNFPKFKENCHIFTRLITAVPS
LREHFENVKKAIGIFVSTSIEEVFSFPFYNQLLTQTQIDLYNQLLGGISREAGTEKIKGLNEVLNLAIQKNDET
AHIIASLPHRFIPLFKQILSDRNTLSFILEEFKSDEEVIQSFCKYKTLLRNENVLETAEALFNELNSIDLTHIFIS
HKKLETISSALCDHWDTLRNALYERRISELTGKITKSAKEKVQRSLKHEDINLQEIISAAGKELSEAFKQKTS
EILSHAHAALDQPLPTTLKKQEEKEILKSQLDSLLGLYHLLDWFAVDESNEVDPEFSARLTGIKLEMEPSLS
FYNKARNYATKKPYSVEKFKLNFQMPTLASGWDVNKEKNNGAILFVKNGLYYLGIMPKQKGRYKALSFEP
TEKTSEGFDKMYYDYFPDAAKMIPKCSTQLKAVTAHFQTHTTPILLSNNFIEPLEITKEIYDLNNPEKEPKKF
QTAYAKKTGDQKGYREALCKWIDFTRDFLSKYTKTTSIDLSSLRPSSQYKDLGEYYAELNPLLYHISFQRIA
EKEIMDAVETGKLYLFQIYNKDFAKGHHGKPNLHTLYWTGLFSPENLAKTSIKLNGQAELFYRPKSRMKR
MAHRLGEKMLNKKLKDQKTPIPDTLYQELYDYVNHRLSHDLSDEARALLPNVITKEVSHEIIKDRRFTSDK
FFFHVPITLNYQAANSPSKFNQRVNAYLKEHPETPIIGIDRGERNLIYITVIDSTGKILEQRSLNTIQQFDYQK
KLDNREKERVAARQAWSVVGTIKDLKQGYLSQVIHEIVDLMIHYQAVVVLANLNFGFKSKRTGIAEKAVYQ
QFEKMLIDKLNCLVLKDYPAEKVGGVLNPYQLTDQFTSFAKMGTQSGFLFYVPAPYTSKIDPLTGFVDPFV
WKTIKNHESRKHFLEGFDFLHYDVKTGDFILHFKMNRNLSFQRGLPGFMPAWDIVFEKNETQFDAKGTPF
IAGKRIVPVIENHRFTGRYRDLYPANELIALLEEKGIVFRDGSNILPKLLENDDSHAIDTMVALIRSVLQMRN
SNAATGEDYINSPVRDLNGVCFDSRFQNPEWPMDADANGAYHIALKGQLLLNHLKESKDLKLQNGISNQ
DWLAYIQELRNKRPAATKKAGQAKKKKSAGGGGSGGGGSGGGGSGPKKKRKVAAAGSSSSQQMDDLF
DILIQSGEISADFKEPPSLPGKEKPSPKTVCGSPLAAQPSPSAELPQAAPPPPGSPSLPGRLEDFLESSTG
LPLLTSGHDGPEPLSLIDDLHSQMLSSTAILDHPPSPMDTSELHFVPEPSSTMGLDLADGHLDSMDWLEL
SSGGPVLSLAPLSTTAPSLFSTDFLDGHDLQLHWDSSGSEVHPSRLQTTDNLLPMSPEEFDEVSRIVGSV
EFDSSGSDALYFDDCMQLLAQTFPFVDDNESGGGSGGSGSSQDIEQVWEELLSIPELQCLNIENDKLVE
ASGSGEGS**YPYDVPDYAYPYDVPDYAYPYDVPDYA**

**AsdCas12a-NMS**: amino acid sequence; AsdCas12a(G993A), Nucleoplasmin Nuclear Localization Sequence, Glysine-Serine Linker Sequence, SV40 Nuclear Localization Sequence, eNRF2, MRTF-A, STAT1, **HA-tag**

MTQFEGFTNLYQVSKTLRFELIPQGKTLKHIQEQGFIEEDKARNDHYKELKPIIDRIYKTYADQCLQLVQLD
WENLSAAIDSYRKEKTEETRNALIEEQATYRNAIHDYFIGRTDNLTDAINKRHAEIYKGLFKAELFNGKVLK
QLGTVTTTEHENALLRSFDKFTTYFSGFYENRKNVFSAEDISTAIPHRIVQDNFPKFKENCHIFTRLITAVPS
LREHFENVKKAIGIFVSTSIEEVFSFPFYNQLLTQTQIDLYNQLLGGISREAGTEKIKGLNEVLNLAIQKNDET
AHIIASLPHRFIPLFKQILSDRNTLSFILEEFKSDEEVIQSFCKYKTLLRNENVLETAEALFNELNSIDLTHIFIS

HKKLETISSALCDHWDTLRNALYERRISELTGKITKSAKEKVQRSLKHEDINLQEIISAAGKELSEAFKQKTS
EILSHAHAALDQPLPTTLKKQEEKEILKSQLDSLLGLYHLLDWFAVDESNEVDPEFSARLTGIKLEMEPSLS
FYNKARNYATKKPYSVEKFKLNFQMPTLASGWDVNKEKNNGAILFVKNGLYYLGIMPKQKGRYKALSFEP
TEKTSEGFDKMYYDYFPDAAKMIPKCSTQLKAVTAHFQTHTTPILLSNNFIEPLEITKEIYDLNNPEKEPKKF
QTAYAKKTGDQKGYREALCKWIDFTRDFLSKYTKTTSIDLSSLRPSSQYKDLGEYYAELNPLLYHISFQRIA
EKEIMDAVETGKLYLFQIYNKDFAKGHHGKPNLHTLYWTGLFSPENLAKTSIKLNGQAELFYRPKSRMKR
MAHRLGEKMLNKKLKDQKTPIPDTLYQELYDYVNHRLSHDLSDEARALLPNVITKEVSHEIIKDRRFTSDK
FFFHVPITLNYQAANSPSKFNQRVNAYLKEHPETPIIGIDRGERNLIYITVIDSTGKILEQRSLNTIQQFDYQK
KLDNREKERVAARQAWSVVGTIKDLKQGYLSQVIHEIVDLMIHYQAVVVLANLNFGFKSKRTGIAEKAVYQ
QFEKMLIDKLNCLVLKDYPAEKVGGVLNPYQLTDQFTSFAKMGTQSGFLFYVPAPYTSKIDPLTGFVDPFV
WKTIKNHESRKHFLEGFDFLHYDVKTGDFILHFKMNRNLSFQRGLPGFMPAWDIVFEKNETQFDAKGTPF
IAGKRIVPVIENHRFTGRYRDLYPANELIALLEEKGIVFRDGSNILPKLLENDDSHAIDTMVALIRSVLQMRN
SNAATGEDYINSPVRDLNGVCFDSRFQNPEWPMDADANGAYHIALKGQLLLNHLKESKDLKLQNGISNQ
DWLAYIQELRNKRPAATKKAGQAKKKKSAGGGGSGGGGSGGGGSGPKKKRKVAAAGSSDALYFDDCM
QLLAQTFPFVDDNESGGGSGGSGSSQDIEQVWEELLSIPELQCLNIENDKLVESGSSSQQMDDLFDILIQ
SGEISADFKEPPSLPGKEKPSPKTVCGSPLAAQPSPSAELPQAAPPPPGSPSLPGRLEDFLESSTGLPLLT
SGHDGPEPLSLIDDLHSQMLSSTAILDHPPSPMDTSELHFVPEPSSTMGLDLADGHLDSMDWLELSSGG
PVLSLAPLSTTAPSLFSTDFLDGHDLQLHWDSSGSEVHPSRLQTTDNLLPMSPEEFDEVSRIVGSVEFDS
SGSDALYFDDCMQLLAQTFPFVDDNESGGGSGGSGSSQDIEQVWEELLSIPELQCLNIENDKLVEASGS
GEGS**YPYDVPDYAYPYDVPDYAYPYDVPDYA**

**SpdCas9-VPR**: amino acid sequence; Streptococcus pyogenes Cas9 (D10A, H840A), Nucleoplasmin Nuclear Localization Sequence, Glysine-Serine Linker Sequence, VPR, SV40 Nuclear Localization Sequence, 1 X “Flag” Epitope

MDYKDDDDKPKKKRKVTGSRALSGGGSGGSGSDKKYSIGLAIGTNSVGWAVITDEYKVPSKKFKVLGNT
DRHSIKKNLIGALLFDSGETAEATRLKRTARRRYTRRKNRICYLQEIFSNEMAKVDDSFFHRLEESFLVEE
DKKHERHPIFGNIVDEVAYHEKYPTIYHLRKKLVDSTDKADLRLIYLALAHMIKFRGHFLIEGDLNPDNSDV
DKLFIQLVQTYNQLFEENPINASGVDAKAILSARLSKSRRLENLIAQLPGEKKNGLFGNLIALSLGLTPNFKS
NFDLAEDAKLQLSKDTYDDDLDNLLAQIGDQYADLFLAAKNLSDAILLSDILRVNTEITKAPLSASMIKRYDE
HHQDLTLLKALVRQQLPEKYKEIFFDQSKNGYAGYIDGGASQEEFYKFIKPILEKMDGTEELLVKLNREDLL
RKQRTFDNGSIPHQIHLGELHAILRRQEDFYPFLKDNREKIEKILTFRIPYYVGPLARGNSRFAWMTRKSEE
TITPWNFEEVVDKGASAQSFIERMTNFDKNLPNEKVLPKHSLLYEYFTVYNELTKVKYVTEGMRKPAFLS
GEQKKAIVDLLFKTNRKVTVKQLKEDYFKKIECFDSVEISGVEDRFNASLGTYHDLLKIIKDKDFLDNEENE
DILEDIVLTLTLFEDREMIEERLKTYAHLFDDKVMKQLKRRRYTGWGRLSRKLINGIRDKQSGKTILDFLKS
DGFANRNFMQLIHDDSLTFKEDIQKAQVSGQGDSLHEHIANLAGSPAIKKGILQTVKVVDELVKVMGRHK
PENIVIEMARENQTTQKGQKNSRERMKRIEEGIKELGSQILKEHPVENTQLQNEKLYLYYLQNGRDMYVD
QELDINRLSDYDVDAIVPQSFLKDDSIDNKVLTRSDKNRGKSDNVPSEEVVKKMKNYWRQLLNAKLITQR
KFDNLTKAERGGLSELDKAGFIKRQLVETRQITKHVAQILDSRMNTKYDENDKLIREVKVITLKSKLVSDFR
KDFQFYKVREINNYHHAHDAYLNAVVGTALIKKYPKLESEFVYGDYKVYDVRKMIAKSEQEIGKATAKYFF
YSNIMNFFKTEITLANGEIRKRPLIETNGETGEIVWDKGRDFATVRKVLSMPQVNIVKKTEVQTGGFSKESI
LPKRNSDKLIARKKDWDPKKYGGFDSPTVAYSVLVVAKVEKGKSKKLKSVKELLGITIMERSSFEKNPIDF
LEAKGYKEVKKDLIIKLPKYSLFELENGRKRMLASAGELQKGNELALPSKYVNFLYLASHYEKLKGSPEDN
EQKQLFVEQHKHYLDEIIEQISEFSKRVILADANLDKVLSAYNKHRDKPIREQAENIIHLFTLTNLGAPAAFK
YFDTTIDRKRYTSTKEVLDATLIHQSITGLYETRIDLSQLGGDKRPAATKKAGQAKKKKSGGGSGGSGSG
SDALDDFDLDMLGSDALDDFDLDMLGSDALDDFDLDMLGSDALDDFDLDMLINSRSSGSPKKKRKVGSQ
YLPDTDDRHRIEEKRKRTYETFKSIMKKSPFSGPTDPRPPPRRIAVPSRSSASVPKPAPQPYPFTSSLSTI
NYDEFPTMVFPSGQISQASALAPAPPQVLPQAPAPAPAPAMVSALAQAPAPVPVLAPGPPQAVAPPAPK
PTQAGEGTLSEALLQLQFDDEDLGALLGNSTDPAVFTDLASVDNSEFQQLLNQGIPVAPHTTEPMLMEYP
EAITRLVTGAQRPPDPAPAPLGAPGLPNGLLSGDEDFSSIADMDFSALLGSGSGSRDSREGMFLPKPEA
GSAISDVFEGREVCQPKRIRPFHPPGSPWANRPLPASLAPTPTGPVHEPVGSLTPAPVPQPLDPAPAVTP
EASHLLEDPDEETSQAVKALREMADTVIPQKEEAAICGQMDLSHPPPRGHLDELTTTLESMTEDLNLDSP
LTPELNEILDTFLNDECLLHAMHISTGLSIFDTSLFGSPKKKRKVDYKDDDDK

**NMS-SpdCas9-VP64**: amino acid sequence; NMS- eNRF2, MRTF-A, STAT1, Streptococcus pyogenes Cas9 (D10A, H840A), Nucleoplasmin Nuclear Localization Sequence, Glysine-Serine Linker Sequence, VP64, SV40 Nuclear Localization Sequence,1 X “Flag” Epitope

MDYKDDDDKPKKKRKVTGSDALYFDDCMQLLAQTFPFVDDNESGGGSGGSGSSQDIEQVWEELLSIPE
LQCLNIENDKLVESGSSSQQMDDLFDILIQSGEISADFKEPPSLPGKEKPSPKTVCGSPLAAQPSPSAELP
QAAPPPPGSPSLPGRLEDFLESSTGLPLLTSGHDGPEPLSLIDDLHSQMLSSTAILDHPPSPMDTSELHFV
PEPSSTMGLDLADGHLDSMDWLELSSGGPVLSLAPLSTTAPSLFSTDFLDGHDLQLHWDSSGSEVHPSR
LQTTDNLLPMSPEEFDEVSRIVGSVEFDSSRALSGGGSGGSGSDKKYSIGLAIGTNSVGWAVITDEYKVP
SKKFKVLGNTDRHSIKKNLIGALLFDSGETAEATRLKRTARRRYTRRKNRICYLQEIFSNEMAKVDDSFFH
RLEESFLVEEDKKHERHPIFGNIVDEVAYHEKYPTIYHLRKKLVDSTDKADLRLIYLALAHMIKFRGHFLIEG
DLNPDNSDVDKLFIQLVQTYNQLFEENPINASGVDAKAILSARLSKSRRLENLIAQLPGEKKNGLFGNLIAL
SLGLTPNFKSNFDLAEDAKLQLSKDTYDDDLDNLLAQIGDQYADLFLAAKNLSDAILLSDILRVNTEITKAPL
SASMIKRYDEHHQDLTLLKALVRQQLPEKYKEIFFDQSKNGYAGYIDGGASQEEFYKFIKPILEKMDGTEE
LLVKLNREDLLRKQRTFDNGSIPHQIHLGELHAILRRQEDFYPFLKDNREKIEKILTFRIPYYVGPLARGNSR
FAWMTRKSEETITPWNFEEVVDKGASAQSFIERMTNFDKNLPNEKVLPKHSLLYEYFTVYNELTKVKYVT
EGMRKPAFLSGEQKKAIVDLLFKTNRKVTVKQLKEDYFKKIECFDSVEISGVEDRFNASLGTYHDLLKIIKD
KDFLDNEENEDILEDIVLTLTLFEDREMIEERLKTYAHLFDDKVMKQLKRRRYTGWGRLSRKLINGIRDKQ
SGKTILDFLKSDGFANRNFMQLIHDDSLTFKEDIQKAQVSGQGDSLHEHIANLAGSPAIKKGILQTVKVVDE
LVKVMGRHKPENIVIEMARENQTTQKGQKNSRERMKRIEEGIKELGSQILKEHPVENTQLQNEKLYLYYL
QNGRDMYVDQELDINRLSDYDVDAIVPQSFLKDDSIDNKVLTRSDKNRGKSDNVPSEEVVKKMKNYWRQ
LLNAKLITQRKFDNLTKAERGGLSELDKAGFIKRQLVETRQITKHVAQILDSRMNTKYDENDKLIREVKVITL
KSKLVSDFRKDFQFYKVREINNYHHAHDAYLNAVVGTALIKKYPKLESEFVYGDYKVYDVRKMIAKSEQEI
GKATAKYFFYSNIMNFFKTEITLANGEIRKRPLIETNGETGEIVWDKGRDFATVRKVLSMPQVNIVKKTEVQ
TGGFSKESILPKRNSDKLIARKKDWDPKKYGGFDSPTVAYSVLVVAKVEKGKSKKLKSVKELLGITIMERS
SFEKNPIDFLEAKGYKEVKKDLIIKLPKYSLFELENGRKRMLASAGELQKGNELALPSKYVNFLYLASHYEK
LKGSPEDNEQKQLFVEQHKHYLDEIIEQISEFSKRVILADANLDKVLSAYNKHRDKPIREQAENIIHLFTLTN
LGAPAAFKYFDTTIDRKRYTSTKEVLDATLIHQSITGLYETRIDLSQLGGDKRPAATKKAGQAKKKKSGGG
SGGSGSGSDALDDFDLDMLGSDALDDFDLDMLGSDALDDFDLDMLGSDALDDFDLDMLGSPKKKRKVD
YKDDDDK

**NMS-SadCas9 (Used in AAV)**: amino acid sequence; NMS- eNRF2, MRTF-A, STAT1, Streptococcus pyogenes Cas9 (D10A, N580A), Nucleoplasmin Nuclear Localization Sequence, Glysine-Serine Linker Sequence,1 X “Flag” Epitope

MSDALYFDDCMQLLAQTFPFVDDNESGGGSGGSGSSQDIEQVWEELLSIPELQCLNIENDKLVESGSSS
QQMDDLFDILIQSGEISADFKEPPSLPGKEKPSPKTVCGSPLAAQPSPSAELPQAAPPPPGSPSLPGRLE
DFLESSTGLPLLTSGHDGPEPLSLIDDLHSQMLSSTAILDHPPSPMDTSELHFVPEPSSTMGLDLADGHLD
SMDWLELSSGGPVLSLAPLSTTAPSLFSTDFLDGHDLQLHWDSSGSEVHPSRLQTTDNLLPMSPEEFDE
VSRIVGSVEFDSSRALSGGGSGGSGSKRNYILGLAIGITSVGYGIIDYETRDVIDAGVRLFKEANVENNEG
RRSKRGARRLKRRRRHRIQRVKKLLFDYNLLTDHSELSGINPYEARVKGLSQKLSEEEFSAALLHLAKRR
GVHNVNEVEEDTGNELSTKEQISRNSKALEEKYVAELQLERLKKDGEVRGSINRFKTSDYVKEAKQLLKV
QKAYHQLDQSFIDTYIDLLETRRTYYEGPGEGSPFGWKDIKEWYEMLMGHCTYFPEELRSVKYAYNADL
YNALNDLNNLVITRDENEKLEYYEKFQIIENVFKQKKKPTLKQIAKEILVNEEDIKGYRVTSTGKPEFTNLKV
YHDIKDITARKEIIENAELLDQIAKILTIYQSSEDIQEELTNLNSELTQEEIEQISNLKGYTGTHNLSLKAINLILD
ELWHTNDNQIAIFNRLKLVPKKVDLSQQKEIPTTLVDDFILSPVVKRSFIQSIKVINAIIKKYGLPNDIIIELARE
KNSKDAQKMINEMQKRNRQTNERIEEIIRTTGKENAKYLIEKIKLHDMQEGKCLYSLEAIPLEDLLNNPFNY
EVDHIIPRSVSFDNSFNNKVLVKQEEASKKGNRTPFQYLSSSDSKISYETFKKHILNLAKGKGRISKTKKEY
LLEERDINRFSVQKDFINRNLVDTRYATRGLMNLLRSYFRVNNLDVKVKSINGGFTSFLRRKWKFKKERN
KGYKHHAEDALIIANADFIFKEWKKLDKAKKVMENQMFEEKQAESMPEIETEQEYKEIFITPHQIKHIKDFK
DYKYSHRVDKKPNRELINDTLYSTRKDDKGNTLIVNNLNGLYDKDNDKLKKLINKSPEKLLMYHHDPQTY
QKLKLIMEQYGDEKNPLYKYYEETGNYLTKYSKKDNGPVIKKIKYYGNKLNAHLDITDDYPNSRNKVVKLS
LKPYRFDVYLDNGVYKFVTVKNLDVIKKENYYEVNSKCYEEAKKLKKISNQAEFIASFYNNDLIKINGELYR

VIGVNNDLLNRIEVNMIDITYREYLENMNDKRPPRIIKTIASKTQSIKKYSTDILGNLYEVKSKKHPQIIKKGKR
PAATKKAGQAKKKKDYKDDDDK

**MS2-modified CjCas9 gRNA expression plasmid**: U6 promoter- yellow highlight, MS2-loop- Red highlight, modified gRNA scaffold- underlined.

GAGGGCCTATTTCCCATGATTCCTTCATATTTGCATATACGATACAAGGCTGTTAGAGAGATAATTGG
AATTAATTTGACTGTAAACACAAAGATATTAGTACAAAATACGTGACGTAGAAAGTAATAATTTCTTGG
GTAGTTTGCAGTTTTAAAATTATGTTTTAAAATGGACTATCATATGCTTACCGTAACTTGAAAGTATTTC
GATTTCTTGGCTTTATATATCTTGTGGAAAGGACGAAACACCGGGAGACGGGATCCCGTCTCC**GTTT**
**TAGTCCCTGA**GCCAACATGAGGATCACCCATGTCTGCAGGGCC**GGGACTAAAATAAAGAGTTTGCG**
**GGACTCTGCGGGGTTACAATCCCCTAAAACCGC**TTTTTTT

# Uncropped Western blots from Supplementary Figures

## Western blots shown in Supplementary Figure 24

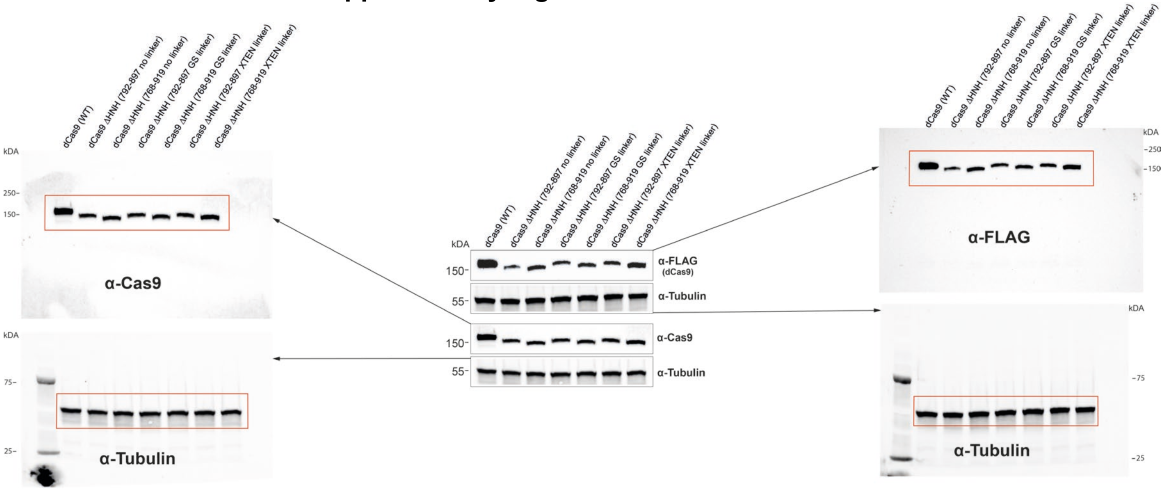


## Western blots shown in Supplementary Figure 26

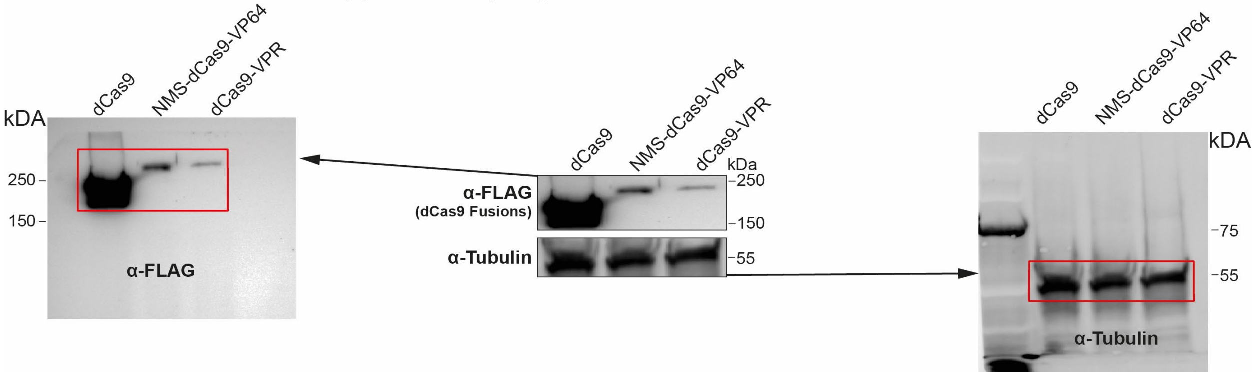

Supplement: Supplementary file 1 — All supplementary notes, supplementary references, figures, tables and uncropped gels. [file 41592_2023_2036_MOESM1_ESM.pdf]
