# Supplementary material for: Compact engineered human mechanosensitive transactivation modules enable potent and versatile synthetic transcriptional control
Source: Nat Methods. 2023 Oct 9;20(11):1716–28. doi: 10.1038/s41592-023-02036-1 (PMC10630135; doi:10.1038/s41592-023-02036-1)
Supplement: Supplementary file 2 — Reporting Summary [file 41592_2023_2036_MOESM2_ESM.pdf]

nature portfolio

Corresponding author(s): Isaac Hilton

Last updated by author(s): Aug 25, 2023

Reporting Summary

Nature Portfolio wishes to improve the reproducibility of the work that we publish. This form provides structure for consistency and transparency in reporting. For further information on Nature Portfolio policies, see our Editorial Policies and the Editorial Policy Checklist.

Statistics

For all statistical analyses, confirm that the following items are present in the figure legend, table legend, main text, or Methods section.

n/a	Confirmed	
☐	☒	The exact sample size (n) for each experimental group/condition, given as a discrete number and unit of measurement
☐	☒	A statement on whether measurements were taken from distinct samples or whether the same sample was measured repeatedly
☐	☒	The statistical test(s) used AND whether they are one- or two-sided<br>*Only common tests should be described solely by name; describe more complex techniques in the Methods section.*
☒	☐	A description of all covariates tested
☒	☐	A description of any assumptions or corrections, such as tests of normality and adjustment for multiple comparisons
☐	☒	A full description of the statistical parameters including central tendency (e.g. means) or other basic estimates (e.g. regression coefficient) AND variation (e.g. standard deviation) or associated estimates of uncertainty (e.g. confidence intervals)
☐	☒	For null hypothesis testing, the test statistic (e.g. F, t, r) with confidence intervals, effect sizes, degrees of freedom and P value noted<br>*Give P values as exact values whenever suitable.*
☐	☒	For Bayesian analysis, information on the choice of priors and Markov chain Monte Carlo settings
☒	☐	For hierarchical and complex designs, identification of the appropriate level for tests and full reporting of outcomes
☒	☐	Estimates of effect sizes (e.g. Cohen's d, Pearson's r), indicating how they were calculated

Our web collection on statistics for biologists contains articles on many of the points above.

Software and code

Policy information about availability of computer code

Data collection

All spacer sequences for SpCas9 systems were designed using the Custom Alt-R® CRISPR-Cas9 guide RNA design tool (IDT).
Illumina Hiseq3000 was used for high throughput RNA sequencing.
Nikon ECLIPSE Ti2 fluorescent microscope was used for collecting microscopic (both Phase contrast and fluorescent) images.
BioRad Chemidoc-MP was used for collecting Western Blot data.
BioRad CFX96 Real-Time PCR system with a C1000 Thermal Cycler was used for QPCR data collection.
9aa TADs were predicted using previously described online tool http://www.at.embnet.org/toolbox/9aatad/.
Sony SA3800 spectral analyzer was used for collecting CD34 surface staining data.

Data analysis	All data analysis, presented graph and statistics were performed using GraphPad Prism 9.0 software except data presented on Fig. 1c, Fig. 1f, Fig. 5a, Fig. 5d, Fig. 5e, Fig. 6a, Fig. 6b and Supplementary Fig. 11b, Supplementary Fig. 10a, Supplementary Fig. 11d, Supplementary Fig. 10e,Supplementary Fig. 12b, Supplementary Fig. 12d, Supplementary Fig. 26b, Supplementary Fig. 31j, Supplementary Fig. 33a, Supplementary Fig. 33b, Supplementary Fig. 33c, Supplementary Fig. 34a, Supplementary Fig. 34c, Supplementary Fig. 35d.<br>RNA Sequencing was performed using an Illumina Hiseq 3000 with paired end 75 base pair reads. Reads were aligned to the human genome (hg38) Gencode Release 36 reference using STAR aligner (v2.7.3a). Transcript levels were quantified to the reference genome using a Bayesian approach. Normalization was done using counts per million (CPM) method. Differential expression was done using DESeq2 (v3.5) with default parameters.<br>Analysis of DNA sequences and construction of virtual plasmid maps were performed using SnapGene.<br>For QPCR analysis CFX Manager software (Bio-Rad CFX Maestro 2.2) was used.<br>Flow Jo v10.8.1 was used for flow cytometry data analysis.<br>All data arrangement for preparation of Figures were performed on Adobe Illustrator 2021.

For manuscripts utilizing custom algorithms or software that are central to the research but not yet described in published literature, software must be made available to editors and reviewers. We strongly encourage code deposition in a community repository (e.g. GitHub). See the Nature Portfolio guidelines for submitting code & software for further information.

Data

Policy information about availability of data

All manuscripts must include a data availability statement. This statement should provide the following information, where applicable:

- Accession codes, unique identifiers, or web links for publicly available datasets
- A description of any restrictions on data availability
- For clinical datasets or third party data, please ensure that the statement adheres to our policy

Plasmids encoding MCP-MSN, MCP-NMS, MCP-eN3x9, MCP-3x 9aa TAD, MS2 stem loop modified gRNA backbone for CjCas9, and AAV vector components are available via Addgene. The RNA-sequencing data used in this study have been deposited to Gene Expression Omnibus under the accession number GSE238178. The Human reference genome GRCh38 and the mouse reference genome GRCm38 are publicly available. Source data including data used for statistical analyses are provided for all Figures and Supplementary Figures. All other datasets generated or analyzed are available upon reasonable request.

Human research participants

Policy information about studies involving human research participants and Sex and Gender in Research.

Reporting on sex and gender	N/A
Population characteristics	N/A
Recruitment	N/A
Ethics oversight	N/A

Note that full information on the approval of the study protocol must also be provided in the manuscript.

Field-specific reporting

Please select the one below that is the best fit for your research. If you are not sure, read the appropriate sections before making your selection.

☒ Life sciences ☐ Behavioural & social sciences ☐ Ecological, evolutionary & environmental sciences

For a reference copy of the document with all sections, see nature.com/documents/nr-reporting-summary-flat.pdf

Life sciences study design

All studies must disclose on these points even when the disclosure is negative.

Sample size	No statistical methods were applied to predetermine sample size. Sample sizes are similar to previous relevant publications.
Data exclusions	No data was excluded. No method was applied for data exclusion.
Replication	All experiment contains at least two biological replicates. All attempts were succesful.
Randomization	Experiments were neither randomized or blinded. Randomization is not relevant to this study.
Blinding	Data collection and analysis were not performed blind to the conditions of the experiments and outcome assessment.

Reporting for specific materials, systems and methods

We require information from authors about some types of materials, experimental systems and methods used in many studies. Here, indicate whether each material, system or method listed is relevant to your study. If you are not sure if a list item applies to your research, read the appropriate section before selecting a response.

Materials & experimental systems

n/a	Involved in the study
☐	☒ Antibodies
☐	☒ Eukaryotic cell lines
☒	☐ Palaeontology and archaeology
☒	☐ Animals and other organisms
☒	☐ Clinical data
☒	☐ Dual use research of concern

Methods

n/a	Involved in the study
☒	☐ ChIP-seq
☐	☒ Flow cytometry
☒	☐ MRI-based neuroimaging

Antibodies

Antibodies used	anti-Cas9; Diagenode #C15200216, Lot. No.#4 (1:1000 dilution), Anti-FLAG; Sigma-Aldrich #F1804, Lot. No.#SLCM4081 (1:2000 dilution), anti-β-Tubulin; Bio-Rad #12004166, Batch No.64512248, (1:1000 dilution) were used as primary antibodies for Western Blot. Anti-rabbit IgG, HRP-linked secondary Antibody; Cell Signaling Technology #7074P2, Lot. No.#28, (1:2000 dilution), Anti-mouse IgG, HRP-linked secondary Antibody; Cell Signaling Technology #7076S, Lot. No.#33 (1:2000 dilution) were were used as secondary antibodies for Western Blot.<br>SSEA-4, CST #43782, Lot. No.#2, (1:200 dilution), TRA1-60, CST #61220, Lot. No.#1, (1:200 dilution) and TRA1-81, Lot. No.#1, (1:200 dilution), CST #83321 were used for immunofluorescence microscopy. Alexa Flour 488 goat anti-mouse IgM ; Invitrogen, #A21042, Lot No.# 2306815 (1:500 dilution) and Alexa Flour 488 goat anti-mouse IgG3 ; Invitrogen, #A21151, Lot No.# 2311808 (1:500 dilution) were used as secondary antibodies for immunofluorescence microscopy.<br>CD34-PE; Invitrogen, #MA1-10205, Clone # QBEND/10, Lot. No.#YA3803041A was used (20 µL/100,000 cells) for surface staining of CD34 in HEK293T cells.<br>H3K4me3 antibody; Epicypher, #13-0041, Lot. No.#21337004-12 (0.5 µg/condition), H3K27ac antibody ;Epicypher, #13-0045, Lot. No.#22242006-81 (0.5 µg/condition) were used for CUT&RUN assay.
Validation	Cas9 : https://www.diagenode.com/en/p/crispr-cas9-monoclonal-antibody-4g10-100-ug. Also validated in "Systematic comparison of CRISPR-based transcriptional activators uncovers gene-regulatory features of enhancer-promoter interactions. Nucleic Acids Res (2022) by Wang, K. et al."<br>FLAG : https://www.sigmaaldrich.com/US/en/product/sigma/f1804. Also validated in "Systematic comparison of CRISPR-based transcriptional activators uncovers gene-regulatory features of enhancer-promoter interactions. Nucleic Acids Res (2022) by Wang, K. et al."<br>β-Tubulin : https://www.bio-rad.com/en-us/sku/12004166-hfab-rhodamine-anti-tubulin-primary-antibody-40-ul?ID=12004166. Also validated in "Systematic comparison of CRISPR-based transcriptional activators uncovers gene-regulatory features of enhancer-promoter interactions. Nucleic Acids Res (2022) by Wang, K. et al."<br>Anti-rabbit IgG, HRP-linked secondary Antibody : https://www.cellsignal.com/products/secondary-antibodies/anti-rabbit-igg-hrp-linked-antibody/707. Also validated in "Systematic comparison of CRISPR-based transcriptional activators uncovers gene-regulatory features of enhancer-promoter interactions. Nucleic Acids Res (2022) by Wang, K. et al."<br>Anti-mouse IgG, HRP-linked secondary Antibody: https://www.cellsignal.com/products/secondary-antibodies/anti-mouse-igg-hrp-linked-antibody/7076. Also validated in "Systematic comparison of CRISPR-based transcriptional activators uncovers gene-regulatory features of enhancer-promoter interactions. Nucleic Acids Res (2022) by Wang, K. et al."<br>SSEA4, TRA1-60 and TRA1-81 : https://media.cellsignal.com/pdf/9656.pdf. Further validated in this study- Liberski, A.R. et al. (2013) J Proteome Res 12, 3233-45.<br>Alexa Flour 488 goat anti-mouse IgM : https://www.thermofisher.com/antibody/product/Goat-anti-Mouse-IgM-Heavy-chain-Cross-Adsorbed-Secondary-Antibody-Polyclonal/A-21042<br>Alexa Flour 488 goat anti-mouse IgG3 : https://www.thermofisher.com/antibody/product/Goat-anti-Mouse-IgG3-Cross-Adsorbed-Secondary-Antibody-Polyclonal/A-21151<br>CD34: https://www.thermofisher.com/order/genome-database/dataSheetPdf?producttype=antibody&productsubtype=antibody_primary&productId=MA1-10205&version=300<br>H3K4me3 antibody (Epicypher, #13-0041): https://www.epicypher.com/products/epigenetics-reagents-and-assays/cutana-chic-cut-and-run-kit. This antibody was included as a positive control in the Epicypher CUTANA ChIC/CUT&RUN Kit (Epicypher, #14-1048).<br>H3K27ac antibody (Epicypher, #13-0045): https://www.epicypher.com/products/antibodies/snap-chip-certified-antibodies/histone-H3K27ac-antibody-snap-chip-certified. Also validated in "Systematic comparison of CRISPR-based transcriptional activators uncovers gene-regulatory features of enhancer-promoter interactions. Nucleic Acids Res (2022) by Wang, K. et al."

Eukaryotic cell lines

Policy information about cell lines and Sex and Gender in Research

Cell line source(s)	HEK293T (ATCC, CRL-11268), HeLa (ATCC, CCL-2), A549 (ATCC, CCL-185), SK-BR-3 (ATCC, HTB-30), U2OS (ATCC, HTB-96), HCT116 (ATCC, CRL-247), K562 (ATCC, CRL-243), CHO-K1 (ATCC, CCL-61), ARPE-19 (ATCC, CRL-2302), HFF (ATCC, CRL-2429),

	Jurkat-T (ATCC, TIB-152), Neuro-2A (ATCC, CCL-131), and hTERT-MSC (ATCC, SCRC-4000) cells were purchased from American Type Cell Culture (ATCC, USA). NIH3T3 cells were a kind gift from Dr. Caleb Bashor's lab and similar to ATCC, CRL-1658.
Authentication	Cell lines from ATCC were authenticated by STR analysis.
Mycoplasma contamination	No Mycoplasma contamination was observed.
Commonly misidentified lines (See ICLAC register)	None.

Flow Cytometry

Plots

Confirm that:

☒ The axis labels state the marker and fluorochrome used (e.g. CD4-FITC).

☒ The axis scales are clearly visible. Include numbers along axes only for bottom left plot of group (a 'group' is an analysis of identical markers).

☒ All plots are contour plots with outliers or pseudocolor plots.

☒ A numerical value for number of cells or percentage (with statistics) is provided.

Methodology

Sample preparation	Up to 100,000 Human Primary T cells were analyzed. All samples from respective groups were prepared similarly. 72 h post-transduced human primary T cells were processed using the Annexin V: PE Apoptosis Detection Kit according to manufacturer's instructions. For CD34 surface staining single HEK293T Single cell suspensions were washed with complete media and then with 0.22 um filtered 1X FACS Buffer (1% BSA in 1X PBS). Next, cells were incubated with CD34-PE antibody (20 µL/106 cells) or IgG-PE isotype antibody in 1X FACS Buffer for 30 mins. Stained cell fluorescence intensity was measured using a Sony SA3800 spectral analyzer.
Instrument	Sony SA3800 Spectral Snalyzer and Flow Cytometer
Software	Flow Jo v10.8.1
Cell population abundance	Live cell represents >75% of total collected event . EGFP-positive events represent between 1% and 50% of the total human primary T cell population.
Gating strategy	To assess the CD34 expression in EGFP positive MCP-effector transfected cells, single cells were gated based on EGFP expression. First cell debris and doublets were excluded with the FSC-A/SSC-A dot plot, followed by FSC-H/FSC-A dot plot. Events were excluded with intensity <1X 10-2, using FSC-A/PE-A dot plots. The CD34 surface marker-positive cells were gated using unstained or isotype controls. For cells transfected with an EGFP expressing effector construct, similar gating strategy was used to select singles cells as above and then events were excluded with intensity <1X 10-2, using PE-A/EGFP-A dot plots, then EGFP expressing cells were analyzed for CD34 surface marker expression using unstained or isotype controls.For primary T cells, events were plotted by FSC-A and SSC-A, and the gate "Live cells" was drawn to include live cells and exclude potential debris/dead cells. From the "Live cells" population, events were plotted by FSC-H and FSC-A to gate for population "Single Cells". From the "Single Cells" population, events were plotted to show EGFP-positive signal and were subsequently gated in the "EGFP-positive cells" population. This population was then gated for PE:Annexin V signal in the x-axis and 7-AAD signal in the y-axis, to gate for four different cell populations: Early apoptosis/ Membrane integrity is present (PE+ , 7-AAD-), Late apoptosis/Already dead (PE+ , 7-AAD+), Cells with damaged membrane (PE- , 7-AAD+), and Viable cells/ No measurable apoptosis (PE- , 7-AAD-). Gates used are shown in Supplementary Figure 33 and source data for Supplementary Figure 33.

☒ Tick this box to confirm that a figure exemplifying the gating strategy is provided in the Supplementary Information.
